# Synthetic data generation methods for longitudinal and time series health data: a systematic review

**DOI:** 10.1186/s12911-025-03326-8

**Published:** 2025-12-24

**Authors:** Marko Miletic, Murat Sariyar

**Affiliations:** Bern University of Applied Sciences, Höheweg 80, Bern, Biel/Bienne, CH-2502 Switzerland

**Keywords:** Synthetic data generation, Temporal health data, Systematic review, Data sharing, Longitudinal patient data, Privacy-preserving data publishing

## Abstract

**Background:**

Synthetic data generation (SDG) has emerged as a critical enabler for data-driven healthcare research, offering privacy-preserving alternatives to real patient data. Temporal health data – ranging from physiological signals to electronic health records (EHRs) – pose unique challenges for SDG due to their complexity, irregularity, and clinical sensitivity.

**Objective:**

This review systematically examines SDG methods for longitudinal and time-series health data. Its aims are to (1) propose a lightweight taxonomy to support orientation across the SDG landscape along five structural dimensions, (2) characterize the major synthesis techniques and their alignment with temporal structures and data modalities, and (3) synthesize the utility and privacy evaluation strategies used in practice.

**Methods:**

A systematic literature review was conducted following PRISMA guidelines across four major databases (ACM, arXiv, IEEE Xplore, Europe PMC) for publications from 2017 to 2025. Eligible studies proposed or applied SDG techniques to healthcare-relevant temporal data with sufficient methodological transparency. Structured data extraction and thematic analysis were used to identify modeling trends, evaluation metrics, and domain-specific requirements, complemented by a comparative synthesis of SDG methods.

**Results:**

A total of 115 studies were included. Deep generative models – especially Generative Adversarial Networks (GANs), Autoencoders (AEs), and diffusion-based methods – dominate the field, with increasing adoption of autoregressive and hybrid simulation approaches. Event-based EHR data are most commonly targeted, while continuous and irregular time series remain underexplored. Utility evaluations vary widely, with strong emphasis on descriptive statistics and predictive performance, but limited attention to inferential validity and clinical realism. Privacy assessments are sparse and inconsistently reported: only 30% of studies included any metric, and just around 6% implemented differential privacy (DP), often without parameter disclosure. This limited adoption may reflect technical challenges, limited expertise, and the absence of regulatory incentives.

**Conclusions:**

Synthetic temporal data play an increasingly vital role across clinical prediction, public health modeling, and Artificial Intelligence (AI) development. However, SDG research remains fragmented in terminology, evaluation practices, and privacy safeguards. Responsible-AI considerations – such as fairness, transparency, and trust – along with evidence on clinical adoption remain underexplored but are critical for future integration. This review provides a unified conceptual and methodological framework to guide future research, standardization efforts, and interdisciplinary collaboration for responsible, effective use of synthetic health data.

**Supplementary Information:**

The online version contains supplementary material available at 10.1186/s12911-025-03326-8.

## Introduction

Medical data is inherently complex and multifaceted, comprising both static variables, such as demographics, comorbidities, and baseline clinical characteristics, and time-varying measurements collected through longitudinal follow-ups, continuous monitoring, or episodic clinical encounters. These temporal data may be structured as balanced (e.g., regularly spaced observations across subjects) or unbalanced sequences, where timing and frequency vary between individuals. Additionally, the mode of data collection introduces further heterogeneity: prospective studies often follow standardized protocols, while retrospective sources like electronic health records (EHRs) tend to be irregular, incomplete, and shaped by routine clinical workflows. This results in challenges such as informative missingness, irregular sampling intervals, and heterogeneous sequence lengths [1].

The generation and use of synthetic data have emerged as a powerful response to longstanding challenges in data-driven research and development, particularly within the healthcare domain [2]. Synthetic data refers to artificially generated data that mimics the statistical properties, structure, and patterns of real-world datasets [3]. It is increasingly used to circumvent barriers such as restricted data access, patient privacy concerns, data scarcity, and ethical limitations associated with real medical datasets. By offering a safe, privacy-preserving alternative, synthetic data enables the development, testing, and validation of machine learning models without direct exposure to sensitive information. Furthermore, it supports data sharing and reproducibility in research contexts where access to real-world clinical data remains limited or infeasible [4].

Within the broader realm of temporal data, time series and longitudinal datasets represent structurally distinct yet conceptually overlapping formats. Time-series data are typically characterized by high-frequency, uniformly spaced observations, such as continuous monitoring of physiological signals like electrocardiography (ECG) or glucose levels. In contrast, longitudinal data consist of lower-frequency, often irregular measurements collected over extended periods, such as routine clinical visits or periodic follow-up assessments. Despite their differences, both formats involve sequential dependencies, dynamic temporal patterns, and potentially nested or hierarchical structures. These characteristics pose significant challenges for synthetic data generation (SDG), particularly for models originally designed for static, cross-sectional tabular data [5]. Accurately capturing such temporal complexity requires specialized generative approaches that can account for variable sampling rates, temporal correlations, and subject-specific trajectories.

Generative approaches for temporal health data now include Generative Adversarial Networks (GANs), Autoencoders (AEs), diffusion models, autoregressive architectures, and hybrids [6]. Applications range from bias testing and robust model training to high-fidelity clinical simulation, exploratory stress-testing, and rapid prototyping. To navigate this diversity, it is useful to distinguish between the primary goals driving synthetic data use:

### Training and testing

Synthetic data are used to improve model robustness by exposing algorithms to a wide range of input scenarios. The focus is not on full realism but on structural variety and functional representativeness–diverse, well-labeled, and systematically generated datasets that support generalization, fairness testing, and bias detection.

### High-fidelity modeling

These datasets aim to closely replicate real-world statistical distributions, temporal dynamics, and interdependencies. They are essential for applications that require trust in modeled patient trajectories, such as clinical decision support (CDS), in silico trials, or digital twin simulations.

### Exploratory simulation

Built on abstracted or stylized assumptions, this category supports theoretical exploration, stress-testing, and rare-event simulation. Often used when empirical data are unavailable or incomplete, such data allow researchers to probe system limits, simulate hypothetical policies, or explore counterfactuals.

### Prototyping and design

Synthetic data are increasingly used in early-stage development to rapidly test User Interface/User Experience (UI/UX) concepts, software logic, and data flow integrations. The emphasis is on low-fidelity plausibility rather than realism, enabling iterative workflows without privacy concerns.

In addition to such broad issues, it is essential to clarify a number of terms that are often treated as self-evident but are applied with considerable variation across different contexts. Concepts such as event-based, time series, longitudinal, or dynamic data are frequently used interchangeably or inconsistently, which creates confusion and hampers effective communication between disciplines and use cases. This underscores the need for some degree of conceptual standardization or at least greater terminological precision. While a single review paper cannot establish definitive norms, it can take an important first step by offering a structured overview of the field. Precisely because it surveys a broad range of approaches, a review is better positioned to propose a preliminary taxonomy than individual studies that are narrowly focused on specific technical challenges. By mapping the landscape and highlighting patterns of usage, such a paper can lay the groundwork for a more coherent and interoperable understanding of key data categories.

## Rationale

Despite a growing body of literature on SDG in healthcare, our review was conducted to address enduring and critical gaps in terminological clarity, methodological integration, and cross-modality synthesis, particularly within the domain of temporal health data. Prior reviews have offered valuable contributions by cataloging generative models, identifying use cases, and highlighting challenges in fidelity, utility, and privacy preservation [1–3]. However, none have presented a comprehensive and conceptually unified synthesis that spans the full range of temporal data modalities. These include continuous physiological signals, irregularly sampled EHRs, imaging-based time series, and event-driven clinical records. Moreover, existing works have not developed a shared methodological and conceptual foundation that would enable meaningful interpretation and comparison of generative approaches across these diverse contexts.

Most existing reviews focus either on specific data types, such as medical images, tabular records, or longitudinal EHR sequences, or on particular algorithmic families, like GANs and AEs. For example [3], provides broad overviews of deep learning-based SDG methods and emphasize the technical challenges inherent in modeling temporal health data. However, these analyses do not present a unified cross-modal structure for comparing approaches. In contrast [7] and [8], focus more narrowly on synthetic longitudinal health records and medical time series. While these studies offer valuable insights – such as highlighting that only a small subset of the literature evaluates utility, fidelity, and privacy in tandem – they also underscore the lack of comprehensive, multidimensional evaluation frameworks, which hinders reproducibility, comparability, and interoperability in synthetic data research for healthcare.

Our review directly addresses these gaps by introducing a methodological map that situates SDG techniques within a unified framework for temporal health data. It proposes a structured taxonomy to reconcile varying terminologies and modeling assumptions across fields such as biomedical informatics, machine learning, and privacy engineering. Beyond algorithmic performance, the review evaluates how well current methods preserve temporal integrity, handle data irregularities, and mitigate privacy risks [9]. By adhering to PRISMA (Preferred Reporting Items for Systematic reviews and Meta-Analyses) guidelines, it ensures transparency and rigor in study selection and synthesis [10]. Through this integrative approach, the review contributes a foundation for future benchmark development and interdisciplinary collaboration [11]. By focusing explicitly on temporal health data and offering conceptual coherence across modalities and methods, it complements prior work while addressing the need for a unified and practical reference in the evolving landscape of SDG [12].

### Objectives

We pursue three central research questions (RQs) in this review:

#### RQ1

What form could a lightweight taxonomy take to systematically organize SDG methods and support quick orientation across application scenarios?

#### RQ2

What are the key SDG methods for different types of temporal medical data, and how do these methods align with temporal structures and data modalities?

#### RQ3

What are the principal metrics used to evaluate the utility and privacy of these SDG methods?

These objectives are designed to provide both methodological depth and practical orientation in a complex and rapidly evolving research field. The first research question (RQ1, discussed in *Lightweight Taxonomy for SDG in Temporal Healthcare Contexts*) introduces a lightweight taxonomy – a practical framework to help researchers and practitioners navigate the SDG landscape by organizing methods along five structural dimensions. The second research question (RQ2, explored in *Synthesis Techniques and Their Alignment with Temporal Structures and Data Modalities*) systematically identifies and characterizes the major SDG methods, showing how they are distributed across temporal data types and data modalities. It further highlights the dimensions by which these methods can be compared, including model class, generative architecture, and strategies for handling temporal irregularity and event sequences. The third question (RQ3, addressed in *Utility and Privacy Metrics*) examines evaluation practices, focusing on metrics for utility and privacy, which are critical for assessing real-world readiness.

## Methods

We conducted a systematic literature review in accordance with the PRISMA [10] guidelines to identify, assess, and synthesize studies focused on SDG methods for temporal health data. The review aimed to address the three core research questions. Our review is intentionally methods focused. While we summarize application areas, the included studies predominantly evaluate SDG offline (or in shadow-mode) and rarely report the operational specifics required for prospective, in-workflow use (governance pathways, EHR/IT integration, safety monitoring, feedback loops). This pattern is expected given the usual division of labor between algorithmic development and applied clinical evaluation. Accordingly, we highlight deployment-adjacent progress, such as privacy safeguards, while noting that clinical adoption remains an open question. A dedicated, clinically oriented investigation focused on implementation and real-world usage is warranted and complementary to this method-centric review.

### Eligibility criteria

To ensure the inclusion of studies relevant to the modeling, evaluation, and application of SDG methods in healthcare-related temporal data, we developed a set of four eligibility criteria aligned with our research objectives (see Table 1). Studies were included if they:

1. Proposed or used SDG or augmentation methods to produce fully or partially synthetic datasets.
2. Included temporal modeling, defined as datasets with at least one variable measured across time and analyzed using time-aware methods.
3. Focused on healthcare-relevant variables, including clinical measurements, diagnoses, physiological signals, or treatment data.
4. Provided sufficient methodological transparency to allow assessment of the SDG technique and its evaluation, including utility and/or privacy aspects.

We excluded studies involving deterministic simulations, theoretical models without implementation, or data lacking sequential structure. Both proprietary and open-source methods were considered, provided methodological detail was sufficient. While privacy-preserving mechanisms were not required for inclusion, studies addressing privacy risks or trade-offs were noted. We limited the review to English-language publications in scholarly formats, including peer-reviewed journal articles, conference papers, preprints, and book chapters published between 2017 and 2025.

**Table 1:** The following set of eligibility rules was used to filter relevant publications based on the screening workflow illustrated in Fig. 1

Number	Criterion	Description	Examples of exclusions
1	SDG or Augmentation	The study must use a generative or augmentation method to produce either (a) fully synthetic datasets, or (b) real data augmented with clearly identifiable synthetic samples. Outputs must support privacy evaluation, data sharing, or model training.	Rule-based or deterministic simulations; studies lacking generative or augmentation components; theoretical models without empirical implementation.
2	Temporal Modeling	The dataset must contain at least one variable measured across multiple time points, with explicit modeling of temporal dynamics (e.g., time-aware architectures, sequence learning, or irregular sampling).	Aggregated or cross-sectional data; repeated measures without modeling temporal structure; datasets with timestamps but no sequential modeling.
3	Healthcare-relevant variables	The input or output variables must reflect the structure and clinical relevance of longitudinal health data, such as diagnoses, clinical measurements, medication events, or physiological time series.	Variable sets unrelated to healthcare contexts (e.g., financial time series or abstract signals without clinical relevance).
4	Transparent Methodology and Evaluation	Both proprietary and open-source methods were eligible, provided they were described in sufficient detail to allow assessment of temporal modeling and SDG, including use of benchmarking frameworks and reporting of utility metrics; privacy mechanisms were not required for inclusion.	Methods lacking architectural detail and transparency; No qualitative or quantitative evaluation of utility and/or privacy; Cherry-picked performance without comparison.

### Information sources and search

We searched four databases: ACM Digital Library, arXiv, IEEE Xplore, and Europe PMC. These sources were selected for their comprehensive coverage of computer science, biomedical research, and interdisciplinary publications at the intersection of artificial intelligence and healthcare. The search spanned publications from January 1, 2017 to April 1, 2025.

Search terms were constructed around three core conceptual axes:

#### Data generation tasks

“synth*”, “augment*”, “generat*”.

#### Temporal data structures

“longitudinal”, “time series”, “event”, “history”.

#### Healthcare context

“EHR”, “health record”, “electronic health record”, “medical”, “medicine”, “clinical”, “healthcare”.

Wildcard operators (e.g., *synth*) were used to capture variations of root terms. Queries were adapted to each database’s indexing syntax and applied to titles and abstracts. Where possible, publication date filters were applied to restrict results to the defined date range. A full list of database-specific search strings and results is shown in Table 2.

**Table 2:** The databases and search terms used

Database	Search terms	Search Result count
ACM Digital Library	“query”: {Title: (synth* OR augment* OR generat*) AND Title: (longitudinal OR “time series” OR time\-series OR event OR history OR EHR OR “health record” OR “electronic health record”) AND Title: (medical OR medicine OR clinical OR healthcare) OR Abstract: (synth* OR augment* OR generat*) AND Abstract: (longitudinal OR “time series” OR time\-series OR event OR history OR EHR OR “health record” OR “electronic health record”) AND Abstract: (medical OR medicine OR clinical OR healthcare)} “filter”: {E-Publication Date: (01/01/2017 TO *)},{ACM Content: DL}	538
arXiv	date_range: from 2017-01-01 to 2025-12-31; include_cross_list: True; terms: AND title = synth* OR augment* OR generat*; AND title = longitudinal OR “time series” OR time\-series OR event OR history OR EHR OR “health record” OR “electronic health record”; AND title = medical OR medicine OR clinical OR healthcare; OR abstract = synth* OR augment* OR generat*; AND abstract = longitudinal OR “time series” OR time\-series OR event OR history OR EHR OR “health record” OR “electronic health record”; AND abstract = medical OR medicine OR clinical OR healthcare	523
IEEEXplore	((DocumentTitle: (synth* OR augment* OR generat*) AND DocumentTitle: (longitudinal OR “time series” OR time-series OR event OR history OR EHR OR “health record” OR “electronic health record”) AND DocumentTitle: (medical OR medicine OR clinical OR healthcare)) OR (Abstract: (synth* OR augment* OR generat*) AND Abstract: (longitudinal OR “time series” OR time-series OR event OR history OR EHR OR “health record” OR “electronic health record”) AND Abstract: (medical OR medicine OR clinical OR healthcare)))	28
Europe pmc	(TITLE: (“synth*” OR “augment*” OR “generat*”) AND TITLE: (“longitudinal” OR “time series” OR “time-series “ OR “event” OR “history” OR “EHR” OR “health record” OR “electronic health record”) AND TITLE: (“medical” OR “medicine” OR “clinical” OR “healthcare”)) AND (ABSTRACT: (“synth*” OR “augment*” OR “generat*”) AND ABSTRACT: (“longitudinal” OR “time series” OR “time-series “ OR “event” OR “history” OR “EHR” OR “health record” OR “electronic health record”) AND ABSTRACT: (“medical” OR “medicine” OR “clinical” OR “healthcare”))	379 (link to free text)

### Data capture

Structured data extraction was performed using REDCap instruments, enabling standardized capture of literature-information, method-characteristics, and method-performance-evaluation [13]. These instruments encoded fields with controlled vocabularies, conditional logic, and free-text descriptors to support structured annotation of contextual features relevant for organizing methods into a lightweight, application-oriented taxonomy (RQ1), synthesis techniques and their alignment with temporal structures and data modalities (RQ2), and reported utility and privacy evaluation metrics (RQ3). Manual review by domain-expert annotators ensured fidelity of the captured data, with interdependent fields cross-validated to reduce inconsistencies. This structured instrument-based pipeline enabled downstream aggregation, stratification, and analysis of SDG methods with respect to their utility, transparency, and applicability to healthcare-relevant temporal data.

For the “Temporal Data Type” and “Data Modality” dimensions in relation to RQ2, we additionally examined the datasets used in each publication to determine whether certain use cases are associated with standardized benchmark datasets. This manual inspection went beyond the structured information captured via REDCap instruments and allowed us to assess whether specific datasets have become de facto standards for particular modeling paradigms or application areas. By mapping each study to one of the central temporal data types and data modalities, we were able to draw more nuanced insights about model complexity, data dependency, and methodological generalizability.

## Results

**Fig. 1:**
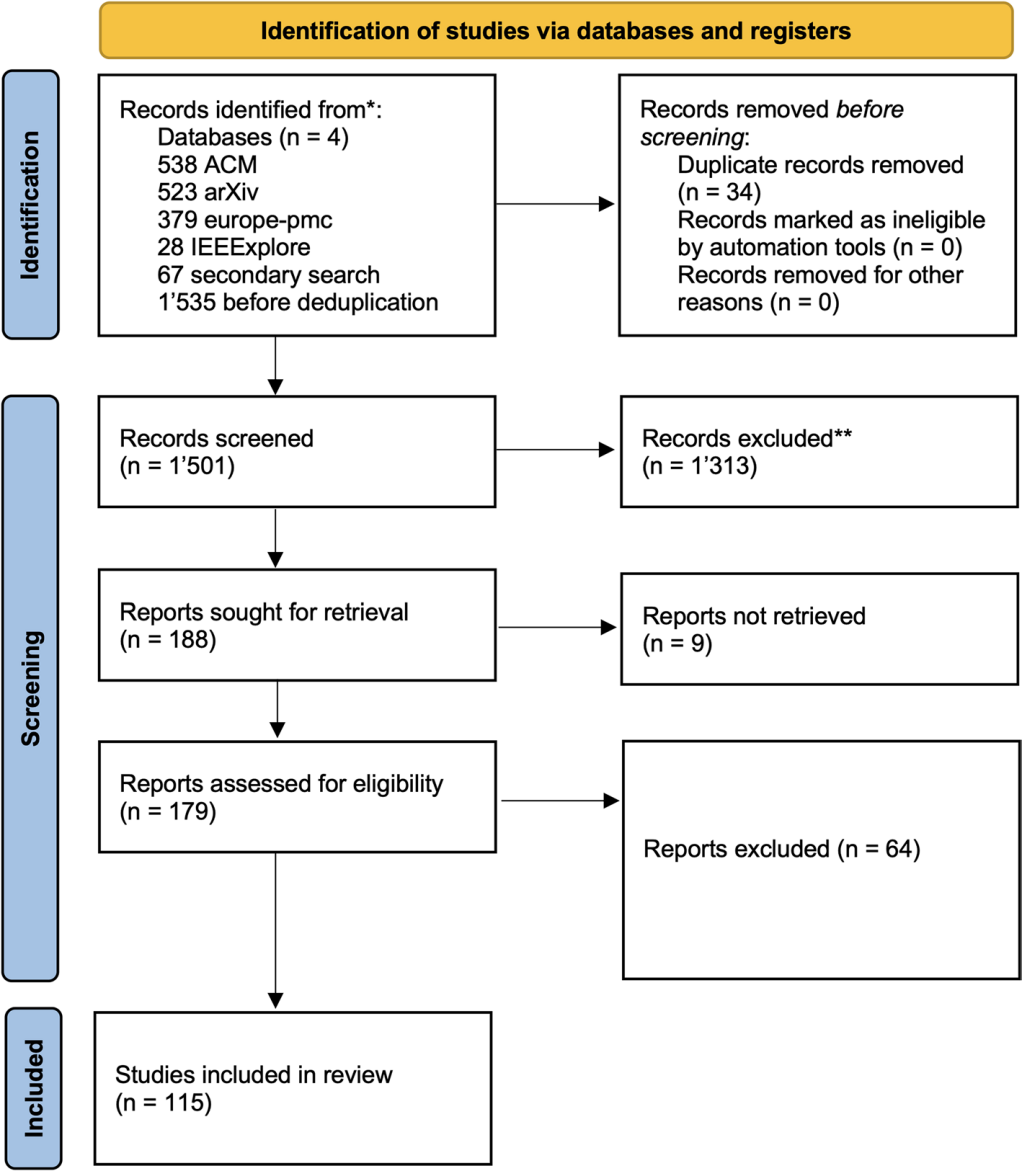
PRISMA flow diagram for the literature database and website search

The study selection process followed the PRISMA guidelines, as illustrated in Fig. 1. A total of 1,535 records were identified through systematic database and secondary searches, including 538 from ACM Digital Library, 523 from arXiv, 379 from Europe PMC, 28 from IEEE Xplore, and 67 from secondary sources. After the removal of 34 duplicates, 1’501 unique records remained for screening.

Title and abstract screening excluded 1’313 records, leaving 188 articles for full-text retrieval and eligibility assessment. Nine reports could not be retrieved (e.g., due to paywalls). Of the 179 full-text reports assessed, 64 were excluded based on predefined eligibility criteria related to the presence of SDG methods, temporal modeling, clinical relevance, or insufficient methodological transparency.

A total of 115 studies met the inclusion criteria and were included in the final synthesis, covering a wide range of SDG methods for temporal health data, including time series and longitudinal formats. Presenting all paper-level details within the main text would exceed the scope of the article without offering additional interpretive value; therefore, this information is provided in Supplementary Table S1.

### Lightweight taxonomy for SDG in temporal healthcare contexts (RQ1)

To facilitate consistent comparison and rapid orientation SDG methods for temporal health data, we propose a five-dimensional lightweight taxonomy (for a summary, see Table 3). Each dimension captures a key aspect relevant to model design, evaluation, or clinical application:

#### Temporal data type

This dimension emphasizes the temporal structure of healthcare data – particularly sampling regularity and event dependence – as key factors influencing model complexity and architectural design. By attending to these structural characteristics, the typology deliberately avoids potentially ambiguous labels like “longitudinal” or “sequential”, which often conflate data format, observation frequency, and semantic interpretation. The typology distinguishes three primary forms of temporal data:

##### C-R (Continuous – Regular sampling)

Refers to data collected at fixed, equidistant time intervals, such as Intensive Care Unit (ICU) waveform signals (e.g., ECG or electroencephalogram (EEG) signals). Here, “continuous” does not imply truly continuous time in the mathematical sense, but rather a high-resolution approximation based on dense, regular sampling. These datasets support fine-grained dynamic modeling and often underlie differential or autoregressive architectures.

##### C-IR (Continuous – Irregular sampling)

Captures data streams with variable time intervals between observations, typical of wearable sensors or intermittently recorded vital signs. Though still representing continuous underlying processes, the sampling is uneven, requiring models that handle irregular time gaps, time-awareness, and potentially informative missingness.

##### E (Event-based)

Involves timestamped but non-continuous records such as diagnostic codes, clinical procedures, or medication administrations. While these are often labeled as “events”, it is important to distinguish between observations, actions, and ontological events. A recorded diagnosis, for example, is a documentation of a clinical judgment, not necessarily an event in itself, but a representation of an event-like process.

Defining what counts as an “event” is philosophically and ontologically non-trivial. In the Basic Formal Ontology (BFO) [14], events are considered occurrents, spatiotemporal entities that unfold over time and have temporal parts. They are distinct from instantaneous observations or discrete data points, which may only reflect the result or trace of an underlying process. In contrast, the Unified Foundational Ontology (UFO) [15] emphasizes the agentive and relational structure of events, treating them as relational occurrences that involve participants, trigger conditions, and consequences. This richer structure allows for a more semantically grounded interpretation of clinical events (e.g., a “medication administration” involves a healthcare actor, an intention, a drug, and a patient) but also introduces modeling complexity. In practice, many EHR systems record event-like observations that do not cleanly map to either ontological definition. Therefore, the “E” category in this typology should be interpreted pragmatically: it includes discrete, temporally localized data entries that may signal or result from underlying clinical processes but do not necessarily qualify as fully ontological events in a strict sense.

Applying the typology of temporal healthcare data highlights how commonly used datasets align with distinct modeling demands. In the C-R (Continuous–Regular sampling) category – characterized by high-frequency, equidistant samples – datasets such as MIT-BIH Arrhythmia [16], PTB Diagnostic ECG [17], MIT-BIH [18], WISDM [19], and eICU [20] are prevalent. These support autoregressive and waveform-based modeling of physiological signals and have been used in multiple studies, including works [21–25]. Sleep-EDF [26], though primarily C-R, also includes event-based components, supporting hybrid modeling approaches. For C-IR (Continuous–Irregular sampling), datasets like PPMI [27] and PhysioNet 2012 [28] are widely used for time-aware modeling of unevenly spaced clinical or wearable data. In the E (Event-based) category, MIMIC-IV [29] dominates with over six referenced studies, followed by datasets such as eICU [20] and others with structured EHR data. Despite the prominence of these datasets, the field lacks unified benchmarking standards across temporal types, resulting in fragmented evaluation practices. Aligning SDG methods with the inherent temporal structure of each dataset remains essential for addressing the complexity of real-world healthcare data. A complete list of datasets, along with their temporal classifications and associated studies, is provided in Supplementary Table S2.

#### Data modality

This dimension highlights the primary forms of medical data on which SDG methods operate. Each modality carries distinct challenges for temporal fidelity, structural realism, and clinical utility. Aligning SDG approaches with these modality-specific requirements is critical for ensuring both technical rigor and translational relevance.

##### Physiological signals

Biosignals such as ECGs, EEGs, photoplethysmography (PPG), electromyography (EMG), and respiratory or ventilator waveforms represent some of the most widely studied modalities for SDG. These high-frequency continuous signals demand preservation of fine-grained temporal structure, waveform morphology, and physiological plausibility, including realistic noise characteristics. Synthetic signals in this category support applications in monitoring, detection of acute events, wearable-device development, and simulation of rare or privacy-sensitive conditions.

##### Medical imaging and video

Radiological images (MRI, CT, X-ray, ultrasound) and ophthalmologic modalities such as OCT, as well as video-based examinations like laryngeal endoscopy, constitute a second major category. Beyond spatial fidelity, SDG methods must address longitudinal coherence when images are captured at multiple time points, ensuring consistency in disease progression or anatomical development. Synthetic imaging and video data enable algorithm training at scale, enhance reproducibility in benchmarking, and reduce reliance on sensitive or scarce patient image archives.

##### Electronic health records (EHRs)

Structured EHR datasets, including diagnoses, medications, procedures, labs, and vitals, are often modeled as discrete event sequences or tabular time series. They combine irregular sampling with heterogeneous data types, requiring generative models to capture logical consistency (e.g., medication follows diagnosis), temporal dependencies, and population-level representativeness. Synthetic EHR data are central for predictive modeling, risk stratification, decision support, and privacy-preserving data sharing, while also serving as a foundation for simulating health services and policy scenarios.

##### Clinical trials and cohort studies

Longitudinal multimodal study datasets, including population biobanks (e.g., UK Biobank), disease-specific cohorts (e.g., ADNI, PPMI, NACC), and oncology or cardiovascular clinical trials, form a fourth domain. These sources integrate clinical, imaging, biomarker, and patient-reported outcomes across extended time horizons. SDG in this category must respect trial protocols, longitudinal outcome dependencies, and demographic heterogeneity to remain scientifically valid. Synthetic trial and cohort data enable reproducible secondary analyses, facilitate meta-research across institutions, and expand accessibility for methodological innovation without exposing sensitive participant records.

Together, these modalities illustrate the breadth of synthetic data generation in healthcare, while underscoring the importance of tailoring SDG methods to the structural and clinical demands of each data type to ensure scientifically valid, ethically responsible, and practically useful applications.

#### Synthesis technique

The landscape of SDG in temporal health research spans two major paradigms – Statistical and Deep Learning – each comprising distinct methodological families. These paradigms reflect core assumptions about how data are modeled and generated, independent of implementation details.

### Statistical models

This category includes Bayesian networks, time series models, and other probabilistic approaches that rely on explicit assumptions about dependencies and distributions. These models offer transparency, reproducibility, and efficiency, making them suitable for structured or low-dimensional datasets, especially where interpretability is critical.

### Deep learning models

Deep learning–based SDG methods cover a range of generative paradigms:

#### Generative models

aim to model the joint distribution of data either explicitly (e.g., AEs) or implicitly (e.g., GANs, diffusion models). They typically generate full samples in one pass and are well-suited for high-dimensional data such as images, dense EHR tables, or multimodal signals.

#### Autoregressive and sequence models

generate data step-by-step by factorizing the joint distribution into conditional probabilities. Examples include Transformers, Recurrent Neural Networks (RNNs), and temporal Convolutional Neural Networks (CNNs). These are ideal for event sequences, longitudinal trajectories, and irregular time series, as they preserve temporal dependencies and causal ordering.

#### Diffusion and score-based models

while part of neural generative models, stand out for their iterative denoising mechanisms and are increasingly applied to clinical time series and complex multimodal datasets.

#### Simulation-informed and hybrid models

combine domain-based rules or simulations with neural components, enabling the integration of causal logic, interpretability, and empirical flexibility, which are critical in high-stakes biomedical applications.

We include both hybrid and simulation-informed Models and other deep learning methods because they address complementary needs in synthetic health data generation. Hybrid generators deliberately fuse two recognized method categories, inheriting complementary strengths to boost realism and interpretability, whereas “other” approaches – novel architectures like transformer-contrastive pipelines – do not fit any existing category and are grouped separately for their originality, flexibility and scalability.

#### Utility evaluation

Utility evaluation assesses how well synthetic data preserve the analytical and clinical value of real datasets. For temporal health data, this involves multiple complementary dimensions:

##### Descriptive statistics

Measures how closely synthetic data reproduce key statistical properties such as distributions, correlations, and temporal dependencies. Metrics include means, standard deviations, Jensen-Shannon divergence [30], Wasserstein distance [31], and Kolmogorov-Smirnov (K-S) tests [32].

##### Predictive performance

Evaluates whether models trained on synthetic data perform comparably to those trained on real data. Common setups include Train-on-Synthetic-Test-on-Real (TSTR) and Train-on-Real-Test-on-Synthetic (TRTS), with performance measured using accuracy, F1-score, AUROC [33], AUPRC, and calibration metrics.

##### Clinical realism

Assesses whether synthetic data reflect plausible clinical scenarios, such as coherent patient trajectories and treatment pathways. Expert reviews, structured rating systems, and visual tools (e.g., Kaplan-Meier plots [34], sequence diagrams) are used.

##### Inferential utility

Tests whether synthetic data preserve statistical relationships needed for hypothesis testing or causal inference. Methods include regression comparisons, statistical significance tests, and likelihood-based evaluations.

##### Qualitative evaluations

Uses visual and expert-based assessments to check the coherence, variability, and structure of synthetic data. Techniques include waveform comparisons and dimensionality reduction plots (e.g., PCA [35], t-SNE [36], UMAP [37]).

Although both clinical realism and qualitative evaluation involve expert-informed judgments, they capture distinct yet partially overlapping dimensions of synthetic data utility. Clinical realism focuses on medical plausibility and domain coherence – that is, whether synthetic data reflect credible patient trajectories, treatment patterns, and disease progressions consistent with clinical knowledge. It is central when synthetic data are intended for applications involving clinical reasoning, workflow simulation, or decision support, where interpretability and trust are paramount. In contrast, qualitative evaluation concerns the visual and structural fidelity of synthetic data, often at a more technical or statistical level. It typically involves exploratory analyses and visual diagnostics – such as waveform overlays, temporal heatmaps, or low-dimensional embeddings (e.g., PCA, t-SNE, UMAP) – to assess whether the data exhibit expected variability, diversity, and internal consistency. While these analyses frequently overlap with descriptive evaluations and are sometimes used interchangeably, their emphasis lies in detecting generative artifacts (e.g., mode collapse, unrealistic temporal smoothness, or loss of variability) rather than assessing domain realism. Thus, qualitative evaluation complements both descriptive statistics and clinical realism by providing an intuitive, visual check of data integrity and generative soundness.

#### Privacy evaluation

This dimension distinguishes between approaches that offer formal privacy guarantees, those that rely on empirical risk evaluation, and those that propose heuristic or design-based safeguards without rigorous assessment.

##### Formal privacy guarantees

Approaches that provide mathematically provable bounds on privacy loss, most notably through frameworks such as differential privacy (DP) and related metrics. These studies define strict privacy guarantees under well-specified assumptions and adversarial models.

##### Empirical privacy evaluation

Studies that assess privacy risks a posteriori using attack simulations such as membership inference, attribute inference, and re-identification tests. While lacking formal guarantees, these methods provide practical insights into the actual leakage potential of synthetic data.

##### Design-based safeguards

Approaches that attempt to mitigate privacy risk through architectural constraints (e.g., bottlenecks in generative models) or data-level interventions (e.g., filtering rare categories), often without quantifying residual risk. These rely on intuitive reasoning rather than formal validation.

In Table S1, we annotated the strongest privacy evidence reported per study with the following precedence: Formal (e.g., implemented DP or equivalent formal mechanism, even if ε/δ was not disclosed) > Empirical (attack-based or risk tests such as membership/linkage/attribute inference or nearest-neighbor leak checks) > Design-based (heuristic safeguards like rare-category suppression, clipping, or rule filters without quantified risk) > None. “None” indicates that no technical privacy mechanism and no empirical privacy test were reported.

**Table 3:** Dimensions for lightweight taxonomy

Dimension	Categories (examples)
Temporal Data Type	Continuous Sampling (Regular - C-R, Irregular - C-IR) and Event-based Sampling (E)
Data Modality	Physiological Signals, Medical Imaging and Video, Electronic Health Records, Clinical Trials and Cohort Studies
Synthesis Technique	Statistical (e.g., Bayesian Networks), Deep Learning (e.g., Diffusion and Score-Based Models)
Utility Evaluation	Descriptive Statistics, Predictive Performance, Clinical Realism, Inferential Utility, Qualitative Evaluations
Privacy Evaluation	Formal Privacy Guarantees, Empirical Privacy Evaluation, Design-Based Safeguards

Our five dimensions are deliberately scoped to the structural properties of methods. This focus enables concise comparison across approaches without conflating model design with governance. Broader attributes such as fairness, bias, or transparency are instead cross-cutting considerations that apply to more than one single dimension. Treating these as a separate annotation layer – rather than embedding them in the taxonomy – avoids inflating the schema while ensuring that responsibilities are visible. Future extensions may formalize this into a standardized responsibility profile accompanying each taxonomy instance to capture these evaluation- and governance-level properties.

We present the taxonomy as a structured mapping rather than a hierarchical ontology. The five dimensions are faceted and orthogonal, reflecting methodological choices that often co-occur (e.g., C-IR + physiological signals + diffusion-based synthesis + empirical privacy evaluation). A single hierarchy would obscure such dependencies, whereas our scaffold makes them explicit. The added value is to (i) foreground temporal structure (C-R / C-IR / E), (ii) clearly separate design choices from evaluation criteria through the annotation layer and optional responsibility profile, and (iii) provide a compact basis for consistent reporting and comparison across data modalities.

### Synthesis techniques and their alignment with temporal structures and data modalities (RQ2)

Before detailing the synthesis techniques themselves, we first outline the relationship between temporal data types and data modalities in health research. Building on this, we then describe how the synthesis techniques align with these dimensions. The three temporal data types impose distinct modeling requirements across data modalities.

Physiological signals such as ECG [38], EEG from Sleep-EDF [26], or other high-frequency biosignals (e.g., PPG, EMG, ventilator waveforms) exemplify C-R data, where preserving fine-grained continuity, waveform morphology, and physiologic plausibility is essential. Medical imaging and video often reflect C-IR structure, as in OCT follow-ups [39] or regularly spaced video frame sequences such as BAGLS [40]; here models must ensure both temporal coherence and consistency in disease or anatomical progression. Electronic Health Records (EHRs) typically combine E-type data – diagnoses, medication administrations, or admissions and readmissions – with irregularly sampled vitals or labs, requiring generative models that maintain logical ordering, temporal dependencies, and population-level realism [41]. Clinical trials and cohort studies further integrate multiple modalities, often combining event-based clinical outcomes with irregular biomarker measurements, as in predictive tasks that jointly leverage physiological variables and discrete medication events [42, 43].

**Fig. 2:**
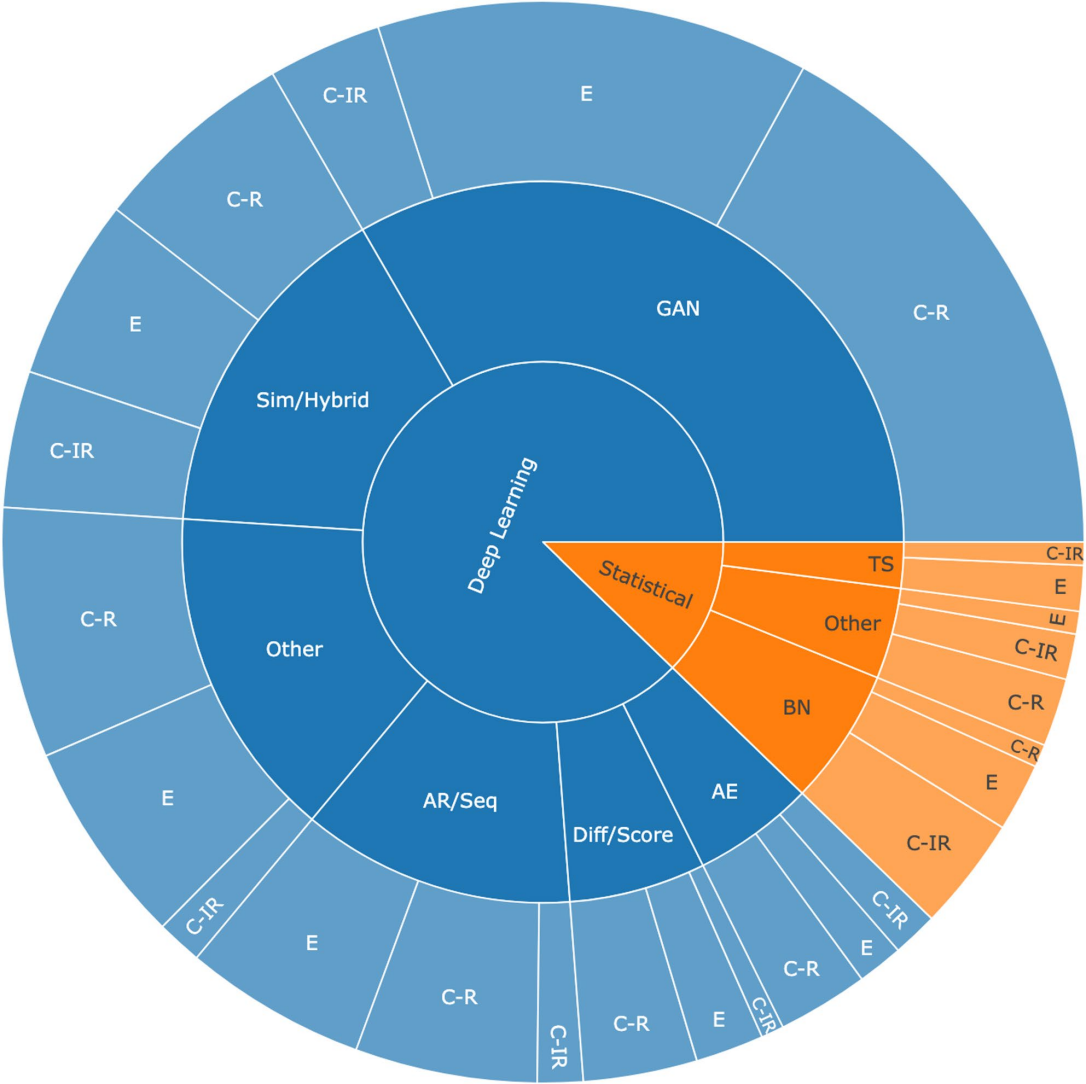
Sunburst chart illustrating the synthesis techniques employed in temporal health research. The chart organizes methods hierarchically by class, synthesis technique, and temporal data structure. For clarity, synthesis technique labels are abbreviated as follows: BN indicates Bayesian Networks; TS stands for Time-Series; GAN refers to Generative Adversarial Networks; AE stands for Autoencoder; Diff/Score represents Diffusion and Score-Based Models; AR/Seq refers to Autoregressive and Sequence Modeling Frameworks; and Sim/Hybrid denotes Simulation-Informed and Hybrid Models

With this alignment in view, the distribution of synthesis techniques across temporal types can be examined. Figure 2’s sunburst chart illustrates how different SDG methods are applied, underscoring the central role of temporal structure in shaping methodological choices. Event-based (E) data dominate overall usage, particularly among GANs (39%), autoregressive/sequence models (44%), and autoencoders (25%), reflecting the prominence of discrete clinical events such as diagnoses, procedures, or encounters in health records. Continuous-irregular (C-IR) data are most prominently modeled with Bayesian networks (56%) and simulation/hybrid approaches (26%), but also appear in autoencoders and diffusion-based methods that are well-suited to irregular sampling and informative missingness. Continuous-regular (C-R) data show strong associations with diffusion/score-based methods (56%) and GANs (51%), while remaining less common in statistical approaches like Bayesian networks (11%) and absent from time series models. Time series models, though rare, are applied exclusively to event-based (67%) and irregular (33%) data.

**Fig. 3:**
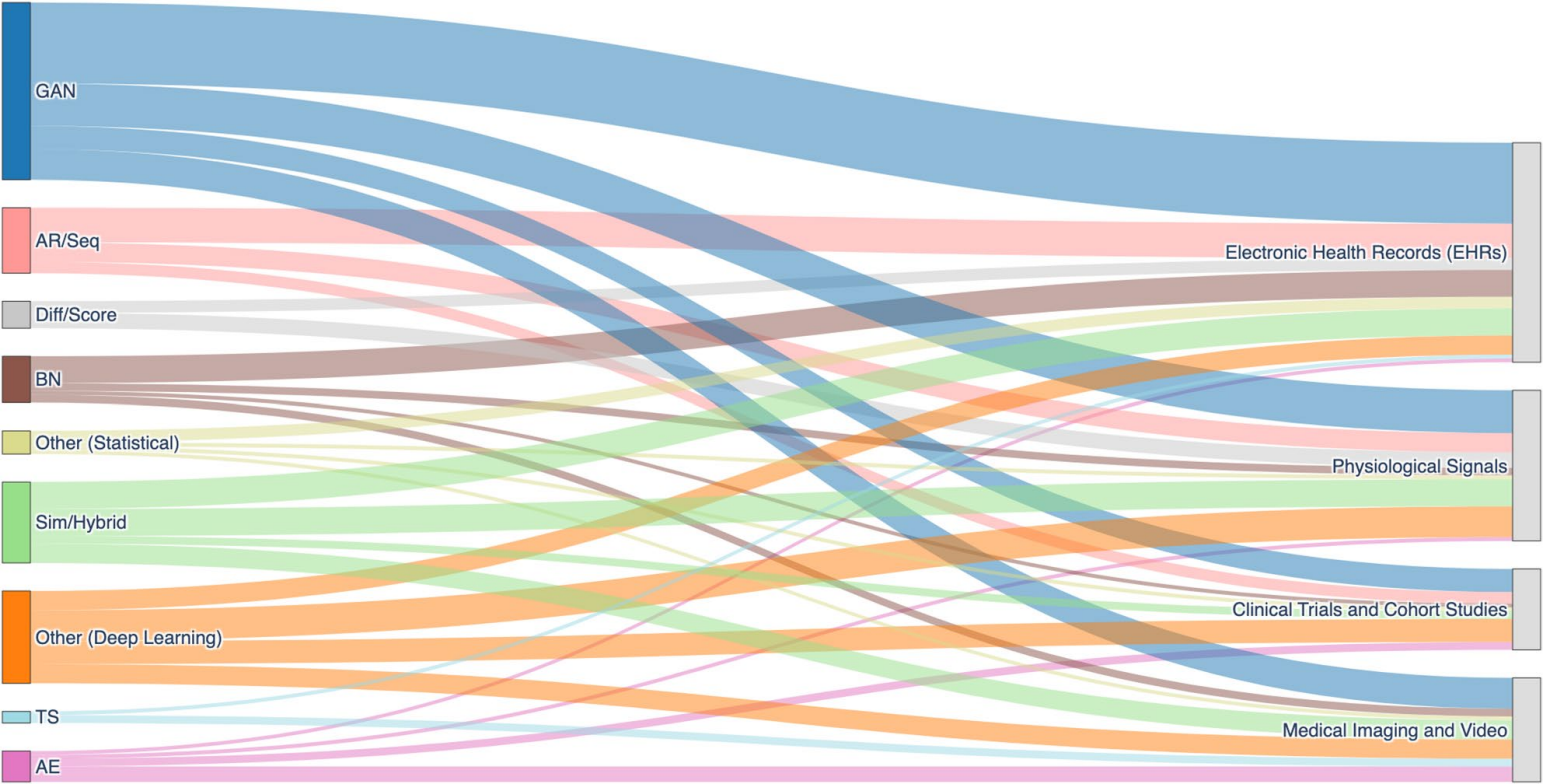
Sankey diagram illustrating the distribution of synthesis techniques across different data modalities

Figure 3 complements this by using a Sankey diagram to map synthesis techniques onto data modalities. This view highlights how methodological choices are distributed across EHRs, physiological signals, clinical trial or cohort data, and medical imaging and video, making the interplay between synthesis technique and modality explicit.

### Statistical methods

#### Bayesian networks

Bayesian network-based methods simulate temporal health data by representing medical events – such as diagnoses, treatments, or sensor readings – as interconnected nodes in a structured probabilistic graph. These models are valued for their interpretability and ability to represent cause-effect relationships and patient-specific trajectories over time. Some approaches use extended Markov models or combine causal discovery with disease simulations to model how conditions evolve (Schulz et al. [44], Ceritli et al. [45], Song et al. [46]). Others apply bootstrapping or personalized dynamics to capture heterogeneity in disease markers or sensor signals (Sood et al. [47], Dahmen and Cook [48]). These methods work well when transparent logic and clinical relevance are priorities, though they are less scalable and less flexible than deep learning models when handling complex or irregular data (Wang et al. [42], Poyraz and Marttinen [49]).

#### Time-series models

Time-series approaches model health data as sequences that unfold over time, ideal for repeated measurements or physiological signals. These models track how variables influence one another across time steps and often use hidden states or learned transitions to capture complex dynamics. For example, SrVARM combines time-based patterns and latent states to model multivariate health signals (Hsieh et al. [50]), while HyperHawkes uses a neural version of event-based modeling to simulate patient-specific event sequences and adapt to new data with minimal retraining (Dubey et al. [51]). These methods offer clear temporal structure and adaptability.

#### Other statistical methods

Beyond mainstream statistical techniques, several diverse methods synthesize temporal health data using rules, latent structures, or signal transformations. Some simulate synthetic patient histories using rule-based transitions grounded in epidemiological data (Walonoski et al. [52]), while others use tensor networks to capture joint time dependencies (Moore et al. [53]), or manipulate frequency components of physiological data for realistic augmentation (Arabi et al. [54]). Approaches like cRBMs (Fisher et al. [55]) and random forests (Afrin et al. [56]) generate realistic sequences by conditioning on past observations or predicting signal segments. Others combine metadata with patient time series (Larrea et al. [57, 58]) or use Bayesian models to track disease progression over time (Alt et al. [59]).

### Deep learning methods

#### GANs

GANs are among the most widely used deep learning methods for generating synthetic temporal health data. They are especially known for their ability to model complex and high-dimensional patient data with realistic temporal structure. Most approaches combine recurrent, convolutional, or transformer-based architectures with conditional logic to simulate patient records, often aligning outputs with diagnoses, treatments, or outcomes. For example, SC-GAN [60], SleepGAN [61], and EHR-Safe [62] apply sequence-to-sequence architectures and attention mechanisms to generate realistic clinical trajectories, while GluGAN [63] and WaveletGAN [64] use autoregressive decoding. Some models introduce novel mechanisms like dual generators (SC-GAN [60]), memory attention (SleepGAN [61]), or latent supervision (TAP-GAN [65]). Conditional GANs – like MTGAN [66] and PlethAugment [67] – enable tailored synthesis for specific medical contexts. Several approaches include privacy-preserving mechanisms (e.g., RDP-CGAN [21], Time-ADS-GAN [68], SynTEG [69], SurvivalGAN [70]). While GANs can produce highly realistic data useful for training downstream models or simulating rare conditions, they often require complex training and may suffer from challenges like temporal inconsistency or mode collapse.

#### AEs

Autoencoder-based models generate synthetic temporal data by compressing health records into latent representations and then reconstructing them. Variants such as VAEs and conditional VAEs allow flexible control over temporal patterns and patient characteristics. CR-VAE [71] incorporates Granger causality to structure variable relationships over time, while Biswal et al. [72] apply masked decoding to capture dependencies between patient visits. Other works (Bing et al. [73], Ramchandran et al. [74]) extend VAEs to account for static patient attributes and missingness, improving fairness and customization. GlOVe [40] offers physiologically meaningful latent trajectories, while Deltadahl et al. [39] integrate VAEs with stable diffusion and mixed-effects models for outcome-informed generation. Graph-based models like those by Nikolentzos et al. [75] structure EHRs as directed acyclic graphs to preserve event sequences. Transformer-based approaches (Li et al. [22, 24]) model long-range dynamics in biosignals. While AE are versatile and interpretable, they may be harder to train and less suited to highly irregular data unless specifically adapted.

#### Diffusion and score-based methods

Diffusion models are a new class of generative methods that gradually convert noise into structured data through denoising steps. In health applications, they have shown promise for synthesizing realistic time series and clinical records. SSSD-ECG [76] combines structured state space models with DiffWave [77] to model long-term ECG dynamics. Vetter et al. [78] apply structured convolutions and Ornstein-Uhlenbeck noise to reproduce neurophysiological signals. MedDiffusion [79] and TimeDiff [80] integrate the long short-term memory (LSTM) or bidirectional recurrent neural networks (BRNN) architectures to generate EHRs with support for both continuous and categorical variables, while EHRPD [81] uses predictive U-Nets and time-aware embeddings to capture visit timing and content. Kuo et al. [82] develop a 1D U-Net with linear layers to synthesize mixed-type EHRs, and TimeLDM [83] performs diffusion in a latent space encoded by transformers to capture spectral dynamics. Many models emphasize privacy (e.g [80, 82]), or domain-specific loss functions ([83]). Diffusion methods offer strong fidelity and are gaining ground over GANs in privacy-sensitive or high-precision applications, though they are computationally demanding and less interpretable.

#### Autoregressive and sequence modelling frameworks

Autoregressive models generate synthetic data step by step, using each previous observation to predict the next. This approach preserves the temporal flow of clinical data, making it ideal for modeling patient trajectories or physiological signals. Conditional LSTM and Gated Recurrent Unit (GRU) architectures have been applied to generate administrative records (Mosquera et al. [41]) and glucose time series (Zhu et al. [63]). Transformer-based methods like CEHR-GPT [84], HiSGT [85], and HALO [86] use attention mechanisms to model visit histories and intra-visit codes, sometimes with added semantic, fairness, or hierarchical constraints. Waveform-focused methods include WaveNet-style convolutional models (Wulan et al. [64]) and extrapolatable forecasting (Song et al. [38]). Others emphasize augmentation rather than generation – using smoothing, permutations, or frequency transformation (Choi and Kim [87], Alawneh et al. [88], Eyobu and Han [89]). Further innovations include fairness-aware autoregression (Tarek et al. [90]), multimodal fusion (Silva and Matos [91]), and domain-specific data augmentation (Cao et al. [92]). These models are strong in sequential fidelity but may struggle with very long sequences or maintaining privacy guarantees.

#### Simulation-informed and hybrid models

These models combine neural networks with domain-based rules, physics-inspired constraints, or probabilistic systems to reflect real-world clinical knowledge. Neural ODEs model continuous-time trajectories in systems like those proposed by Habiba et al. [93], Linial et al. [94], and Brouwer et al. [95]. Autoregressive mechanisms appear in models like LS-EHR [96] and TrialSynth [97]. Hybrid models also embed simulation logic within generative networks–e.g., GOKU-net [94] learns physiological parameters through known ordinary differential equations (ODEs), and TWIN [98] uses a module to preserve causal relationships across visits. Modular frameworks like VAMBN [99] and MultiNODE [100] use VAEs linked by Bayesian networks to model irregular longitudinal data. Other notable examples include IGNITE for personalized simulation [101], Deep-PxAF for expert-certified pipelines [102], and recurrence plot-based signal modeling (Asadi et al. [102]). These models are ideal where personalization and clinical realism are required, though they are often complex and resource intensive.

#### Other Deep learning methods

This category includes a range of deep learning approaches that don’t fall into standard families but contribute innovative strategies for health data generation or augmentation. Transformer-based models such as those by Theodorou et al. [1, 84, 86], Wang and Sun [103], and Chang et al. [104] model sequential structure across visits or signal segments. Spatiotemporal neural fields (Sørensen et al. [105]) offer fine-grained motion synthesis. Contrastive and representation learning approaches like Kallidromitis et al. [106], Wickstrøm et al. [107], and Ming et al. [108] focus on learning structure-preserving embeddings. Others focus on neural language models (PromptEHR [109], TWIN-GPT [110]), spectral augmentation (UniCL [43]), and large language model guided generation (Lee et al. [111]). These models are often used for personalized simulation, pretraining, or multimodal integration. They are highly flexible and suited for diverse tasks and data types, though typically require significant tuning and computational resources.

### Comparative synthesis across method families

Building on Table S1 and our five-dimensional taxonomy, we provide a qualitative synthesis organized by data type, evaluation priorities, privacy amenability, and interpretability.

#### GANs

Adversarial models work particularly well for C-R waveforms (e.g., ECG/EEG) and event-rich EHR when label-conditioned augmentation is needed. They routinely deliver high-fidelity samples and support flexible conditioning, with established TSTR/TRTS evaluation patterns. Their limitations are equally well known: training instability and mode collapse, degraded sequence-level coherence across long or irregular histories, and predominantly empirical (rather than formal) privacy evidence. GANs are most suitable when task-oriented augmentation and high perceptual realism are priorities and resources allow for stabilization and systematic evaluation.

#### AEs

AE-based approaches are broadly effective for E and C-IR data. They offer stable training, interpretable and controllable latent spaces, and straightforward integration of static covariates and patterns of missingness – useful for imputation, counterfactuals, and downstream inference. The trade-offs are potential over-smoothing, under-representation of rare phenomena, and realism that can lag GANs or diffusion unless decoders and priors are carefully designed. AEs are strong default models when structured latent factors, robustness, and inferential utility take precedence over absolute fidelity.

#### Diffusion and score-based models

Diffusion models excel when fidelity and mode coverage are paramount, notably for C-R signals and mixed-type EHR. They are robust to mode collapse and scale to high-dimensional settings, often yielding the most realistic samples. The costs are substantial compute due to iterative denoising, limited interpretability, and privacy that is again mostly empirical unless paired with DP. Diffusion models are preferred when maximum realism and diversity justify substantial compute investment.

#### Autoregressive and sequence modeling frameworks

Sequence models naturally preserve temporal coherence for E and C-IR data, representing visit timing and long-range dependencies explicitly and supporting precise conditioning. Challenges arise with long-sequence cost and drift, careful treatment of irregular intervals. These models are best when faithful patient trajectories, survival dynamics, or treatment-sequence phenotypes are the goal and interpretability can be layered through attention or modular structure.

#### Statistical/Bayesian and classical time-series

When interpretability, data efficiency, and transparent assumptions are first-order requirements, statistical and Bayesian frameworks remain compelling, especially for C-IR settings with explicit hazard or transition structure. They enable explainable causal or graphical representations and support formal inferential utility. Their key limitation is restricted expressivity in high-dimensional or multimodal EHRs.

#### Simulation-informed and hybrid models

These models embed clinical priors – such as physiological constraints, care pathways, or semantic rules – and therefore achieve superior plausibility and controllability. They are ideal for E and C-IR mixtures in safety-critical contexts like CDS prototyping or digital twins. The trade-off is engineering complexity and a risk of over-constraining diversity if rule systems dominate. Use them when trust, auditability, and domain alignment outweigh maximal fidelity.

#### Cross-cutting trade-offs

High-fidelity model families (diffusion, GANs) typically depend on empirical privacy validation; formal DP integration remains rare and may degrade utility without tuning. Handling of irregular timing favors autoregressive and statistical frameworks, while dense continuous signals favor GANs and diffusion. Realism vs. controllability represents the central tension: deep black-box models yield realism; statistical or hybrid designs afford semantic control and auditability. Computational demands peak for diffusion and large Transformers; AEs and statistical methods remain efficient baselines. Current evaluation practices overweight predictive metrics, while inferential validity and clinician-rated realism remain underused.

**Practical selection (single-pass guidance)**

- For C-R waveforms with maximal fidelity goals, start with diffusion or conditional GAN/AE if compute is constrained.
- For C-IR longitudinal data requiring coherent trajectories, adopt time-aware autoregressive or hybrid models; prefer Bayesian or hybrid designs when interpretability dominates.
- For event-based EHR emphasizing controllable code-sequence realism, use hierarchical autoregressive/transformer models; for fast augmentation, conditional GAN/AE suffice.
- When privacy is primary, apply DP-aware training with accounting or complement empirical checks with design-time and post-hoc safeguards.
- When clinical realism is paramount, combine statistical or autoregressive models with expert-driven validation.

**Common failure modes and remedies**

- GANs: mitigate mode collapse with spectral or feature regularization, balanced conditioning, and diversity-aware early stopping.
- GANs/AEs: reduce inter-visit incoherence via hierarchical or segment conditioning and sequence-aware decoders.
- Autoregressive/Transformers: limit long-sequence drift with relative or continuous-time embeddings, scheduled sampling, and chunked decoding.
- Irregular intervals: use time-gap embeddings, neural ODEs, hazard-based decoders, or time-aware evaluation splits.
- AEs: counter over-smoothing with richer decoders, mixture priors, or adversarial/diffusion hybrids.

### Utility and privacy metrics (RQ3)

Evaluation taxonomies for synthetic data vary in emphasis. Some foreground fidelity or realism together with privacy, while others adopt a two-axis framework in which utility and privacy are the primary dimensions, with fidelity nested under utility. We adopt the latter, which is now widely used in healthcare SDG and consistent with recent syntheses (Kaabachi et al. [12]). This orientation also aligns task-level metrics with established practices in EHR benchmarking (Yan et al. [112]).

A potential crosswalk illustrates how existing categories map onto these dimensions. Fidelity or realism corresponds to descriptive similarity and clinical realism. Measures of distributional similarity – such as Jensen-Shannon divergence, Kolmogorov-Smirnov tests, or Wasserstein distances – fall under descriptive utility. Task performance indicators, including AUROC, F1, and calibration, are situated within predictive utility. Causal or associational validity, captured through effect sizes and confidence intervals, supports inferential validity. Privacy claims grounded in DP correspond to formal guarantees. Privacy “risk tests” are classified as empirical attacks. Finally, heuristic controls, such as rarity filters or capping, operate as design-based safeguards. Although this mapping provides a structured way of aligning different evaluation traditions, deeper comparisons remain dependent on specific use cases and application contexts.

In the case of temporal health data, including time series, longitudinal, and event-based formats, utility is typically assessed across five complementary domains: (1) qualitative evaluations, such as expert reviews; (2) descriptive statistics, which summarize distributions and trends; (3) inferential statistics, used to assess generalizability or causal inference; (4) predictive performance, gauging how well synthetic data support model training; and (5) clinical realism, which considers how faithfully synthetic data reflect real-world medical patterns. Striking a balance between utility and privacy is crucial: overly sanitized data may ensure privacy but lack analytic value, while overly realistic data can raise disclosure risks [113].

### Utility metrics

Across the literature, utility assessment is widespread but uneven. All reviewed studies reported at least one utility metric, underscoring its central role in validating synthetic data.

Descriptive statistics appeared in 96% of studies, reflecting their accessibility and foundational role. Predictive evaluations were used in 86% of studies, indicating strong interest in downstream task performance. Qualitative evaluations were used in 63% of studies. Clinical realism was assessed in 56%, pointing to growing attention to domain-specific plausibility. However, inferential utility – critical for evaluating generalizability or causal validity – was included in only 30% of studies, highlighting a significant gap. While the field clearly prioritizes utility, the absence of standardized benchmarks and the variation in methodological depth remain key challenges for advancing rigorous and comparable evaluation practices.

#### Qualitative evaluations

These evaluations focus on visual and expert-based assessments of the realism, coherence, and clinical plausibility of synthetic data. Researchers commonly present side-by-side comparisons of synthetic and real physiological signals (e.g., ECG, EEG, glucose levels), inspecting waveform morphology, temporal continuity, and variability across and within subjects (e.g [56, 105, 114–116]). Dimensionality reduction methods such as t-SNE, PCA, or UMAP are used to visualize clustering, separability, and overlap in the latent space of real and synthetic data (e.g [80, 100, 116–118]).

#### Descriptive statistics

Descriptive evaluations measure how closely synthetic data reproduce the statistical properties of real datasets. Standard summary metrics include means, medians, standard deviations, percentiles, and range checks for key clinical variables like vital signs, lab values, or biomarker levels. These approaches are used in studies such as [40, 41, 102, 119, 120]. More advanced metrics include divergence measures such as Jensen-Shannon divergence, Wasserstein distance, and K-S statistics, as applied in e.g [41]. Visual comparison tools (e.g., violin plots, histograms, and KDE curves) are used to highlight distributional similarities across these studies. Correlation matrices and autocorrelation functions assess whether synthetic data preserve both cross-sectional and temporal interdependencies, demonstrated for example by [120]. Domain-specific indicators – such as disease prevalence, frequency of visits, number of diagnosis codes, and age-of-onset – provide additional checks on whether synthetic data reflect real-world clinical heterogeneity.

#### Inferential statistics

Inferential methods assess whether synthetic data retain statistical relationships found in real data, making them suitable for hypothesis testing and causal inference. Common strategies include regression-based comparisons using logistic regression, linear regression, and Cox proportional hazards models, with evaluation of effect sizes, confidence intervals, and statistical significance, as seen in [41, 120, 121]. Likelihood-based metrics (e.g., AIC, BIC, log-likelihood) are used to evaluate model fit, such as in [39]. Group comparison tests–including K-S tests as used in [102]–examine differences in model outputs or sample distributions. K-S tests and chi-square tests are applied across several studies (e.g [53, 101, 102, 122]). Some studies, such as [47, 99, 120], also incorporate Bayesian inference or credible interval analysis to ensure that uncertainty is preserved and interpretable in synthetic datasets. Despite their importance, inferential methods remain under-used, with only 29 of the reviewed studies (e.g [44, 48, 67, 123]), applying statistical tests to evaluate whether synthetic data preserve relationships observed in real data.

#### Predictive performance

Across studies, a diverse array of prediction algorithms is used. These include tree-based models (e.g., Random Forests as in [61]), linear models (e.g., Logistic Regression in [119]), and deep learning architectures such as CNNs [102], LSTMs [92], and other neural networks like DeepSleepNet [61] and ResNet18 [39]. For time-to-event outcomes, survival models such as the Cox proportional hazards model and DeepSurv [124] are also employed, though less frequently in this subset. Some approaches incorporate multitask, autoregressive, or sequence-to-sequence models with attention mechanisms ([119], among others). Evaluation typically relies on metrics like accuracy, F1-score, precision, recall, AUROC, and AUPRC – used throughout [39, 61, 92, 102, 119]. Some studies also include calibration plots, confusion matrices, or learning curves to assess model quality. Robustness is further evaluated through subgroup analyses, variation of hyperparameters, or sensitivity to random seed initialization, offering deeper insight into model reliability.

#### Clinical realism assessments

Clinical realism assesses whether synthetic data preserve the logical and semantic coherence necessary for medical decision-making. This often involves expert evaluation of synthetic patient timelines – such as diagnoses, treatments, and outcomes – to judge whether event sequences are clinically plausible, as seen in [41, 84, 102, 121, 125]. Visual tools like Kaplan-Meier survival curves, graph-based representations, and network diagrams are used to compare treatment transitions or disease progression patterns between synthetic and real cohorts (e.g [41, 125]). Sequence alignment plots and token frequency histograms help verify that diagnostic and procedural codes occur in realistic combinations and temporal orders, as demonstrated in [102, 121]. Some studies, such as [84], employ structured rating systems or clinician-blinded comparisons to enhance objectivity. Encouragingly, plausibility checks are becoming more common, with 56% of studies now including structured assessments of clinical realism.

**Fig. 4:**
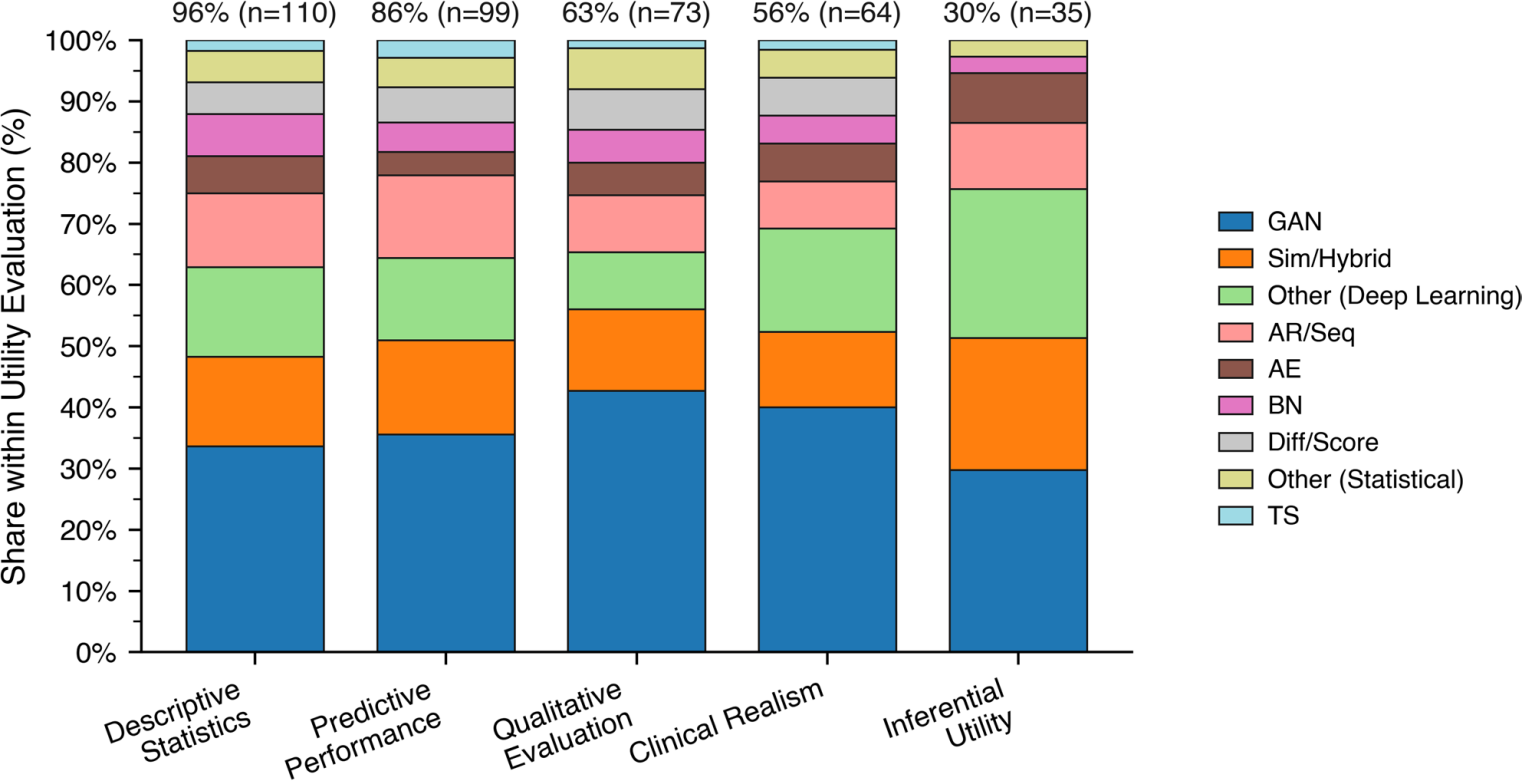
Distribution of synthesis techniques within utility categories (stacked bars). Segment heights show the within-category share of techniques. Labels above bars show each category’s share of all papers and the corresponding counts

Figure 4 shows five utility categories as stacked bars. Across the five utility domains, GANs constitute the largest single class, most prominent in descriptive and predictive evaluations, where statistical similarity and model performance are central. Their relative share decreases toward qualitative, clinical-realism, and inferential assessments, which exhibit a more heterogeneous composition. Simulation-informed and hybrid models become more visible in these downstream domains, reflecting a preference for approaches that incorporate domain knowledge or mechanistic constraints. Other deep-learning architectures, autoregressive and sequence models, autoencoders, Bayesian networks, diffusion and score-based methods, and time-series-specific techniques contribute smaller yet consistent fractions. Taken together, the pattern indicates a gradual shift from a predominantly GAN-driven landscape in standard evaluations toward a more diversified methodological ecology in context-rich and inference-oriented settings, suggesting that synthesis technique selection increasingly aligns with the evaluative focus rather than any single dominant paradigm.

### Privacy metrics

In the context of SDG for healthcare, privacy remains a critical yet inconsistently addressed concern, especially for temporally structured datasets. Although synthetic data is widely promoted as a privacy-preserving alternative to real patient records, our review reveals a substantial gap between this claim and actual implementation. Across studies, privacy is often treated as an implicit benefit rather than a rigorously evaluated property. This weakens one of the central justifications for synthetic data – its potential to enable open, compliant, and low-risk data sharing in sensitive clinical settings.

Formal privacy guarantees, most notably DP [126], are rarely implemented. Despite its status as the gold standard for mathematically provable privacy protection, DP is only referenced in a small fraction of studies around 6% [21, 23, 99, 114, 127–129]). Even among these, its application is often superficial. Crucially, not all these studies reported essential parameters such as the privacy budget (ε) or failure probability (δ) [126]. Without these values, it is impossible to assess the strength or credibility of the protection being claimed. This underscores a broader methodological gap between theoretical awareness and actual practice in SDG research. Empirical privacy evaluations are somewhat more common but remain highly inconsistent in both their use and rigor. Only 22% of studies (e.g [21, 62, 68, 80, 86, 130]), reported any form of privacy metric. Design-based safeguards were only found in 2% of the studies. Some of these used methods such as membership inference attacks [131], attribute inference, or distance-based similarity metrics [132] to test whether synthetic records can be linked back to real individuals. When applied systematically, as in [23], these tests can yield valuable insights. However, in many cases, privacy testing is mentioned only briefly or used without clear reporting of methodology or results. 70% of the studies do not include any kind of privacy evaluation approach.

This inconsistency reflects a pervasive but misleading assumption: that synthetic data is private by default. While this assumption may hold for simple or low-dimensional tabular data, it is a dangerous oversimplification when applied to high-dimensional temporal datasets. Longitudinal health records, continuous biosignals, and event-based timelines often contain structured, correlated sequences that can uniquely characterize individuals. These risks are especially acute in small or demographically narrow populations, where distinctive patterns can facilitate re-identification even without direct identifiers. Despite these vulnerabilities, very few studies explicitly address the privacy risks specific to temporal data or propose targeted mitigation strategies.

Finally, regulatory and ethical considerations are almost entirely absent from current SDG practice. Few papers reference legal frameworks such as the General Data Protection Regulation (GDPR) or the Health Insurance Portability and Accountability Act (HIPAA), and even fewer engage with national or institutional data governance policies. Ethical review, consent structures, and downstream accountability mechanisms are rarely discussed, even when synthetic data are intended for deployment in clinical or commercial settings. This omission points to a broader disconnect between technical development and the regulatory environments in which healthcare data are governed. Taken together, the fact that only 30% of studies report any privacy metric and just around 6% use DP – with almost none reporting key parameters – signals a fundamental underdevelopment in how privacy is conceptualized, implemented, and validated in synthetic temporal health data research. Without substantial methodological and regulatory alignment, the privacy promise of synthetic data remains more aspirational than assured.

**Fig. 5:**
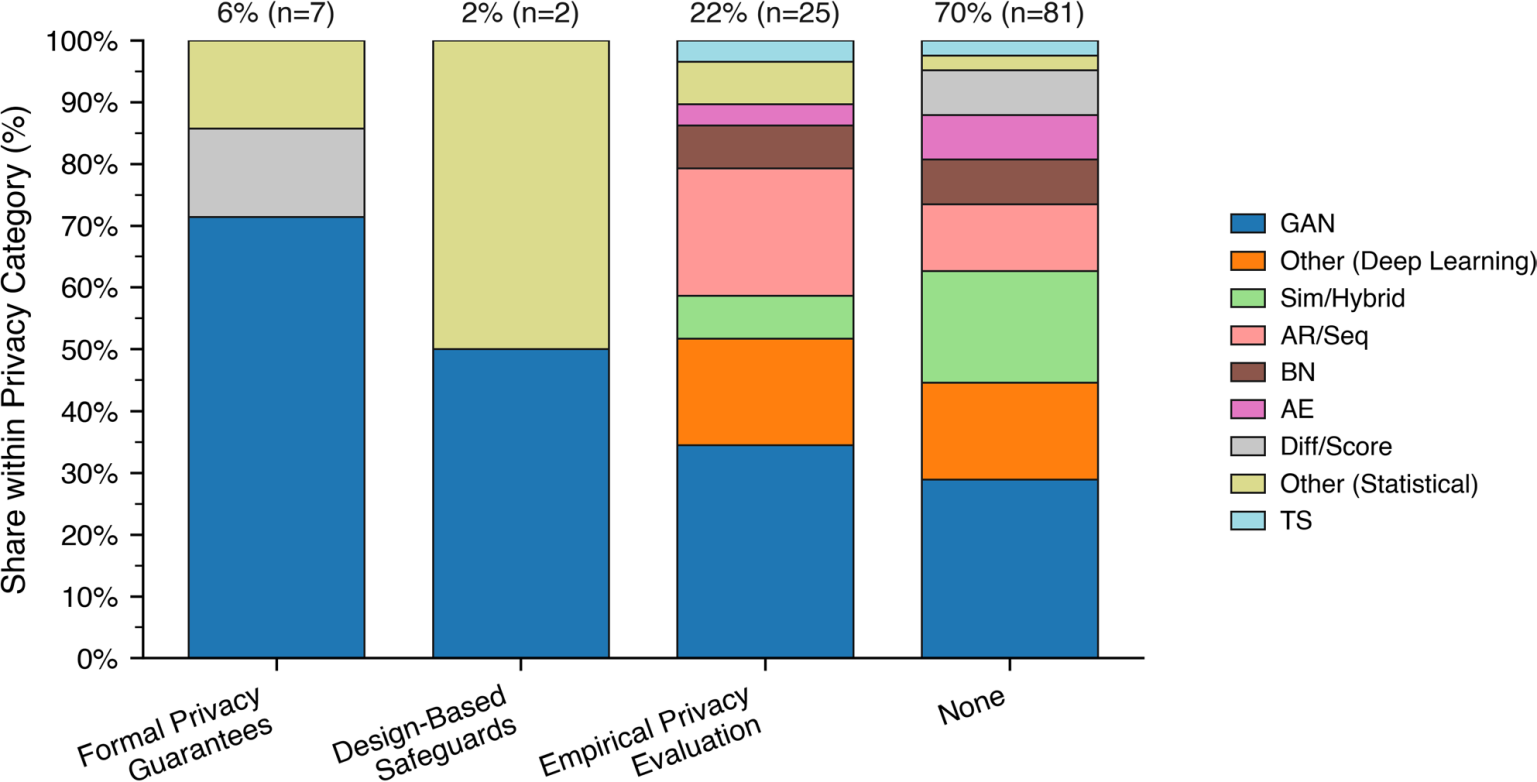
Distribution of synthesis techniques within privacy categories (stacked bars). Segment heights show the within-category share of techniques. Labels above bars show each category’s share of all papers and the corresponding counts

Figure 5 shows four privacy categories as stacked bars. GAN-based approaches dominate under formal privacy guarantees and remain prominent wherever privacy is explicitly assessed, yet their share declines as evaluations become less formalized. Categories emphasizing design-based safeguards and empirical privacy testing exhibit a more balanced mix, with rising contributions from simulation-informed, hybrid, and other deep-learning architectures, and small but consistent roles for autoregressive, Bayesian, and autoencoding models. In contrast, studies lacking privacy evaluation altogether display the broadest methodological spread, with no single family exceeding a modest plurality, indicating that privacy-agnostic work draws on a wider repertoire of synthesis paradigms. Overall, the pattern suggests a gradient of methodological concentration: the stronger the formalization of privacy, the more the field converges on GANs; the weaker or absent the privacy framing, the more diverse the methodological landscape becomes.

## Discussion

### Principal results

This review reveals the considerable growth and diversification of SDG methods targeting temporal health data, reflecting both technical innovation and increasing demand in healthcare AI. A key finding is the dominance of event-based modeling approaches, particularly for structured EHR data, with deep generative methods such as GANs and AEs leading the field. More recently, diffusion models and autoregressive transformers have gained traction, particularly for complex, irregular, or high-frequency temporal data. While event-based data remains the most widely modeled format, there is increasing attention to continuous-irregular (C-IR) data from wearables or episodic recordings, suggesting a broadening of methodological scope. Still, continuous-regular (C-R) data, common in ICU signals and biosensor outputs, are underrepresented, indicating an opportunity for greater innovation in high-resolution signal modeling.

Synthetic temporal data are applied across diverse data modalities, including physiological signals, medical imaging, video-based assessments, structured EHRs, and longitudinal trial or cohort datasets. Each modality imposes distinct requirements on fidelity, temporal coherence, and interpretability. Yet, real-world deployment remains limited. Key translational challenges include insufficient model transparency, limited awareness of regulatory frameworks (e.g., GDPR, HIPAA), and lack of clinical alignment. Many models fail to integrate domain knowledge or to incorporate feedback loops with clinicians. Without these elements, synthetic data may fall short of the operational, ethical, or epistemic demands of clinical environments. Modality-specific realism checks, rigorous interpretability protocols, and co-design processes with clinical stakeholders are critical to bridging this translational gap.

Evaluation of SDG methods for temporal health data remains uneven and fragmented. On the utility side, studies employ a diverse range of metrics–ranging from descriptive statistics and predictive modeling to inferential testing and expert-based realism checks. While this diversity reflects the variety of use cases, it also leads to inconsistencies that undermine comparability and reproducibility. Common predictive metrics such as AUROC or F1-score are applied inconsistently, and clinical realism evaluations often lack structured or reproducible protocols.

Privacy evaluations are even less developed. Most studies provide narrative assurances or informal similarity analyses rather than formal guarantees. DP, though referenced in several papers, is rarely implemented. Key factors likely to contribute are the limited adoption of DP: (i) the technical difficulty of applying DP to high-dimensional, temporally correlated health data (privacy loss composes over long sequences and often requires heavy noise); (ii) limited off-the-shelf tooling and expertise (e.g., DP-SGD, privacy accounting, hyperparameter tuning); (iii)–inconsistent reporting standards (missing ε/δ or accountant assumptions), which hampers review and replication; and (iv) weak external incentives – venues and regulators commonly encourage de-identification but rarely require DP for synthetic data. Specifically, HIPAA [133] and GDPR [134] emphasize governance but do not mandate technical guarantees. Consequently, many studies prefer heuristic disclosure controls over formal DP.

Sparse and inconsistent privacy reporting reflects (i) structural constraints (Institutional Review Board (IRB) or Data-usage-Agreement (DUA) limits, novelty-biased incentives, and reluctance to publish negative results); (ii) methodological ambiguity (different risks – identity, linkage, membership, attribute inference – depend on attacker knowledge and auxiliary data, yet threat models are rarely specified); (iii) practical barriers (compute cost, fragile implementations, and lack of real holdouts, especially for temporal clinical data); and (iv) a tooling/skills gap (few reusable attack harnesses). One remedy would be a minimum privacy reporting checklist (Box 1). We recommend evaluating on at least one open or DUA-gated benchmark, releasing a lightweight, reusable privacy attack harness (membership, linkage, uniqueness, attribute inference), and reporting confidence intervals, baselines, and seeds/configs to enable red-teaming (to run adversarial tests) and comparability.

**Box 1:** Proposed minimum privacy reporting checklist (for authors)

1. **Threat model**. Specify the targeted risk (identity, linkage, membership, or attribute inference), assumed attacker knowledge and auxiliary data, success criterion, and acceptable risk threshold.
2. **Attack specifications**. Provide full details of privacy attacks: algorithms, hyperparameters, seeds, runtime or compute budget, and attacker auxiliary data sources.
3. **Data and access**. Describe data provenance, cohort sizes, and whether a real holdout was used; note IRB or DUA constraints.
4. **Splits and leakage control**. Detail train/validation/test split rules, including time-aware schemes for longitudinal data; confirm deduplication and document any leakage detection methods.
5. **Utility and privacy metrics** (with confidence intervals). Report both aspects jointly, including sensitivity analyses for robustness.
6. **Baselines**. Compare against raw data (if permissible) or a realistic proxy, simple perturbation, and a DP baseline when DP is claimed; justify any omissions.
7. **DP accounting** (if applicable). Report ε, δ, composition or accounting method, clipping and noise parameters, and sensitivity settings.
8. **Reproducibility artifacts**. Share code and configurations, plus a small synthetic sample or red-teamable sandbox, and document known failure modes or limitations.

### Limitations

While comprehensive in scope, this review as certain limitations. First, it focuses exclusively on SDG for temporal healthcare data, thereby excluding potentially relevant advances in SDG for static, non-temporal, or non-healthcare datasets that may offer transferable insights. Second, our literature search includes publications only up to April 2025 and may therefore miss emerging contributions that reflect the most recent innovations in transformer-based synthesis, privacy frameworks, or domain-specific benchmarks. Third, methodological heterogeneity across studies, particularly in reporting standards and evaluation metrics, limits the potential for meta-analytic synthesis and makes cross-study comparison challenging.

With the comparative-synthesis subsection, we go beyond descriptive mapping and summarize cross-family patterns, trade-offs, and failure modes. However, deeper comparisons must be use-case-specific, not only general. Method suitability depends on the temporal data type, data modality, clinical constraints (e.g., plausibility, auditability), privacy goals (e.g., DP budgets and threat models), and operational factors (compute, latency, integration). Robust head-to-head claims would require task-linked benchmarks with harmonized pipelines, shared datasets and time-aware splits, standardized utility-privacy protocols, and governance assumptions held constant. Accordingly, we stop at a qualitative synthesis and a pragmatic selection heuristic; ranking methods across domains is out of scope and is best addressed by dedicated, use-case–driven benchmark studies.

Finally, this review highlights the limited and inconsistent treatment of privacy across the literature but does not itself propose a standardized remedy. Sparse reporting, lack of baselines, and incomplete disclosure of parameters constrain the comparability of findings, and our review cannot fully resolve these shortcomings. While we point to potential remedies – including the adoption of minimum reporting standards, shared benchmark tasks, and open release of evaluation code – these recommendations remain aspirational until embraced by the broader community. Ethical oversight, legal frameworks, and journal policies will also play an important role in shaping transparent and accountable evaluation practices.

### Future directions

Looking ahead, several promising research avenues emerge. First, more attention should be devoted to the development of SDG techniques for hybrid data structures that combine discrete clinical events with continuous biosignal streams, especially in settings like mobile health, remote monitoring, and ICU telemetry. These mixed data types present unique modeling challenges due to differences in sampling frequency, semantic granularity, and missingness patterns.

Second, while some work has begun to assess both privacy and utility in synthetic temporal data, there is still no standard framework for doing so. Privacy guarantees like DP or membership inference resistance are often evaluated separately from utility measures, making it difficult to compare methods or understand trade-offs. To enable meaningful, reproducible evaluation, privacy and utility must be assessed in an integrated manner, especially in time-sensitive clinical settings where both matter equally.

Third, the field needs high-quality, publicly available temporal datasets to serve as standardized benchmarks. As our typology shows, current research frequently references a handful of prominent real-world datasets – such as MIT-BIH, PTB-XL, and PhysioNet (C-R); TREND and PPMI (C-IR); or MIMIC-III/IV, OMOP, and UK Biobank (E) – yet these are still not widely used across studies. To enable reproducibility and meaningful cross-comparison, benchmark datasets should be clinically grounded, temporally structured, and released with clear documentation, baseline performance metrics, and well-defined domain-specific tasks. In the absence of such standardized resources, methodological progress remains difficult to compare or generalize.

Fourth, further theoretical and ontological development is needed to clarify foundational concepts such as “event”, “trajectory”, and “temporal abstraction”. Advancing the semantic and formal grounding of these terms will improve the interoperability of SDG methods with clinical knowledge systems, medical ontologies, and decision-support logic.

Fifth, as SDG is largely AI-driven, responsible AI and data governance are essential, covering three pillars – fairness, transparency, and trust. Fairness means reporting subgroup performance to prevent bias. Transparency requires documenting data provenance and consent, preprocessing, assumptions, and evaluation with versioning. Trust involves stating uncertainty and failure modes and ensuring external validation. Black-box models increase the need for explainability, reproducibility, and auditability, while the EU AI Act [135] and FDA AI/ML guidance [136] raise expectations for risk class, traceability, change control, and post-market monitoring. Practical steps include SDG-specific reporting checklists, model/data cards with code and versioned pipelines, and interdisciplinary oversight across the lifecycle.

Finally, interdisciplinary collaboration should be actively fostered. Bringing together clinicians, informaticians, privacy experts, and machine learning researchers is essential to ensure that synthetic data methods are not only technically robust but also clinically meaningful, ethically grounded, and implementation ready. Frameworks that embed stakeholder input at all stages – from problem formulation to evaluation and deployment – will play a key role in making synthetic data a practical and trustworthy tool in real-world healthcare.

## Conclusion

This review makes a dual contribution: it offers a methodological synthesis of SDG techniques for temporal healthcare data and introduces a structured taxonomy that clarifies conceptual and practical distinctions. The potential of synthetic data in healthcare is vast, but it will only be realized through rigorous modeling, transparent evaluation, and application-sensitive design. By laying conceptual and methodological groundwork, this review supports the development of shared standards, interdisciplinary collaboration, and ethical deployment in real-world contexts.

## Supplementary Information

Below is the link to the electronic supplementary material.

Supplementary Material 1

Supplementary Material 2

## Data Availability

All data generated or analysed during this study are included in this published article and its supplementary information files.
