# Supplementary material for: Synthetic data generation methods for longitudinal and time series health data: a systematic review
Source: BMC Med Inform Decis Mak. 2025 Dec 24;26:30. doi: 10.1186/s12911-025-03326-8 (PMC12849454; doi:10.1186/s12911-025-03326-8)
Supplement: Supplementary file 1 — Supplementary Material 1 [file 12911_2025_3326_MOESM1_ESM.docx]

Table S1: Overview of studies from the systematic literature review mapped to key evaluation criteria. Each study is classified according to key aspects of our review framework: temporal data type, synthesis technique, utility evaluation methods, privacy assessment category, and data modality. Privacy category reflects the strongest evidence reported (Formal > Empirical > Design-based > None). ‘None’ = no technical mechanism and no empirical privacy test; governance/de-identification statements alone were not counted. Studies labeled ‘None’ met inclusion via utility evaluation.

| **Authors** | **Temporal Data Type** | **Synthesis Technique** | **Utility Evaluation** | **Privacy Category^[[1]](#footnote-1)^** | **Data Modality** |
| --- | --- | --- | --- | --- | --- |
| You et al. (1) | C-R, E | GAN | Clinical Realism, Descriptive Statistics, Predictive Performance, Qualitative Evaluation | None | Physiological Signals |
| Deltadahl et al. (2) | C-IR | AE | Clinical Realism, Descriptive Statistics, Inferential Utility, Predictive Performance | None | Medical Imaging and Video |
| Darvish and Kist (3) | C-R | Bayesian Networks | Clinical Realism, Descriptive Statistics, Qualitative Evaluation | None | Medical Imaging and Video |
| Mosquera et al. (4) | E | Autoregressive and Sequence Modeling Frameworks | Descriptive Statistics, Inferential Utility, Qualitative Evaluation | Empirical Privacy Evaluation | Electronic Health Records (EHRs) |
| Cao et al.(5) | C-R | Autoregressive and Sequence Modeling Frameworks | Descriptive Statistics, Inferential Utility, Predictive Performance | None | Physiological Signals |
| Boukhennoufa et al. (6) | C-R, C-IR | Autoregressive and Sequence Modeling Frameworks | Clinical Realism, Descriptive Statistics, Predictive Performance, Qualitative Evaluation | None | Physiological Signals |
| Wang et al. (7) | C-R | GAN | Clinical Realism, Descriptive Statistics, Inferential Utility, Predictive Performance, Qualitative Evaluation | None | Physiological Signals, Medical Imaging and Video |
| Asadi et al. (8) | C-R | Simulation-Informed and Hybrid Models | Clinical Realism, Descriptive Statistics, Inferential Utility, Predictive Performance, Qualitative Evaluation | None | Medical Imaging and Video |
| Li et al. (9) | C-R | Simulation-Informed and Hybrid Models | Descriptive Statistics, Predictive Performance, Qualitative Evaluation | None | Physiological Signals |
| Hao et al. (10) | C-R | GAN | Clinical Realism, Descriptive Statistics, Predictive Performance, Qualitative Evaluation | None | Physiological Signals |
| Kuo et al. (11) | E | GAN | Descriptive Statistics, Inferential Utility, Qualitative Evaluation | None | Clinical Trials and Cohort Studies |
| Nikolaidis et al. (12) | C-R | GAN | Descriptive Statistics, Predictive Performance, Qualitative Evaluation | None | Physiological Signals |
| Sood et al. (13) | C-IR, E | GAN | Descriptive Statistics, Inferential Utility, Predictive Performance, Qualitative Evaluation | None | Clinical Trials and Cohort Studies |
| Che et al. (14) | E | GAN | Descriptive Statistics, Inferential Utility, Predictive Performance, Qualitative Evaluation | None | Clinical Trials and Cohort Studies |
| Li et al. (15) | C-R | Simulation-Informed and Hybrid Models | Descriptive Statistics, Inferential Utility, Predictive Performance, Qualitative Evaluation | None | Physiological Signals, Medical Imaging and Video |
| Pang et al. (16) | E | Autoregressive and Sequence Modeling Frameworks | Descriptive Statistics, Predictive Performance | Empirical Privacy Evaluation | Electronic Health Records (EHRs) |
| Sun et al. (17) | E | Autoregressive and Sequence Modeling Frameworks | Descriptive Statistics, Predictive Performance | Empirical Privacy Evaluation | Electronic Health Records (EHRs) |
| Bing et al. (18) | E | AE | Descriptive Statistics, Predictive Performance | Empirical Privacy Evaluation | Electronic Health Records (EHRs) |
| Theodorou et al. (19) | E | Autoregressive and Sequence Modeling Frameworks | Clinical Realism, Descriptive Statistics, Predictive Performance | None | Electronic Health Records (EHRs) |
| Wang et al. (20) | C-R | GAN | Clinical Realism, Descriptive Statistics, Inferential Utility, Predictive Performance, Qualitative Evaluation | None | Electronic Health Records (EHRs) |
| Liu et al. (21) | C-R, E | Autoregressive and Sequence Modeling Frameworks | Descriptive Statistics, Inferential Utility, Predictive Performance | None | Physiological Signals, Electronic Health Records (EHRs) |
| Kallidromitis et al. (22) | C-R | Autoregressive and Sequence Modeling Frameworks | Descriptive Statistics, Inferential Utility, Predictive Performance | None | Physiological Signals, Medical Imaging and Video, Clinical Trials and Cohort Studies |
| Torfi and Fox (23) | C-R, C-IR, E | GAN | Clinical Realism, Descriptive Statistics, Inferential Utility, Predictive Performance, Qualitative Evaluation | Empirical Privacy Evaluation | Electronic Health Records (EHRs) |
| Choi and Kim (24) | C-IR | Autoencoder | Predictive Performance | None | Clinical Trials and Cohort Studies |
| Haleem et al. (25) | C-R, C-IR, E | Simulation-Informed and Hybrid Models | Clinical Realism, Descriptive Statistics, Inferential Utility, Predictive Performance | None | Medical Imaging and Video |
| Kim et al. (26) | C-IR, E | GAN | Clinical Realism, Descriptive Statistics, Predictive Performance, Qualitative Evaluation | None | Electronic Health Records (EHRs) |
| Torfi et al. (27) | C-R, C-IR, E | GAN | Descriptive Statistics, Predictive Performance | Formal Privacy Guarantees | Physiological Signals, Medical Imaging and Video, Electronic Health Records (EHRs) |
| Alcatraz and Strodthoff (28) | C-R | Diffusion and Score-Based Methods | Clinical Realism, Descriptive Statistics, Predictive Performance, Qualitative Evaluation | None | Physiological Signals |
| Kaisti et al. (29) | C-R | Simulation-Informed and Hybrid Models | Clinical Realism, Descriptive Statistics, Predictive Performance, Qualitative Evaluation | None | Physiological Signals |
| Habiba et al. (30) | C-R | GAN | Clinical Realism, Descriptive Statistics, Predictive Performance, Qualitative Evaluation | None | Physiological Signals |
| Yoon et al. (31) | E | GAN | Clinical Realism, Descriptive Statistics, Inferential Utility, Predictive Performance, Qualitative Evaluation | Empirical Privacy Evaluation | Electronic Health Records (EHRs) |
| Alawneh et al. (32) | C-R | Autoregressive and Sequence Modeling Frameworks | Descriptive Statistics, Inferential Utility, Predictive Performance | None | Physiological Signals |
| Biswal et al. (33) | E | AE | Clinical Realism, Descriptive Statistics, Predictive Performance, Qualitative Evaluation | Empirical Privacy Evaluation | Electronic Health Records (EHRs) |
| Tarek et al. (34) | E | Autoencoder | Clinical Realism, Descriptive Statistics, Predictive Performance | None | Electronic Health Records (EHRs) |
| Eyobu and Han (35) | C-IR | Autoencoder | Descriptive Statistics, Predictive Performance | None | Electronic Health Records (EHRs) |
| Dissanayake et al. (36) | C-R, E | GAN | Clinical Realism, Descriptive Statistics, Predictive Performance, Qualitative Evaluation | None | Physiological Signals |
| Zhou and Barbieri (37) | C-R | Simulation-Informed and Hybrid Models | Clinical Realism, Descriptive Statistics, Predictive Performance, Qualitative Evaluation | Empirical Privacy Evaluation | Electronic Health Records (EHRs) |
| Wulan et al. (38) | C-R | GAN | Clinical Realism, Descriptive Statistics, Predictive Performance, Qualitative Evaluation | None | Physiological Signals |
| Shi et al. (39) | E | GAN | Descriptive Statistics, Inferential Utility, Predictive Performance, Qualitative Evaluation | Empirical Privacy Evaluation | Electronic Health Records (EHRs) |
| Seyfi et al. (40) | C-R | GAN | Clinical Realism, Descriptive Statistics, Predictive Performance, Qualitative Evaluation | None | Physiological Signals |
| Hoorn et al. (41) | C-R, C-IR | GAN | Clinical Realism, Descriptive Statistics, Predictive Performance, Qualitative Evaluation | Design-Based Safeguards | Electronic Health Records (EHRs) |
| Vetter et al. (42) | C-R | GAN | Clinical Realism, Descriptive Statistics, Predictive Performance, Qualitative Evaluation | None | Physiological Signals |
| Lee et al. (43) | E | Simulation-Informed and Hybrid Models | Clinical Realism, Descriptive Statistics, Predictive Performance, Qualitative Evaluation | Formal Privacy Guarantees | Electronic Health Records (EHRs) |
| Kuo et al. (44) | E | GAN | Clinical Realism, Descriptive Statistics, Inferential Utility, Predictive Performance, Qualitative Evaluation | Empirical Privacy Evaluation | Physiological Signals |
| Lange et al. (45) | C-R | GAN | Clinical Realism, Descriptive Statistics, Predictive Performance, Qualitative Evaluation | Formal Privacy Guarantees | Medical Imaging and Video |
| Li et al. (46) | C-R | Simulation-Informed and Hybrid Models | Clinical Realism, Descriptive Statistics, Predictive Performance, Qualitative Evaluation | Formal Privacy Guarantees | Electronic Health Records (EHRs) |
| Wendland et al. (47) | C-IR | Autoencoder | Clinical Realism, Descriptive Statistics, Predictive Performance, Qualitative Evaluation | None | Clinical Trials and Cohort Studies |
| Gall et al. (48) | C-R | Simulation-Informed and Hybrid Models | Clinical Realism, Descriptive Statistics | Empirical Privacy Evaluation | Physiological Signals |
| Linial et al. (49) | C-R | Simulation-Informed and Hybrid Models | Descriptive Statistics, Predictive Performance | None | Medical Imaging and Video |
| Zhu et al. (50) | C-R | GAN | Descriptive Statistics, Predictive Performance, Qualitative Evaluation | None | Physiological Signals |
| Brouwer et al. (51) | C-IR, E | Simulation-Informed and Hybrid Models | Descriptive Statistics, Predictive Performance | None | Electronic Health Records (EHRs) |
| Ghosheh et al. (52) | C-R, C-IR | Simulation-Informed and Hybrid Models | Clinical Realism, Descriptive Statistics, Inferential Utility, Predictive Performance | None | Electronic Health Records (EHRs) |
| Lee et al. (53) | E | Simulation-Informed and Hybrid Models | Descriptive Statistics, Inferential Utility, Predictive Performance, Qualitative Evaluation | None | Physiological Signals |
| Fallahzadeh et al. (54) | E | GAN | Clinical Realism, Descriptive Statistics, Predictive Performance, Qualitative Evaluation | None | Electronic Health Records (EHRs) |
| Alt et al. (55) | C-IR | Bayesian Networks, Time-series (ARIMA, ‚Ä¶) | Descriptive Statistics, Inferential Utility | None | Clinical Trials and Cohort Studies |
| Zhang et al. (56) | E | Simulation-Informed and Hybrid Models | Descriptive Statistics, Predictive Performance | Empirical Privacy Evaluation | Electronic Health Records (EHRs) |
| Ramchandran et al. (57) | C-R, C-IR | AE | Descriptive Statistics, Predictive Performance, Qualitative Evaluation | None | Clinical Trials and Cohort Studies |
| Schulz et al. (58) | E | Bayesian Networks | Clinical Realism, Descriptive Statistics, Inferential Utility, Qualitative Evaluation | None | Clinical Trials and Cohort Studies |
| Ramchandran et al. (59) | C-R, C-IR | AE | Descriptive Statistics | None | Electronic Health Records (EHRs) |
| Fischer et al. (60) | C-IR | Autoencoder | Descriptive Statistics | None | Clinical Trials and Cohort Studies |
| Zhong et al. (61) | C-R | Diffusion and Score-Based Models | Clinical Realism, Descriptive Statistics, Predictive Performance, Qualitative Evaluation | None | Electronic Health Records (EHRs) |
| Wickstrøm et al. (62) | C-R | Diffusion and Score-Based Methods | Predictive Performance | None | Physiological Signals |
| Poyraz and Marttinen (63) | C-IR | Autoencoder | Descriptive Statistics, Inferential Utility, Predictive Performance | None | Clinical Trials and Cohort Studies |
| Ceritli et al. (64) | C-R, C-IR | Bayesian Networks | Descriptive Statistics, Predictive Performance | None | Medical Imaging and Video, Electronic Health Records (EHRs) |
| Silva and Matos (65) | C-R | Bayesian Networks | Descriptive Statistics, Predictive Performance | None | Electronic Health Records (EHRs) |
| Lu et al. (66) | E | GAN | Clinical Realism, Descriptive Statistics, Predictive Performance, Qualitative Evaluation | None | Electronic Health Records (EHRs) |
| Moazemi et al. (67) | C-R, C-IR, E | Simulation-Informed and Hybrid Models | Descriptive Statistics, Predictive Performance | Empirical Privacy Evaluation | Medical Imaging and Video, Clinical Trials and Cohort Studies |
| Kiyasseh et al. (68) | C-R | GAN | Clinical Realism, Descriptive Statistics, Predictive Performance, Qualitative Evaluation | None | Physiological Signals |
| Li et al. (69) | C-R | Simulation-Informed and Hybrid Models | Clinical Realism, Descriptive Statistics, Predictive Performance, Qualitative Evaluation | None | Medical Imaging and Video, Electronic Health Records (EHRs) |
| Wang and Sun (70) | C-R | GAN | Descriptive Statistics, Predictive Performance, Qualitative Evaluation | Empirical Privacy Evaluation | Electronic Health Records (EHRs) |
| Ashrafi et al. (71) | C-R | GAN | Clinical Realism, Descriptive Statistics, Inferential Utility, Predictive Performance, Qualitative Evaluation | Empirical Privacy Evaluation | Medical Imaging and Video |
| Yadav et al. (72) | C-R | GAN | Descriptive Statistics, Inferential Utility, Predictive Performance, Qualitative Evaluation | None | Physiological Signals |
| Yu et al. (73) | E | GAN | Predictive Performance | None | Electronic Health Records (EHRs) |
| Hyland et al. (74) | C-R | GAN | Descriptive Statistics, Predictive Performance, Qualitative Evaluation | Formal Privacy Guarantees | Electronic Health Records (EHRs) |
| Sood et al. (75) | C-IR, E | GAN | Clinical Realism, Descriptive Statistics, Inferential Utility, Predictive Performance, Qualitative Evaluation | None | Clinical Trials and Cohort Studies |
| Tian et al. (76) | C-R, E | Diffusion and Score-Based Models | Descriptive Statistics, Predictive Performance, Qualitative Evaluation | Empirical Privacy Evaluation | Electronic Health Records (EHRs) |
| Afrin et al. (77) | C-R | Other | Clinical Realism, Descriptive Statistics, Predictive Performance, Qualitative Evaluation | None | Physiological Signals |
| Sørensen et al. (78) | C-R | Other | Clinical Realism, Descriptive Statistics, Predictive Performance, Qualitative Evaluation | None | Medical Imaging and Video |
| Hsieh et al. (79) | E | Other | Descriptive Statistics, Inferential Utility, Predictive Performance | None | Physiological Signals |
| Ming et al. (80) | C-R | Other | Clinical Realism, Descriptive Statistics, Predictive Performance, Qualitative Evaluation | None | Medical Imaging and Video |
| Norcliffe et al. (81) | C-R | GAN | Clinical Realism, Descriptive Statistics, Inferential Utility, Predictive Performance | None | Electronic Health Records (EHRs), Clinical Trials and Cohort Studies |
| Hazra and Byun (82) | C-R, E | Other | Clinical Realism, Descriptive Statistics, Qualitative Evaluation | None | Physiological Signals, Medical Imaging and Video |
| Dahmen and Cook (83) | E | Other | Clinical Realism, Descriptive Statistics, Qualitative Evaluation | None | Medical Imaging and Video |
| Zhang et al. (84) | E | Simulation-Informed and Hybrid Models | Descriptive Statistics, Inferential Utility, Predictive Performance, Qualitative Evaluation | Empirical Privacy Evaluation | Electronic Health Records (EHRs) |
| Walonoski et al. (85) | E | Other | Clinical Realism, Descriptive Statistics, Qualitative Evaluation | None | Electronic Health Records (EHRs) |
| Brophy et al. (86) | C-R | Other | Descriptive Statistics, Predictive Performance, Qualitative Evaluation | Formal Privacy Guarantees | Physiological Signals |
| Delaney et al. (87) | C-R | GAN | Descriptive Statistics, Predictive Performance, Qualitative Evaluation | Empirical Privacy Evaluation | Physiological Signals |
| Theodorou et al. (88) | E | Autoregressive and Sequence Modeling Frameworks | Clinical Realism, Descriptive Statistics, Predictive Performance | None | Electronic Health Records (EHRs) |
| Theodorou et al. (89) | E | Autoregressive and Sequence Modeling Frameworks | Clinical Realism, Descriptive Statistics, Predictive Performance | Empirical Privacy Evaluation | Electronic Health Records (EHRs) |
| Zhong et al. (90) | C-R | Diffusion and Score-Based Models | Descriptive Statistics, Predictive Performance | Empirical Privacy Evaluation | Electronic Health Records (EHRs), Clinical Trials and Cohort Studies |
| Foomani et al. (91) | C-IR | GAN | Descriptive Statistics, Predictive Performance, Qualitative Evaluation | None | Electronic Health Records (EHRs) |
| Kühnel et al. (92) | C-IR | Simulation-Informed and Hybrid Models | Clinical Realism, Descriptive Statistics, Inferential Utility, Predictive Performance, Qualitative Evaluation | None | Medical Imaging and Video |
| Nikolentzos et al. (93) | E | AE | Clinical Realism, Descriptive Statistics, Inferential Utility, Predictive Performance, Qualitative Evaluation | Empirical Privacy Evaluation | Electronic Health Records (EHRs) |
| Kulyabin et al. (94) | C-R | GAN | Clinical Realism, Descriptive Statistics, Predictive Performance, Qualitative Evaluation | None | Medical Imaging and Video |
| Dash et al. (95) | E | GAN | Clinical Realism, Descriptive Statistics, Qualitative Evaluation | None | Electronic Health Records (EHRs) |
| Kuo et al. (96) | E | GAN | Clinical Realism, Descriptive Statistics, Inferential Utility, Qualitative Evaluation | Empirical Privacy Evaluation | Electronic Health Records (EHRs) |
| Larrea et al. (97) | C-IR | Other | Clinical Realism, Descriptive Statistics, Qualitative Evaluation | None | Physiological Signals |
| Hashemi et al. (98) | E | GAN | Descriptive Statistics, Predictive Performance, Qualitative Evaluation | Design-Based Safeguards | Electronic Health Records (EHRs) |
| Dubey et al. (99) | E | Other | Descriptive Statistics, Predictive Performance | None | Electronic Health Records (EHRs) |
| Lee et al. (100) | E | Simulation-Informed and Hybrid Models | Predictive Performance | None | Medical Imaging and Video |
| Chang et al. (101) | C-R | Other | Clinical Realism, Predictive Performance | None | Physiological Signals, Medical Imaging and Video, Electronic Health Records (EHRs) |
| Qian et al. (102) | C-R | Diffusion and Score-Based Models | Clinical Realism, Descriptive Statistics, Predictive Performance, Qualitative Evaluation | None | Physiological Signals, Medical Imaging and Video |
| Song et al. (103) | E | Bayesian Networks | Descriptive Statistics, Predictive Performance | None | Electronic Health Records (EHRs) |
| Gao et al. (104) | E | Simulation-Informed and Hybrid Models | Descriptive Statistics, Predictive Performance | Empirical Privacy Evaluation | Clinical Trials and Cohort Studies |
| Yalavarthi et al. (105) | C-R, C-IR, E | Other | Descriptive Statistics, Inferential Utility, Predictive Performance | None | Medical Imaging and Video, Electronic Health Records (EHRs) |
| Yang et al. (106) | C-R, E | AE | Clinical Realism, Descriptive Statistics, Predictive Performance, Qualitative Evaluation | None | Physiological Signals |
| Li et al. (107) | C-R | Simulation-Informed and Hybrid Models | Clinical Realism, Descriptive Statistics, Predictive Performance, Qualitative Evaluation | None | Physiological Signals, Medical Imaging and Video |
| Li et al. (108) | C-R | Simulation-Informed and Hybrid Models | Clinical Realism, Descriptive Statistics, Predictive Performance, Qualitative Evaluation | None | Physiological Signals, Medical Imaging and Video |
| Wang et al. (109) | C-R | GAN | Clinical Realism, Descriptive Statistics, Predictive Performance, Qualitative Evaluation | Empirical Privacy Evaluation | Clinical Trials and Cohort Studies |
| Das et al. (110) | E | Simulation-Informed and Hybrid Models | Clinical Realism, Descriptive Statistics, Predictive Performance | Empirical Privacy Evaluation | Clinical Trials and Cohort Studies |
| Li et al. (111) | C-R | Simulation-Informed and Hybrid Models | Descriptive Statistics, Predictive Performance | None | Physiological Signals, Medical Imaging and Video, Electronic Health Records (EHRs) |
| Wang et al. (112) | C-R | GAN | Clinical Realism, Descriptive Statistics, Qualitative Evaluation | None | Electronic Health Records (EHRs) |
| Moore et al. (113) | C-R | Other | Clinical Realism, Descriptive Statistics, Inferential Utility, Predictive Performance, Qualitative Evaluation | None | Physiological Signals, Electronic Health Records (EHRs), Clinical Trials and Cohort Studies |
| Gootjes-Dreesbach et al. (114) | C-IR | Simulation-Informed and Hybrid Models | Clinical Realism, Descriptive Statistics, Inferential Utility, Qualitative Evaluation | Formal Privacy Guarantees | Clinical Trials and Cohort Studies |
| Arabi et al. (115) | C-R | Other | Descriptive Statistics, Predictive Performance | None | Electronic Health Records (EHRs) |

References

1. You Y, Guo X, Zhong X, Yang Z. A Few-Shot Learning-Based EEG and Stage Transition Sequence Generator for Improving Sleep Staging Performance. Biomedicines. 2022 Nov;10(12):3006.

2. Deltadahl S, Vall A, Ivaturi V, Korsbo N. A Framework for Evaluating Predictive Models Using Synthetic Image Covariates and Longitudinal Data [Internet]. arXiv; 2024 [cited 2025 Mar 26]. Available from: http://arxiv.org/abs/2410.16177

3. Darvish M, Kist AM. A Generative Method for a Laryngeal Biosignal. J Voice [Internet]. 2024 Feb [cited 2025 Apr 11]; Available from: https://www.sciencedirect.com/science/article/pii/S0892199724000195

4. Mosquera L, El Emam K, Ding L, Sharma V, Zhang XH, Kababji SE, et al. A method for generating synthetic longitudinal health data. BMC Med Res Methodol. 2023 Mar;23(1):67.

5. Cao P, Li X, Mao K, Lu F, Ning G, Fang L, et al. A novel data augmentation method to enhance deep neural networks for detection of atrial fibrillation. Biomed Signal Process Control. 2020 Feb;56:101675.

6. Boukhennoufa I, Jarchi D, Zhai X, Utti V, Sanei S, Lee TKM, et al. A Novel Model to Generate Heterogeneous and Realistic Time-Series Data for Post-Stroke Rehabilitation Assessment. IEEE Trans Neural Syst Rehabil Eng. 2023;31:2676–87.

7. Wang J, Chen Y, Gu Y. A wearable-HAR oriented sensory data generation method based on spatio-temporal reinforced conditional GANs. Neurocomputing. 2022 July;493:548–67.

8. Asadi M, Poursalim F, Loni M, Daneshtalab M, Sjödin M, Gharehbaghi A. Accurate detection of paroxysmal atrial fibrillation with certified-GAN and neural architecture search. Sci Rep. 2023 July;13:11378.

9. Li X, Luo J, Younes R. ActivityGAN: generative adversarial networks for data augmentation in sensor-based human activity recognition. In: Adjunct Proceedings of the 2020 ACM International Joint Conference on Pervasive and Ubiquitous Computing and Proceedings of the 2020 ACM International Symposium on Wearable Computers [Internet]. New York, NY, USA: Association for Computing Machinery; 2020 [cited 2025 Apr 17]. p. 249–54. (UbiComp/ISWC ’20 Adjunct). Available from: https://dl.acm.org/doi/10.1145/3410530.3414367

10. Hao L, Bakkes THGF, van Diepen A, Chennakeshava N, Bouwman RA, De Bie Dekker AJR, et al. An adversarial learning approach to generate pressure support ventilation waveforms for asynchrony detection. Comput Methods Programs Biomed. 2024 June;250:108175.

11. Kuo NIH, Gallego B, Jorm L. Attention-Based Synthetic Data Generation for Calibration-Enhanced Survival Analysis: A Case Study for Chronic Kidney Disease Using Electronic Health Records [Internet]. arXiv; 2025 [cited 2025 Mar 26]. Available from: http://arxiv.org/abs/2503.06096

12. Nikolaidis K, Kristiansen S, Goebel V, Plagemann T, Liestøl K, Kankanhalli M. Augmenting Physiological Time Series Data: A Case Study for Sleep Apnea Detection [Internet]. arXiv; 2019 [cited 2025 Apr 17]. Available from: http://arxiv.org/abs/1905.09068

13. Sood M, Suenkel U, Thaler AK von, Zacharias HU, Brockmann K, Eschweiler GW, et al. Bayesian network modeling of risk and prodromal markers of Parkinson’s disease. PLOS ONE. 2023 Feb;18(2):e0280609.

14. Che Z, Cheng Y, Zhai S, Sun Z, Liu Y. Boosting Deep Learning Risk Prediction with Generative Adversarial Networks for Electronic Health Records [Internet]. arXiv; 2017 [cited 2025 Mar 26]. Available from: http://arxiv.org/abs/1709.01648

15. Li H, Yu S, Principe J. Causal Recurrent Variational Autoencoder for Medical Time Series Generation. Proc AAAI Conf Artif Intell. 2023 June;37(7):8562–70.

16. Pang C, Jiang X, Pavinkurve NP, Kalluri KS, Minto EL, Patterson J, et al. CEHR-GPT: Generating Electronic Health Records with Chronological Patient Timelines [Internet]. arXiv; 2024 [cited 2025 Mar 26]. Available from: http://arxiv.org/abs/2402.04400

17. Sun H, Lin H, Yan R. Collaborative Synthesis of Patient Records through Multi-Visit Health State Inference [Internet]. arXiv; 2023 [cited 2025 Mar 26]. Available from: http://arxiv.org/abs/2312.14646

18. Bing S, Dittadi A, Bauer S, Schwab P. Conditional Generation of Medical Time Series for Extrapolation to Underrepresented Populations [Internet]. arXiv; 2022 [cited 2025 Mar 26]. Available from: http://arxiv.org/abs/2201.08186

19. Theodorou B, Jain S, Xiao C, Sun J. ConSequence: Synthesizing Logically Constrained Sequences for Electronic Health Record Generation. Proc AAAI Conf Artif Intell. 2024 Mar;38(14):15355–63.

20. Wang L, Zhang W, He X. Continuous Patient-Centric Sequence Generation via Sequentially Coupled Adversarial Learning. In: Li G, Yang J, Gama J, Natwichai J, Tong Y, editors. Database Systems for Advanced Applications. Cham: Springer International Publishing; 2019. p. 36–52.

21. Liu Z, Liu X, Zhang X, Li J. Contrastive Learning-Based Time Series Classification in Healthcare. In: Proceedings of the 2023 4th International Symposium on Artificial Intelligence for Medicine Science [Internet]. New York, NY, USA: Association for Computing Machinery; 2024. p. 728–33. (ISAIMS ’23). Available from: https://doi.org/10.1145/3644116.3644238

22. Kallidromitis K, Gudovskiy D, Kozuka K, Ohama I, Rigazio L. Contrastive Neural Processes for Self-Supervised Learning [Internet]. arXiv; 2021 [cited 2025 Mar 26]. Available from: http://arxiv.org/abs/2110.13623

23. Torfi A, Fox EA. CorGAN: Correlation-Capturing Convolutional Generative Adversarial Networks for Generating Synthetic Healthcare Records [Internet]. arXiv; 2020 [cited 2025 Mar 26]. Available from: http://arxiv.org/abs/2001.09346

24. Choi S, Kim S. Data augmentation method for modeling health records with applications to clopidogrel treatment failure detection [Internet]. arXiv; 2024 [cited 2025 Mar 26]. Available from: http://arxiv.org/abs/2402.18046

25. Haleem MS, Ekuban A, Antonini A, Pagliara S, Pecchia L, Allocca C. Deep-Learning-Driven Techniques for Real-Time Multimodal Health and Physical Data Synthesis. Electronics. 2023 Jan;12(9):1989.

26. Kim J, Woo SH, Kim T, Yoon WT, Shin JH, Lee JY, et al. Development of a cerebellar ataxia diagnosis model using conditional GAN-based synthetic data generation for visuomotor adaptation task. BMC Med Inform Decis Mak. 2024 Nov;24(1):336.

27. Torfi A, Fox EA, Reddy CK. Differentially Private Synthetic Medical Data Generation using Convolutional GANs. Inf Sci. 2022 Mar;586:485–500.

28. Alcaraz JML, Strodthoff N. Diffusion-based Conditional ECG Generation with Structured State Space Models [Internet]. arXiv; 2023 [cited 2025 Mar 26]. Available from: http://arxiv.org/abs/2301.08227

29. Kaisti M, Laitala J, Wong D, Airola A. Domain randomization using synthetic electrocardiograms for training neural networks. Artif Intell Med. 2023 Sept 1;143:102583.

30. Habiba M, Brophy E, Pearlmutter BA, Ward T. ECG synthesis with Neural ODE and GAN models [Internet]. arXiv; 2022 [cited 2025 Mar 26]. Available from: http://arxiv.org/abs/2111.00314

31. Yoon J, Mizrahi M, Ghalaty NF, Jarvinen T, Ravi AS, Brune P, et al. EHR-Safe: generating high-fidelity and privacy-preserving synthetic electronic health records. Npj Digit Med. 2023 Aug;6(1):1–11.

32. Alawneh L, Alsarhan T, Al-Zinati M, Al-Ayyoub M, Jararweh Y, Lu H. Enhancing human activity recognition using deep learning and time series augmented data. J Ambient Intell Humaniz Comput. 2021 Dec;12(12):10565–80.

33. Biswal S, Ghosh S, Duke J, Malin B, Stewart W, Sun J. EVA: Generating Longitudinal Electronic Health Records Using Conditional Variational Autoencoders [Internet]. arXiv; 2020 [cited 2025 Mar 26]. Available from: http://arxiv.org/abs/2012.10020

34. Tarek MFB, Poulain R, Beheshti R. Fairness-Optimized Synthetic EHR Generation for Arbitrary Downstream Predictive Tasks [Internet]. arXiv; 2024 [cited 2025 Mar 26]. Available from: http://arxiv.org/abs/2406.02510

35. Steven Eyobu O, Han DS. Feature Representation and Data Augmentation for Human Activity Classification Based on Wearable IMU Sensor Data Using a Deep LSTM Neural Network. Sensors. 2018 Sept;18(9):2892.

36. Dissanayake T, Fernando T, Denman S, Sridharan S, Fookes C. Generalized Generative Deep Learning Models for Biosignal Synthesis and Modality Transfer. IEEE J Biomed Health Inform. 2023 Feb;27(2):968–79.

37. Zhou G, Barbieri S. Generating Clinically Realistic EHR Data via a Hierarchy- and Semantics-Guided Transformer [Internet]. arXiv; 2025 [cited 2025 Mar 26]. Available from: http://arxiv.org/abs/2502.20719

38. Wulan N, Wang W, Sun P, Wang K, Xia Y, Zhang H. Generating electrocardiogram signals by deep learning. Neurocomputing. 2020 Sept;404:122–36.

39. Shi J, Wang D, Tesei G, Norgeot B. Generating high-fidelity privacy-conscious synthetic patient data for causal effect estimation with multiple treatments. Front Artif Intell [Internet]. 2022 Sept [cited 2025 Apr 16];5. Available from: https://www.frontiersin.orghttps://www.frontiersin.org/journals/artificial-intelligence/articles/10.3389/frai.2022.918813/full

40. Seyfi A, Rajotte JF, Ng RT. Generating multivariate time series with COmmon Source CoordInated GAN (COSCI-GAN) [Internet]. arXiv; 2022 [cited 2025 Mar 26]. Available from: http://arxiv.org/abs/2205.13741

41. Hoorn R van, Bakkes T, Tokoutsi Z, Jong Y de, Bouwman RA, Pechenizkiy M. Generating Privacy-Preserving Longitudinal Synthetic Data. In 2023 [cited 2025 Apr 2]. Available from: https://openreview.net/forum?id=Xr13v66xxT

42. Vetter J, Macke JH, Gao R. Generating realistic neurophysiological time series with denoising diffusion probabilistic models. Patterns. 2024 Sept;5(9):101047.

43. Lee D, Yu H, Jiang X, Rogith D, Gudala M, Tejani M, et al. Generating sequential electronic health records using dual adversarial autoencoder. J Am Med Inform Assoc JAMIA. 2020 July;27(9):1411–9.

44. Kuo NIH, Garcia F, Sönnerborg A, Böhm M, Kaiser R, Zazzi M, et al. Generating synthetic clinical data that capture class imbalanced distributions with generative adversarial networks: Example using antiretroviral therapy for HIV. J Biomed Inform. 2023 Aug;144:104436.

45. Lange L, Wenzlitschke N, Rahm E. Generating Synthetic Health Sensor Data for Privacy-Preserving Wearable Stress Detection. Sensors. 2024 Jan;24(10):3052.

46. Li J, Cairns BJ, Li J, Zhu T. Generating Synthetic Mixed-type Longitudinal Electronic Health Records for Artificial Intelligent Applications [Internet]. arXiv; 2023 [cited 2025 Mar 26]. Available from: http://arxiv.org/abs/2112.12047

47. Wendland P, Birkenbihl C, Gomez-Freixa M, Sood M, Kschischo M, Fröhlich H. Generation of realistic synthetic data using Multimodal Neural Ordinary Differential Equations. Npj Digit Med. 2022 Aug;5(1):1–10.

48. Gall KL, Bellanger L, Laplaud D, Stamm A. Generation of synthetic gait data: application to multiple sclerosis patients’ gait patterns [Internet]. arXiv; 2024 [cited 2025 Mar 26]. Available from: http://arxiv.org/abs/2411.10377

49. Linial O, Ravid N, Eytan D, Shalit U. Generative ODE modeling with known unknowns. In: Proceedings of the Conference on Health, Inference, and Learning [Internet]. New York, NY, USA: Association for Computing Machinery; 2021. p. 79–94. (CHIL ’21). Available from: https://doi.org/10.1145/3450439.3451866

50. Zhu T, Li K, Herrero P, Georgiou P. GluGAN: Generating Personalized Glucose Time Series Using Generative Adversarial Networks. IEEE J Biomed Health Inform. 2023 Oct;27(10):5122–33.

51. Brouwer ED, Simm J, Arany A, Moreau Y. GRU-ODE-Bayes: Continuous modeling of sporadically-observed time series [Internet]. arXiv; 2019 [cited 2025 Mar 26]. Available from: http://arxiv.org/abs/1905.12374

52. Ghosheh GO, Li J, Zhu T. IGNITE: Individualized GeNeration of Imputations in Time-series Electronic health records [Internet]. arXiv; 2024 [cited 2025 Mar 26]. Available from: http://arxiv.org/abs/2401.04402

53. Lee M, Tae D, Choi JH, Jung HY, Seok J. Improved recurrent generative adversarial networks with regularization techniques and a controllable framework. Inf Sci. 2020 Oct;538:428–43.

54. Fallahzadeh R, Bidoki NH, Stelzer IA, Becker M, Marić I, Chang AL, et al. In-silico generation of high-dimensional immune response data in patients using a deep neural network. Cytometry A. 2023;103(5):392–404.

55. Alt EM, Qu Y, Damone E, Liu J ou, Wang C, Ibrahim JG. Jointly modeling time-to-event and longitudinal data with individual-specific change points: a case study in modeling tumor burden [Internet]. arXiv; 2024 [cited 2025 Mar 26]. Available from: http://arxiv.org/abs/2409.13873

56. Zhang Z, Yan C, Malin BA. Keeping synthetic patients on track: feedback mechanisms to mitigate performance drift in longitudinal health data simulation. J Am Med Inform Assoc. 2022 Nov;29(11):1890–8.

57. Ramchandran S, Tikhonov G, Lönnroth O, Tiikkainen P, Lähdesmäki H. Learning Conditional Variational Autoencoders with Missing Covariates [Internet]. arXiv; 2022 [cited 2025 Mar 26]. Available from: http://arxiv.org/abs/2203.01218

58. Schulz NA, Carus J, Wiederhold AJ, Johanns O, Peters F, Rath N, et al. Learning debiased graph representations from the OMOP common data model for synthetic data generation. BMC Med Res Methodol. 2024 June;24(1):136.

59. Ramchandran S, Tikhonov G, Kujanpää K, Koskinen M, Lähdesmäki H. Longitudinal Variational Autoencoder [Internet]. arXiv; 2021 [cited 2025 Mar 26]. Available from: http://arxiv.org/abs/2006.09763

60. Fisher CK, Smith AM, Walsh JR. Machine learning for comprehensive forecasting of Alzheimer’s Disease progression. Sci Rep. 2019 Sept;9(1):13622.

61. Zhong Y, Cui S, Wang J, Wang X, Yin Z, Wang Y, et al. MedDiffusion: Boosting Health Risk Prediction via Diffusion-based Data Augmentation [Internet]. arXiv; 2023 [cited 2025 Mar 26]. Available from: http://arxiv.org/abs/2310.02520

62. Wickstrøm K, Kampffmeyer M, Mikalsen KØ, Jenssen R. Mixing Up Contrastive Learning: Self-Supervised Representation Learning for Time Series. Pattern Recognit Lett. 2022 Mar;155:54–61.

63. Poyraz O, Marttinen P. Mixture of Coupled HMMs for Robust Modeling of Multivariate Healthcare Time Series [Internet]. arXiv; 2023 [cited 2025 Mar 26]. Available from: http://arxiv.org/abs/2311.07867

64. Ceritli T, Creagh AP, Clifton DA. Mixture of Input-Output Hidden Markov Models for Heterogeneous Disease Progression Modeling [Internet]. arXiv; 2022 [cited 2025 Mar 26]. Available from: http://arxiv.org/abs/2207.11846

65. Silva JF, Matos S. Modelling Patient Trajectories Using Multimodal Information [Internet]. arXiv; 2022 [cited 2025 Mar 26]. Available from: http://arxiv.org/abs/2209.04224

66. Lu C, Reddy CK, Wang P, Nie D, Ning Y. Multi-Label Clinical Time-Series Generation via Conditional GAN. IEEE Trans Knowl Data Eng. 2024 Apr;36(4):1728–40.

67. Moazemi S, Adams T, NG HG, Kühnel L, Schneider J, Näher AF, et al. NFDI4Health workflow and service for synthetic data generation, assessment and risk management [Internet]. arXiv; 2024 [cited 2025 Apr 2]. Available from: http://arxiv.org/abs/2408.04478

68. Kiyasseh D, Tadesse GA, Nhan LNT, Van Tan L, Thwaites L, Zhu T, et al. PlethAugment: GAN-Based PPG Augmentation for Medical Diagnosis in Low-Resource Settings. IEEE J Biomed Health Inform. 2020 Nov;24(11):3226–35.

69. Li K, Yang S, Sullivan TM, Burd RS, Marsic I. ProcessGAN: Generating Privacy-Preserving Time-Aware Process Data with Conditional Generative Adversarial Nets. ACM Trans Knowl Discov Data [Internet]. 2024 Nov;18(9). Available from: https://doi.org/10.1145/3687464

70. Wang Z, Sun J. PromptEHR: Conditional Electronic Healthcare Records Generation with Prompt Learning [Internet]. arXiv; 2022 [cited 2025 Apr 2]. Available from: http://arxiv.org/abs/2211.01761

71. Ashrafi N, Schmitt V, Spang RP, Möller S, Voigt-Antons JN. Protect and Extend – Using GANs for Synthetic Data Generation of Time-Series Medical Records [Internet]. arXiv; 2024 [cited 2025 Mar 26]. Available from: http://arxiv.org/abs/2402.14042

72. Yadav P, Gaur M, Fatima N, Sarwar S. Qualitative and Quantitative Evaluation of Multivariate Time-Series Synthetic Data Generated Using MTS-TGAN: A Novel Approach. Appl Sci. 2023 Jan;13(7):4136.

73. Yu K, Wang Y, Cai Y, Xiao C, Zhao E, Glass L, et al. Rare Disease Detection by Sequence Modeling with Generative Adversarial Networks [Internet]. arXiv; 2019 [cited 2025 Apr 16]. Available from: http://arxiv.org/abs/1907.01022

74. Hyland SL, Esteban C, Rätsch G. Real-valued (Medical) Time Series Generation with Recurrent Conditional GANs [Internet]. arXiv; 2017 [cited 2025 Mar 26]. Available from: http://arxiv.org/abs/1706.02633

75. Sood M, Sahay A, Karki R, Emon MA, Vrooman H, Hofmann-Apitius M, et al. Realistic simulation of virtual multi-scale, multi-modal patient trajectories using Bayesian networks and sparse auto-encoders. Sci Rep. 2020 July;10(1):10971.

76. Tian M, Chen B, Guo A, Jiang S, Zhang AR. Reliable Generation of Privacy-preserving Synthetic Electronic Health Record Time Series via Diffusion Models [Internet]. arXiv; 2024 [cited 2025 Mar 26]. Available from: http://arxiv.org/abs/2310.15290

77. Afrin K, Verma P, Srivatsa SS, Bukkapatnam STS. Simultaneous 12-Lead Electrocardiogram Synthesis using a Single-Lead ECG Signal: Application to Handheld ECG Devices [Internet]. arXiv; 2018 [cited 2025 Mar 26]. Available from: http://arxiv.org/abs/1811.08035

78. Sørensen K, Diez P, Margeta J, Youssef YE, Pham M, Pedersen JJ, et al. Spatio-temporal neural distance fields for conditional generative modeling of the heart [Internet]. arXiv; 2024 [cited 2025 Mar 26]. Available from: http://arxiv.org/abs/2407.10663

79. Hsieh TY, Sun Y, Tang X, Wang S, Honavar VG. SrVARM: State Regularized Vector Autoregressive Model for Joint Learning of Hidden State Transitions and State-Dependent Inter-Variable Dependencies from Multi-variate Time Series. In: Proceedings of the Web Conference 2021 [Internet]. New York, NY, USA: Association for Computing Machinery; 2021. p. 2270–80. (WWW ’21). Available from: https://doi.org/10.1145/3442381.3450116

80. Ming Y, Ding W, Pelusi D, Wu D, Wang YK, Prasad M, et al. Subject adaptation network for EEG data analysis. Appl Soft Comput. 2019 Nov;84:105689.

81. Norcliffe A, Cebere B, Imrie F, Lio P, Schaar M van der. SurvivalGAN: Generating Time-to-Event Data for Survival Analysis [Internet]. arXiv; 2023 [cited 2025 Mar 26]. Available from: http://arxiv.org/abs/2302.12749

82. Hazra D, Byun YC. SynSigGAN: Generative Adversarial Networks for Synthetic Biomedical Signal Generation. Biology. 2020 Dec;9(12):441.

83. Dahmen J, Cook D. SynSys: A Synthetic Data Generation System for Healthcare Applications. Sensors. 2019 Jan;19(5):1181.

84. Zhang Z, Yan C, Lasko TA, Sun J, Malin BA. SynTEG: a framework for temporal structured electronic health data simulation. J Am Med Inform Assoc JAMIA. 2021 Mar;28(3):596–604.

85. Walonoski J, Kramer M, Nichols J, Quina A, Moesel C, Hall D, et al. Synthea: An approach, method, and software mechanism for generating synthetic patients and the synthetic electronic health care record. J Am Med Inform Assoc. 2018 Mar;25(3):230–8.

86. Brophy E. Synthesis of Dependent Multichannel ECG using Generative Adversarial Networks. In: Proceedings of the 29th ACM International Conference on Information &amp; Knowledge Management [Internet]. New York, NY, USA: Association for Computing Machinery; 2020. p. 3229–32. (CIKM ’20). Available from: https://doi.org/10.1145/3340531.3418509

87. Delaney AM, Brophy E, Ward TE. Synthesis of Realistic ECG using Generative Adversarial Networks [Internet]. arXiv; 2019 [cited 2025 Mar 26]. Available from: http://arxiv.org/abs/1909.09150

88. Theodorou B, Xiao C, Sun J. Synthesize Extremely High-dimensional Longitudinal Electronic Health Records via Hierarchical Autoregressive Language Model. Res Sq. 2023 Mar 10;rs.3.rs-2644725.

89. Theodorou B, Xiao C, Sun J. Synthesize High-dimensional Longitudinal Electronic Health Records via Hierarchical Autoregressive Language Model. Nat Commun. 2023 Aug;14(1):5305.

90. Zhong Y, Wang X, Wang J, Zhang X, Wang Y, Huai M, et al. Synthesizing Multimodal Electronic Health Records via Predictive Diffusion Models. In: Proceedings of the 30th ACM SIGKDD Conference on Knowledge Discovery and Data Mining [Internet]. New York, NY, USA: Association for Computing Machinery; 2024. p. 4607–18. (KDD ’24). Available from: https://doi.org/10.1145/3637528.3671836

91. Foomani FH, Anisuzzaman DM, Niezgoda J, Niezgoda J, Guns W, Gopalakrishnan S, et al. Synthesizing time-series wound prognosis factors from electronic medical records using generative adversarial networks. J Biomed Inform. 2022 Jan;125:103972.

92. Kühnel L, Schneider J, Perrar I, Adams T, Moazemi S, Prasser F, et al. Synthetic data generation for a longitudinal cohort study – evaluation, method extension and reproduction of published data analysis results. Sci Rep. 2024 June;14(1):14412.

93. Nikolentzos G, Vazirgiannis M, Xypolopoulos C, Lingman M, Brandt EG. Synthetic electronic health records generated with variational graph autoencoders. Npj Digit Med. 2023 Apr;6(1):1–12.

94. Kulyabin M, Constable PA, Zhdanov A, Lee IO, Skuse DH, Thompson DA, et al. Synthetic Electroretinogram Signal Generation Using Conditional Generative Adversarial Network for Enhancing Classification of Autism Spectrum Disorder [Internet]. arXiv; 2024 [cited 2025 Mar 26]. Available from: http://arxiv.org/abs/2407.08166

95. Dash S, Dutta R, Guyon I, Pavao A, Yale A, Bennett KP. Synthetic Event Time Series Health Data Generation [Internet]. arXiv; 2019 [cited 2025 Mar 26]. Available from: http://arxiv.org/abs/1911.06411

96. Kuo NIH, Jorm L, Barbieri S. Synthetic Health-related Longitudinal Data with Mixed-type Variables Generated using Diffusion Models [Internet]. arXiv; 2023 [cited 2025 Mar 26]. Available from: http://arxiv.org/abs/2303.12281

97. Larrea X, Hernandez M, Epelde G, Beristain A, Molina C, Alberdi A, et al. Synthetic Subject Generation with Coupled Coherent Time Series Data. Eng Proc. 2022;18(1):7.

98. Hashemi AS, Etminani K, Soliman A, Hamed O, Lundström J. Time-series Anonymization of Tabular Health Data using Generative Adversarial Network. In: 2023 International Joint Conference on Neural Networks (IJCNN) [Internet]. 2023 [cited 2025 Apr 16]. p. 1–8. Available from: https://ieeexplore.ieee.org/document/10191367

99. Dubey M, Srijith PK, Desarkar MS. Time-to-Event Modeling with Hypernetwork based Hawkes Process. In: Proceedings of the 29th ACM SIGKDD Conference on Knowledge Discovery and Data Mining [Internet]. New York, NY, USA: Association for Computing Machinery; 2023. p. 3956–65. (KDD ’23). Available from: https://doi.org/10.1145/3580305.3599912

100. Lee G, Yu W, Shin K, Cheng W, Chen H. TimeCAP: Learning to Contextualize, Augment, and Predict Time Series Events with Large Language Model Agents [Internet]. arXiv; 2025 [cited 2025 Mar 26]. Available from: http://arxiv.org/abs/2502.11418

101. Chang C, Chan CT, Wang WY, Peng WC, Chen TF. TimeDRL: Disentangled Representation Learning for Multivariate Time-Series [Internet]. arXiv; 2024 [cited 2025 Mar 26]. Available from: http://arxiv.org/abs/2312.04142

102. Qian J, Xie B, Wan B, Li M, Sun M, Chiang PY. TimeLDM: Latent Diffusion Model for Unconditional Time Series Generation [Internet]. arXiv; 2024 [cited 2025 Mar 26]. Available from: http://arxiv.org/abs/2407.04211

103. Song Z, Lu Q, Xu H, Zhu H, Buckeridge D, Li Y. TimelyGPT: Extrapolatable Transformer Pre-training for Long-term Time-Series Forecasting in Healthcare. In: Proceedings of the 15th ACM International Conference on Bioinformatics, Computational Biology and Health Informatics [Internet]. New York, NY, USA: Association for Computing Machinery; 2024. (BCB ’24). Available from: https://doi.org/10.1145/3698587.3701364

104. Gao C, Beigi M, Shafquat A, Aptekar J, Sun J. TrialSynth: Generation of Synthetic Sequential Clinical Trial Data [Internet]. arXiv; 2024 [cited 2025 Mar 26]. Available from: http://arxiv.org/abs/2409.07089

105. Yalavarthi VK, Burchert J, Schmidt-thieme L. Tripletformer for Probabilistic Interpolation of Irregularly sampled Time Series [Internet]. arXiv; 2024 [cited 2025 Mar 26]. Available from: http://arxiv.org/abs/2210.02091

106. Yang Z, Li Y, Zhou G. TS-GAN: Time-series GAN for Sensor-based Health Data Augmentation. ACM Trans Comput Healthc [Internet]. 2023 Apr;4(2). Available from: https://doi.org/10.1145/3583593

107. Li X, Ngu AHH, Metsis V. TTS-CGAN: A Transformer Time-Series Conditional GAN for Biosignal Data Augmentation [Internet]. arXiv; 2022 [cited 2025 Mar 26]. Available from: http://arxiv.org/abs/2206.13676

108. Li X, Metsis V, Wang H, Ngu AHH. TTS-GAN: A Transformer-based Time-Series Generative Adversarial Network [Internet]. arXiv; 2022 [cited 2025 Mar 26]. Available from: http://arxiv.org/abs/2202.02691

109. Wang Y, Fu T, Xu Y, Ma Z, Xu H, Du B, et al. TWIN-GPT: Digital Twins for Clinical Trials via Large Language Model. ACM Trans Multimed Comput Commun Appl [Internet]. 2024 July; Available from: https://doi.org/10.1145/3674838

110. Das T, Wang Z, Sun J. TWIN: Personalized Clinical Trial Digital Twin Generation. In: Proceedings of the 29th ACM SIGKDD Conference on Knowledge Discovery and Data Mining [Internet]. New York, NY, USA: Association for Computing Machinery; 2023 [cited 2025 Apr 16]. p. 402–13. (KDD ’23). Available from: https://dl.acm.org/doi/10.1145/3580305.3599534

111. Li J, Peng J, Li H, Chen L. UniCL: A Universal Contrastive Learning Framework for Large Time Series Models [Internet]. arXiv; 2024 [cited 2025 Mar 26]. Available from: http://arxiv.org/abs/2405.10597

112. Wang X, Lin Y, Xiong Y, Zhang S, He Y, He Y, et al. Using an optimized generative model to infer the progression of complications in type 2 diabetes patients. BMC Med Inform Decis Mak. 2022 July;22(1):174.

113. Moore JB, Stackhouse HP, Fulcher BD, Mahmoodian S. Using matrix-product states for time-series machine learning [Internet]. arXiv; 2024 [cited 2025 Mar 26]. Available from: http://arxiv.org/abs/2412.15826

114. Gootjes-Dreesbach L, Sood M, Sahay A, Hofmann-Apitius M, Fröhlich H. Variational Autoencoder Modular Bayesian Networks for Simulation of Heterogeneous Clinical Study Data. Front Big Data [Internet]. 2020 May [cited 2025 Apr 2];3. Available from: https://www.frontiersin.org/journals/big-data/articles/10.3389/fdata.2020.00016/full

115. Arabi D, Bakhshaliyev J, Coskuner A, Madhusudhanan K, Uckardes KS. Wave-Mask/Mix: Exploring Wavelet-Based Augmentations for Time Series Forecasting [Internet]. arXiv; 2024 [cited 2025 Mar 26]. Available from: http://arxiv.org/abs/2408.10951

1. “None” under privacy mechanism denotes studies that did not implement a formal or technical privacy-preserving method. These studies were still included if they provided at least a utility evaluation, consistent with the inclusion criteria requiring either utility and/or privacy assessment. [↑](#footnote-ref-1)
