# Supplementary material for: Synthetic data generation methods for longitudinal and time series health data: a systematic review
Source: BMC Med Inform Decis Mak. 2025 Dec 24;26:30. doi: 10.1186/s12911-025-03326-8 (PMC12849454; doi:10.1186/s12911-025-03326-8)
Supplement: Supplementary file 2 — Supplementary Material 2 [file 12911_2025_3326_MOESM2_ESM.docx]

Table S2: Overview of public datasets used in reviewed studies. Each dataset is categorized by temporal data type, modality, and its use across included studies.

| Dataset | Type | Data Modality (detailed) | Used by studies |
| --- | --- | --- | --- |
| Sleep-EDF (1) | C-R + E | Single-channel EEG (Fpz-Cz) | You et al. (2), Yang et al. (3), Hazra and Byun (4), Song et al. (5), |
| Synthetic OCT–Longitudinal Dataset (6) | C-IR | Imaging (OCT scans – 2D grayscale); Simulated longitudinal clinical trajectories | Deltadahl et al. (7) |
| BAGLS (8) | C-R | High-speed laryngeal videoendoscopy; Derived Glottal Area Waveform (GAW) | Darvish and Kist (9) |
| Synthetic Alberta Health Administrative Data (10) | E | Coded clinical events; Temporal event sequences modeled using a knowledge graph and transition probabilities | Mosquera et al. (11) |
| PhysioNet 2017 Challenge Dataset (12) | C-R | Single-lead ECG signals (sampling rate: 300 Hz) | Cao et al.(13) |
| WISDM (14) | C-R | Accelerometer and gyroscope data from smartphone & smartwatch | Boukhennoufa et al. (15), Alawneh et al. (16), Chang et al. (17) |
| Post-Stroke Sensor Dataset (18) | C-IR | Wearable sensors & vision-based (camera) time-series (e.g., gait, motion) | Boukhennoufa et al. (15) |
| UCI-PAMAP2 (19) | C-R | Accelerometer, gyroscope, heart rate (wearables, 100 Hz) | Wang et al. (20) |
| HASC2010 corpus (21) | C-R | Smartphone accelerometer data (3-axis, labeled activities) | Wang et al. (20) |
| PAF Prediction Challenge Database (22) | C-R | Single-lead ECG signals (sampling rate: 300 Hz) | Asadi et al. (23) |
| HASC2011 corpus (24) | C-R | Tri-axial accelerometer signals from smartphones | Li et al. (25) |
| Catharina hospital (Eindhoven, the Netherlands) | C-R | Ventilator waveform data (pressure and flow signals) | Hao et al. (26) |
| Fondazione I.R.C.C.S. Policlinico San Matteo (Pavia, Italy) | C-R | Ventilator waveform data (pressure and flow signals) | Hao et al. (26) |
| Simulated Dataset (27) | C-R | Simulated Pressure Support Ventilation (PSV) Waveforms | Hao et al. (26) |
| UAE Cardiovascular Risk Cohort Dataset (28) | E | Administrative and clinical records | Kuo et al. (29) |
| Apnea-ECG (30) (31) | C-R | Single-lead ECG (100 Hz) + apnea annotations (per-minute) | Nikolaidis et al. (32), Yang et al. (3) |
| MIT-BIH (33) | C-R | Multichannel polysomnography: EEG, ECG, respiration, EMG, EOG, SaO₂, etc. | Nikolaidis et al. (32), Kallidromitis et al. (34), Hazra and Byun (4), Dissanayake et al. (35), Habiba et al. (36), |
| MIT-BIH Arrythmia (37) | C-R | ECG signals (cardiac arrhythmia detection) | Torfi et al. (38), Zhu et al. (39), Wulan et al. (40), Zhu et al. (41), Delaney et al. (42), Brophy (43), Li et al. (44), Li et al. (45) |
| MIT-BIH Normal Sinus Rhythm Database (46) | C-R | Clean ECG recordings | Habiba et al. (36) |
| TREND cohort data | C-IR + E | Clinical visit data over time; Longitudinal biomarker assessments; Variable visit intervals and diverse data types | Sood et al. (47) |
| Congestive Heart Failure (Heart Failure) Cohort | E | EHR-derived symbolic events (diagnoses, medications, procedures) | Che et al. (48) |
| Diabetes Cohort | E | EHR-derived symbolic events (diagnoses, medications, procedures) | Che et al. (48) |
| Hénon maps | C-R | Simulated 2D nonlinear dynamical system (chaotic) | Li et al. (49) |
| Lorenz-96 model | C-R | Simulated 3D continuous chaotic system (x, y, z trajectories) | Li et al. (49) |
| fMRI (50) | C-R | Simulated BOLD signal time series across multiple brain regions | Li et al. (49), Qian et al. (204) |
| EEG (51) | C-R | Multichannel high‑frequency neural signal time series | Li et al. (49) |
| Columbia University Irving Medical Center-New York Presbyterian Hospital EHR data (OMOP database) | E | Structured clinical events; Precise timestamps; Serialized as chronological token sequences | Pang et al. (52) |
| MIMIC-III (53) | E | Structured EHR events: diagnoses, procedures, labs, medications, vitals | w |
| MIMIC-III – Heart Failure (53) | C-R | ICU Time Series (Vitals, Labs, Diagnoses) | Zhong et al. (73) |
| MIMIC-IV (74) | E | Structured EHR events: diagnoses, procedures, labs, medications, vitals | Sun et al. (54), Lu et al. (67), Tian et al. (75), Nikolentzos et al. (76), Hashemi et al. (77), Zhou and Barbieri (62), Tian et al. (75) |
| outpatient EHR dataset | E | Longitudinal outpatient claims data | Theodorou et al. (56) |
| Epilepsy (78) | C-R | EEG signals, 1-second samples, seizure classification | Liu et al. (58) |
| HAR (79) | C-R | Accelerometer & gyroscope data from wearables (50 Hz) | Liu et al. (58), Chang et al. (17) |
| IMS Bearings (80) | C-R | Accelerometer signals (bearing fault detection) | Kallidromitis et al. (34) |
| Urban8K (81) | C-R | Environmental audio waveforms (urban sound classification) | Kallidromitis et al. (34) |
| UCI Epileptic Seizure Recognition dataset (82) | E | Structured EHR events: diagnoses, procedures, labs, medications, vitals | Torfi and Fox (59) |
| UK Biobank (83) | E | Diagnosis, procedure, and prescription codes with timestamps | Choi and Kim (84) |
| GATEKEEPER PROJECT Dataset (85) | C-R; C-IR; E | Smartphones, self-assessments, and wearables data | Haleem et al. (86) |
| visuomotor adaptation dataset (87) | C-IR; E | visuomotor adaptation data | Kim et al. (88) |
| Kaggle Cervical Cancer (89) | E | Structured Clinical Data (Survey/Screening) | Torfi et al. (38) |
| UCI Epileptic Seuizure Recognition (82) | C-R | ECG | Torfi et al. (38) |
| PTB Diagnostic ECG (90) | C-R | ECG | Torfi et al. (38), Afrin et al. (91), Li et al. (45) |
| Kaggle Cardiovascular Disease (92) | E | Structured Clinical Data (Tabular) | Torfi et al. (38) |
| PTB-XL (93) | C-R | ECG (12-lead) | Alcatraz and Strodthoff (94), Yang et al. (3) |
| eICU (95) | C-R | Vitals, Labs, Treatments, Survival Outcomes | Yoon et al. (60), Hoorn et al. (96), Li et al. (64), Ghosheh et al. (97), Hyland et al. (98), Tian et al. (75) |
| MMIDB-EEG (99) | C-R | EEG | Yang et al. (3) |
| CLAS (100) | C-R | EDA, ECG, PPG, ACC | Yang et al. (3) |
| PAMAP2 (19) | C-R | IMU (accelerometer, gyroscope, magnetometer, temp), HR | Yang et al. (3) |
| UCI-HAR (101) | C-R | Accelerometer, Gyroscope | Yang et al. (3), Alawneh et al. (16) |
| 10-years of longitudinal medical records | E | EHR Data | Biswal et al. (102) |
| PIC (Pediatric Intensive Care) dataset (103,104) | C-R | Vital signs (heart rate, SpO₂, blood pressure, respiratory rate), labs, meds, fluid balance, demographics | Eyobu and Han (105) |
| EPHNOGRAM (106) |  | ECG | Dissanayake et al. (35) |
| Data from large insurance company in the United States |  | diagnoses, procedures, and drug prescriptions or refills) and lab test results | Shi et al. (107) |
| Toy Sine Dataset | C-R | EEG | Seyfi et al. (108) |
| Sine Waves (109) | C-R | Synthetic (Sine signal) | Hoorn et al. (96) |
| In-patient Deterioration (Catharina Hospital) (110) | C-IR | Vitals, Labs, Demographics, Categorical Clinical States | Hoorn et al. (96) |
| Rat LFP Dataset (111) | C-R | LFP (Prefrontal Cortex, Thalamus, Hippocampus) | Vetter et al. (112) |
| AJILE12 (Human ECoG) (113) | C-R | ECoG | Vetter et al. (112) |
| Macaque ECoG (Awake/Anesthetized) | C-R | ECoG | Vetter et al. (112) |
| UT Physicians clinical database (UTP) | E | Structured EHR events: diagnoses, procedures, labs, medications, vitals | Lee et al. (63) |
| EuResist Integrated DataBase (EIDB) (114) | E | Genotype (viral sequences), Treatment regimens, Virological response outcomes | Kuo et al. (115) |
| WESAD (Wearable Stress and Affect Detection) (116) | C-R | ECG, EDA, EMG, RESP, TEMP, ACC (chest @700Hz); BVP (PPG), EDA, TEMP, ACC (wrist @4–64Hz) | Lange et al. (117) |
| HiRID (118) | C-R | High-res Vitals, Labs, Interventions (IV meds, devices), Outcomes | Li et al. (64), Ghosheh et al. (97) |
| PPMI (119) | C-IR | Clinical, Neurological, Demographics (PD-focused) | Wendland et al. (120), Sood et al. (121), Gootjes-Dreesbach et al. (122) |
| NACC (123) | C-IR | Clinical, Cognitive, Demographics (AD-focused) | Wendland et al. (120) |
| MYO (124) | C-R | Wearable Gait Signals (IMU), Disability Scores (EDSS) | Gall et al. (125) |
| Double Pendelum dataset |  |  | Linial et al. (126) |
| United States Historical Climatology Network (USHCN) (127) | C-IR | Climate: Precipitation, Snowfall, Snow Depth, Max/Min Temperature | Brouwer et al. (65) |
| PhysioNet 2012 (128) | C-IR | ICU Vitals, Labs, Demographics | Ghosheh et al. (97), Ramchandran et al. (129), Yalavarthi et al. (72) |
| PhysioNet 2019 (130) | C-IR | ICU time series for sepsis detection (38 variables, ~40,000 samples) | Yalavarthi et al. (72) |
| PhysioNet 2015 Challenge PPG (46) | C-R | PPG (resampled to 100 Hz) | Kiyasseh et al. (131) |
| Air Quality (AQ) (132) | C-R | Air pollutants (CO, NOx, NO₂, etc.), Temperature, Humidity | Lee et al. (133) |
| Beijing PM2.5 (BPM) (134) | C-R | PM2.5, Weather variables (Temp, Pressure, Wind Speed, etc.) | Lee et al. (133) |
| THA Mass Cytometry Study | E | Mass Cytometry (CyTOF), Clinical Metadata | Fallahzadeh et al. (135) |
| Simulated Clinical Trial Data | C-IR | Simulated Covariates, Longitudinal Outcomes, Event Times | Alt et al. (136) |
| VUMC EHR (PheWAS) (137) | E | Diagnosis Codes (PheWAS) | Zhang et al. (138) |
| All of Us EHR (139) | E | Diagnosis & Procedure Codes (CCS) | Zhang et al. (138) |
| Rotated Digits (Synthetic) (140) | C-R | Image (MNIST), Synthetic Covariates (Rotation, Shift, Contrast, Time) | Ramchandran et al. (141) |
| Prostate Cancer Trial (Project Datasphere) (142) | C-IR | Lab Results, Vitals, Adverse Events, Medications, Demographics | Ramchandran et al. (141) |
| Schleswig-Holstein Lung Cancer Registry (OMOP) | E | Diagnoses, Procedures (OMOP CDM), Structured Temporal Rules | Schulz et al. (143) |
| Rotated MNIST (3s only) | C-R | Image (MNIST digit '3'), Rotation Angle, ID | Ramchandran et al. (129) |
| Health MNIST | C-R | Image (MNIST digits ‘3’ and ‘6’), Disease Covariates, Age, Location | Ramchandran et al. (129) |
| CAMD (CODR-AD) (144) | C-R | Cognitive scores (ADAS-Cog, MMSE), Labs, Vitals, Demographics, History, Questionnaires | Fisher et al. (145) |
| TriNetX (146) | C-R | EHR Time Series (Vitals, Labs, Diagnoses) | Zhong et al. (73) |
| UCR Archive (147) | C-R | Univariate Time Series (UTS) | Wickstrøm et al. (148) |
| UEA Archive (149) | C-R | Multivariate Time Series (MTS) | Wickstrøm et al. (148) |
| CLEAR (RCT) (150) | C-IR | Binary colonization status (nares, throat, skin) over time | Poyraz and Marttinen (151) |
| Real-world Dataset for mIOHMM (152) | C-IR | EHR-like Time Series (Disease States, Observations) | Ceritli et al. (153) |
| Synthetic mPHMM Dataset (153) | C-R | Simulated Disease Progression States, Personalized Effects | Ceritli et al. (153) |
| Glioblastoma Synthetic Dataset (154) | E | Cross-sectional Oncology Data (9 variables) | Moazemi et al. (155) |
| ADNI (156) | C-IR | Clinical, Imaging, Cognitive Assessments (76 vars) | Moazemi et al. (155), Sood et al. (121) |
| Vietnam HFMD PPG | C-R | PPG (toe, 100 Hz) | Kiyasseh et al. (131) |
| Vietnam Tetanus PPG | C-R | PPG (finger, 100 Hz) | Kiyasseh et al. (131) |
| China CVD PPG (157) | C-R | PPG (1 kHz) | Kiyasseh et al. (131) |
| Pediatric Trauma | E | Manually coded clinical events | Li et al. (158) |
| Sepsis Cases Event Log | E | EHR-derived process events (time-stamped logs) | Li et al. (158) |
| Loan Application Event Log | E | Business process event traces | Li et al. (158) |
| Dementia Game Interaction (PflegeTab) (159) | C-R | Touchscreen behavior, QoL metrics, MMST scores, Task timing | Ashrafi et al. (160) |
| Google Stocks (2004–2021) (161) | C-R | Daily OHLC Prices, Volume, Adjusted Close | Yadav et al. (162) |
| UniMiB-SHAR (Running vs. Jumping) (163) | C-R | Accelerometer (3-axis), 150 timesteps per sample | Yadav et al. (162) |
| IQVIA EPI Claims Dataset | E | Diagnoses (Dx), Procedures, Prescriptions (Rx) | Yu et al. (164) |
| MNIST (165) | C‑R | 28×28‑pixel grayscale images of handwritten digits (60 k train / 10 k test) | Hyland et al. (98) |
| TWA Database (166) | C-R | 12-lead ECG | Afrin et al. (91) |
| Kardia + Paper ECGs | C-R | Kardia ECG (asynchronous), Paper ECG (digitized) | Afrin et al. (91) |
| TWA Database (166) | C-R | 12-lead ECG | Sørensen et al. (167) |
| Stress Data Base (SDB) (168) | E | Wrist-worn biosensors (EDA, HR, SpO₂, Temp, Acc, etc.) | Hsieh et al. (169) |
| BCI Competition IV Dataset 2a (170) | C-R | EEG | Ming et al. (171) |
| Quebec CHD Database | E | Administrative claims (ICD-9 codes, demographics, vital status) | Song et al. (172) |
| PopHR - COPD Subset | E | Administrative claims (ICD-9 codes, billing, hospitalization) | Song et al. (172), Song et al. (5) |
| ACTG 320 (173) | C-R | Clinical trial (HIV/AIDS) | Norcliffe et al. (174) |
| CUTRACT | C-R | Clinical records (Prostate cancer, UK) | Norcliffe et al. (174) |
| PHEART | C-R | EHR (Heart failure) | Norcliffe et al. (174) |
| SEER (175) | C-R | Cancer registry (Prostate cancer, US) | Norcliffe et al. (174) |
| METABRIC (176) | C-R | Genomics + clinical (Breast cancer) | Norcliffe et al. (174) |
| Siena Scalp EEG Database (177) | C-R | EEG (electroencephalogram) | Hazra and Byun (4) |
| BIDMC PPG and Respiration Dataset (178) | C-R | PPG (photoplethysmography) | Hazra and Byun (4) |
| Synthetic Derivative (SD) and Clean SD (CSD) (179,180) | E | Clinical codes (ICD → PheWAS) | Zhang et al. (181) |
| Synthetic Sine Waves | C-R | Simulated oscillatory signals (sinusoids) | Delaney et al. (42) |
| Outpatient EHR | E | Visit-level ICD-10 codes, demographics, disease phenotypes | Theodorou et al. (69) |
| Breast Cancer Trial (182) | E | Adverse events, medications, labs, treatments, demographics | Zhong et al. (70) |
| EMR VLU Dataset | C-IR | Wound measurements (length, width, area), wound assessments, clinical data (BP, BMI, history), demographics | Foomani et al. (183) |
| DONALD Study Dataset (184) | C-IR | Nutrition intake, anthropometrics, socioeconomic factors (longitudinal) | Kühnel et al. (185) |
| LA-ERG Dataset (186) | C-R | Electroretinogram (ERG) waveforms under 9 flash strengths | Kulyabin et al. (187) |
| ATUS Sleep Pattern Dataset (188) | E | Time-use diary data (minutes asleep per hour), demographics | Dash et al. (189) |
| ART for HIV (EuResist) (190) | C-IR | HIV therapy data (VL, CD4, meds over 10–100 months) | Kuo et al. (71) |
| TMET (Treadmill Maximal Exercise Tests) (46,191) | C-IR | Cardiorespiratory signals: HR, VO2, VCO2, RR, VE, treadmill speed, environmental metadata | Larrea et al. (192) |
| Yelp Events (193) | E | Check-in event times + business attributes (sequence descriptors: 1229-dim) | Dubey et al. (194) |
| Meme Events (195) | E | Media quote timestamps + content embeddings (sequence descriptors: Doc2Vec 100-dim) | Dubey et al. (194) |
| NY Weather | C-R | Hourly temp, humidity, pressure, wind (rain event prediction) | Lee et al. (196) |
| SF Weather | C-R | Hourly temp, humidity, pressure, wind (rain event prediction) | Lee et al. (196) |
| \| HS Weather \| \| --- \| | \| HS Weather \| \| --- \| | \| HS Weather \| \| --- \| | Lee et al. (196) |
| Finance-SP | C-R | Daily financial indicators (SP event prediction) | Lee et al. (196) |
| Finance-NK | C-R | Daily financial indicators (NK event prediction) | Lee et al. (196) |
| Mortality (MT) | C-R | Weekly influenza & pneumonia deaths (mortality threshold prediction) | Lee et al. (196) |
| Test-Positive (TP) | C-R | Weekly influenza A & B positive specimens (positivity threshold prediction) | Lee et al. (196) |
| ETT (ETTh1, ETTh2, ETTm1, ETTm2) (197) | C-R | Oil temperature + power loads (hourly & 15-min) | Chang et al. (17), Arabi et al. (224), Li et al. (216), Qian et al. (204) |
| Exchange (198) | C-R | Daily FX rates (8 countries) | Chang et al. (17) |
| Weather (199) | C-R | Daily local climatology: 11 weather variables incl. wet bulb | Chang et al. (17) |
| Epilepsy (201) | C-R | EEG (174 Hz) on epilepsy vs non-epilepsy | Chang et al. (17) |
| PenDigits (202) | C-R | x-y coordinates of handwritten digits | Chang et al. (17) |
| FingerMovements (203) | C-R | Key typing movement time series | Chang et al. (17) |
| Sine (109) | C-R | Simulated sinusoidal sequences (5 features with independent frequencies & phases) | Qian et al. (204) |
| MuJoCo (205) | C-R | Multivariate physics simulation (14 features) | Qian et al. (204) |
| Stocks (206) | C-R | Daily Google stock prices (6 features, 2004–2019) | Qian et al. (204) |
| LC1, BC1, CC, BC2, BC3, VTE, LC2 (182) | E | Clinical trial event logs (medications, procedures, adverse events over time) | Gao et al. (208) |
| PenDigits (synthetic) (209) | C-R | Synthetic sparse time series from digit trajectories | Yalavarthi et al. (72) |
| PhonemeSpectra (synthetic) (209) | C-R | Synthetic sparse time series from phoneme spectra | Yalavarthi et al. (72) |
| ECG_200 (210) | C-R | ECG time series (96 samples per heartbeat) | Yang et al. (210) |
| NonInvasiveFatalECG_Thorax1 (210) | C-R | Fetal ECG time series (750 samples) | Yang et al. (210) |
| mHealth (211) | C-R | Multisensor body motion & ECG (23 dims, chest + wearable sensors) | Yang et al. |
| Simulated Sine Waves | C-R | Synthetic multivariate sinusoidal sequences | Li et al. (44), Li et al. (45) |
| UniMiB HAR (163) | C-R | Accelerometer (x, y, z) human activity signals | Li et al. (44), Li et al. (45) |
| PTB Diagnostic ECG Database (212) | C-R | ECG heartbeats (normal vs abnormal) | Li et al. (44), Li et al. (45) |
| Phase III breast cancer trial (142) | E | Clinical trial survival data (DFS, treatments) | Wang et al. (213) |
| TOP (Trial Outcome Prediction) (214) | E | Multimodal clinical trial metadata & outcomes | Wang et al. (213) |
| Breast Cancer Trial (NCT00174655) (142) | E | Clinical trial: treatments, adverse events, medications | Das et al. (215) |
| Lung Cancer Trial (NCT01439568) (142) | E | Clinical trial: treatments, adverse events, medications | Das et al. (215) |
| Weather (217) | C-R | Weather variables (temperature, humidity, etc.) | Li et al. (216), Arabi et al. (224) |
| Electricity (218) | C-R | Electricity consumption (load diagrams) | Li et al. (216) |
| ILI (219) | C-R | Influenza-like illness rates | Li et al. (216) |
| 10 UEA datasets (149) | C-R | Various multivariate sensor / signal data (time-series classification) | Li et al. (216) |
| Shanghai T2D EHR dataset | C-IR | Longitudinal EHR records (ICD-10 codes over time), type 2 diabetes progression | Wang et al. (220) |
| NTS1–NTS6 | C-R | Synthetic time series: noisy trendy sinusoids (period, trend, phase, noise) | Moore et al. (221) |
| ECG200 (222) | C-R | ECG signals (heartbeats, healthy vs myocardial infarction) | Moore et al. (221) |
| ItalyPowerDemand | C-R | Power demand (daily load curves, winter vs summer) | Moore et al. (221) |
| KeplerLightCurves (223) | C-R | Stellar brightness light curves (variable star types) | Moore et al. (221) |
| ILI | C-R | Influenza-like illness rates over time | Arabi et al. (224) |

References

1. Kemp B, Aeilko Zwinderman, Tuk B, Kamphuisen H, Oberyé J. The Sleep-EDF Database [Expanded] [Internet]. physionet.org; 2018 [cited 2025 Jun 23]. Available from: https://physionet.org/content/sleep-edfx/

2. You Y, Guo X, Zhong X, Yang Z. A Few-Shot Learning-Based EEG and Stage Transition Sequence Generator for Improving Sleep Staging Performance. Biomedicines. 2022 Nov;10(12):3006.

3. Yang H, Yu H, Sano A. Empirical Evaluation of Data Augmentations for Biobehavioral Time Series Data with Deep Learning [Internet]. arXiv; 2022 [cited 2025 Mar 26]. Available from: http://arxiv.org/abs/2210.06701

4. Hazra D, Byun YC. SynSigGAN: Generative Adversarial Networks for Synthetic Biomedical Signal Generation. Biology. 2020 Dec;9(12):441.

5. Song Z, Lu Q, Xu H, Zhu H, Buckeridge D, Li Y. TimelyGPT: Extrapolatable Transformer Pre-training for Long-term Time-Series Forecasting in Healthcare. In: Proceedings of the 15th ACM International Conference on Bioinformatics, Computational Biology and Health Informatics [Internet]. New York, NY, USA: Association for Computing Machinery; 2024. (BCB ’24). Available from: https://doi.org/10.1145/3698587.3701364

6. Kermany D, Zhang K, Goldbaum M. Large Dataset of Labeled Optical Coherence Tomography (OCT) and Chest X-Ray Images. 2018 Jun 1 [cited 2025 Jun 24];3. Available from: https://data.mendeley.com/datasets/rscbjbr9sj/3

7. Deltadahl S, Vall A, Ivaturi V, Korsbo N. A Framework for Evaluating Predictive Models Using Synthetic Image Covariates and Longitudinal Data [Internet]. arXiv; 2024 [cited 2025 Mar 26]. Available from: http://arxiv.org/abs/2410.16177

8. Benchmark for Automatic Glottis Segmentation (BAGLS) [Internet]. [cited 2025 Jun 23]. Available from: https://www.bagls.org/

9. Darvish M, Kist AM. A Generative Method for a Laryngeal Biosignal. J Voice [Internet]. 2024 Feb [cited 2025 Apr 11]; Available from: https://www.sciencedirect.com/science/article/pii/S0892199724000195

10. Sharma V, Weir D, Samanani S, Simpson SH, Gilani F, Jess E, et al. Characterisation of concurrent use of prescription opioids and benzodiazepine/Z-drugs in Alberta, Canada: a population-based study. BMJ Open. 2019 Sep 1;9(9):e030858.

11. Mosquera L, El Emam K, Ding L, Sharma V, Zhang XH, Kababji SE, et al. A method for generating synthetic longitudinal health data. BMC Med Res Methodol. 2023 Mar;23(1):67.

12. Clifford G, Liu C, Moody B, Lehman L wei, Silva I, Li Q, et al. AF Classification from a Short Single Lead ECG Recording: the Physionet Computing in Cardiology Challenge 2017. In 2017 [cited 2025 Jun 25]. Available from: http://www.cinc.org/archives/2017/pdf/065-469.pdf

13. Cao P, Li X, Mao K, Lu F, Ning G, Fang L, et al. A novel data augmentation method to enhance deep neural networks for detection of atrial fibrillation. Biomed Signal Process Control. 2020 Feb;56:101675.

14. Weiss G. WISDM Smartphone and Smartwatch Activity and Biometrics Dataset [Internet]. UCI Machine Learning Repository; 2019 [cited 2025 Jun 25]. Available from: https://archive.ics.uci.edu/dataset/507

15. Boukhennoufa I, Jarchi D, Zhai X, Utti V, Sanei S, Lee TKM, et al. A Novel Model to Generate Heterogeneous and Realistic Time-Series Data for Post-Stroke Rehabilitation Assessment. IEEE Trans Neural Syst Rehabil Eng. 2023;31:2676–87.

16. Alawneh L, Alsarhan T, Al-Zinati M, Al-Ayyoub M, Jararweh Y, Lu H. Enhancing human activity recognition using deep learning and time series augmented data. J Ambient Intell Humaniz Comput. 2021 Dec;12(12):10565–80.

17. Chang C, Chan CT, Wang WY, Peng WC, Chen TF. TimeDRL: Disentangled Representation Learning for Multivariate Time-Series [Internet]. arXiv; 2024 [cited 2025 Mar 26]. Available from: http://arxiv.org/abs/2312.04142

18. Razfar N, Kashef R, Mohammadi F. Automatic Post-Stroke Severity Assessment Using Novel Unsupervised Consensus Learning for Wearable and Camera-Based Sensor Datasets. Sensors. 2023 Jun 12;23(12):5513.

19. Reiss A. PAMAP2 Physical Activity Monitoring [Internet]. UCI Machine Learning Repository; 2012 [cited 2025 Jun 25]. Available from: https://archive.ics.uci.edu/dataset/231

20. Wang J, Chen Y, Gu Y. A wearable-HAR oriented sensory data generation method based on spatio-temporal reinforced conditional GANs. Neurocomputing. 2022 Jul;493:548–67.

21. HASC2010corpus [Internet]. [cited 2025 Jun 25]. Available from: http://hasc.jp/hc2010/HASC2010corpus/hasc2010corpus-en.html

22. Moody GB, Goldberger AL, McClennen S, Swiryn SP. PAF Prediction Challenge Database [Internet]. physionet.org; 2001 [cited 2025 Jun 25]. Available from: https://physionet.org/content/afpdb/

23. Asadi M, Poursalim F, Loni M, Daneshtalab M, Sjödin M, Gharehbaghi A. Accurate detection of paroxysmal atrial fibrillation with certified-GAN and neural architecture search. Sci Rep. 2023 Jul;13:11378.

24. Kawaguchi N, Yang Y, Yang T, Ogawa N, Iwasaki Y, Kaji K, et al. HASC2011corpus: towards the common ground of human activity recognition. In: Proceedings of the 13th international conference on Ubiquitous computing [Internet]. New York, NY, USA: Association for Computing Machinery; 2011 [cited 2025 Jun 25]. p. 571–2. (UbiComp ’11). Available from: https://dl.acm.org/doi/10.1145/2030112.2030218

25. Li X, Luo J, Younes R. ActivityGAN: generative adversarial networks for data augmentation in sensor-based human activity recognition. In: Adjunct Proceedings of the 2020 ACM International Joint Conference on Pervasive and Ubiquitous Computing and Proceedings of the 2020 ACM International Symposium on Wearable Computers [Internet]. New York, NY, USA: Association for Computing Machinery; 2020 [cited 2025 Apr 17]. p. 249–54. (UbiComp/ISWC ’20 Adjunct). Available from: https://dl.acm.org/doi/10.1145/3410530.3414367

26. Hao L, Bakkes THGF, van Diepen A, Chennakeshava N, Bouwman RA, De Bie Dekker AJR, et al. An adversarial learning approach to generate pressure support ventilation waveforms for asynchrony detection. Comput Methods Programs Biomed. 2024 Jun;250:108175.

27. van Diepen A, Bakkes T, de Bie A, Turco S, Bouwman A, Woerlee P, et al. Simulated data from A Model-based Approach to Generating Annotated Pressure Support Waveforms [Internet]. 4TU.ResearchData; 2024 [cited 2025 Jun 25]. Available from: https://data.4tu.nl/datasets/81220350-0f3d-4e0a-86cf-28c904a1cb09/1

28. Al-Shamsi S, Regmi D, Govender RD. Chronic kidney disease in patients at high risk of cardiovascular disease in the United Arab Emirates: A population-based study. PloS One. 2018;13(6):e0199920.

29. Kuo NIH, Gallego B, Jorm L. Attention-Based Synthetic Data Generation for Calibration-Enhanced Survival Analysis: A Case Study for Chronic Kidney Disease Using Electronic Health Records [Internet]. arXiv; 2025 [cited 2025 Mar 26]. Available from: http://arxiv.org/abs/2503.06096

30. Penzel T, Moody GB, Mark RG, Goldberger AL, Peter JH. Apnea-ECG Database [Internet]. physionet.org; 2000 [cited 2025 Jun 25]. Available from: https://physionet.org/content/apnea-ecg/

31. Penzel T, Moody GB, Mark RG, Goldberger A, Peter JH. The apnea-ECG database. Vol. 27, Computers in Cardiology. 2000. 255 p.

32. Nikolaidis K, Kristiansen S, Goebel V, Plagemann T, Liestøl K, Kankanhalli M. Augmenting Physiological Time Series Data: A Case Study for Sleep Apnea Detection [Internet]. arXiv; 2019 [cited 2025 Apr 17]. Available from: http://arxiv.org/abs/1905.09068

33. Ichimaru Y, Moody GB. MIT-BIH Polysomnographic Database [Internet]. physionet.org; 1992 [cited 2025 Jun 25]. Available from: https://physionet.org/content/slpdb/

34. Kallidromitis K, Gudovskiy D, Kozuka K, Ohama I, Rigazio L. Contrastive Neural Processes for Self-Supervised Learning [Internet]. arXiv; 2021 [cited 2025 Mar 26]. Available from: http://arxiv.org/abs/2110.13623

35. Dissanayake T, Fernando T, Denman S, Sridharan S, Fookes C. Generalized Generative Deep Learning Models for Biosignal Synthesis and Modality Transfer. IEEE J Biomed Health Inform. 2023 Feb;27(2):968–79.

36. Habiba M, Brophy E, Pearlmutter BA, Ward T. ECG synthesis with Neural ODE and GAN models [Internet]. arXiv; 2022 [cited 2025 Mar 26]. Available from: http://arxiv.org/abs/2111.00314

37. Moody GB, Mark RG. MIT-BIH Arrhythmia Database [Internet]. physionet.org; 1992 [cited 2025 Jun 26]. Available from: https://physionet.org/content/mitdb/

38. Torfi A, Fox EA, Reddy CK. Differentially Private Synthetic Medical Data Generation using Convolutional GANs. Inf Sci. 2022 Mar;586:485–500.

39. Zhu F, Ye F, Fu Y, Liu Q, Shen B. Electrocardiogram generation with a bidirectional LSTM-CNN generative adversarial network. Sci Rep. 2019 May;9(1):6734.

40. Wulan N, Wang W, Sun P, Wang K, Xia Y, Zhang H. Generating electrocardiogram signals by deep learning. Neurocomputing. 2020 Sep;404:122–36.

41. Zhu T, Li K, Herrero P, Georgiou P. GluGAN: Generating Personalized Glucose Time Series Using Generative Adversarial Networks. IEEE J Biomed Health Inform. 2023 Oct;27(10):5122–33.

42. Delaney AM, Brophy E, Ward TE. Synthesis of Realistic ECG using Generative Adversarial Networks [Internet]. arXiv; 2019 [cited 2025 Mar 26]. Available from: http://arxiv.org/abs/1909.09150

43. Brophy E. Synthesis of Dependent Multichannel ECG using Generative Adversarial Networks. In: Proceedings of the 29th ACM International Conference on Information &amp; Knowledge Management [Internet]. New York, NY, USA: Association for Computing Machinery; 2020. p. 3229–32. (CIKM ’20). Available from: https://doi.org/10.1145/3340531.3418509

44. Li X, Ngu AHH, Metsis V. TTS-CGAN: A Transformer Time-Series Conditional GAN for Biosignal Data Augmentation [Internet]. arXiv; 2022 [cited 2025 Mar 26]. Available from: http://arxiv.org/abs/2206.13676

45. Li X, Metsis V, Wang H, Ngu AHH. TTS-GAN: A Transformer-based Time-Series Generative Adversarial Network [Internet]. arXiv; 2022 [cited 2025 Mar 26]. Available from: http://arxiv.org/abs/2202.02691

46. Goldberger AL, Amaral LA, Glass L, Hausdorff JM, Ivanov PC, Mark RG, et al. PhysioBank, PhysioToolkit, and PhysioNet: components of a new research resource for complex physiologic signals. Circulation. 2000 Jun 13;101(23):E215-220.

47. Sood M, Suenkel U, Thaler AK von, Zacharias HU, Brockmann K, Eschweiler GW, et al. Bayesian network modeling of risk and prodromal markers of Parkinson’s disease. PLOS ONE. 2023 Feb;18(2):e0280609.

48. Che Z, Cheng Y, Zhai S, Sun Z, Liu Y. Boosting Deep Learning Risk Prediction with Generative Adversarial Networks for Electronic Health Records [Internet]. arXiv; 2017 [cited 2025 Mar 26]. Available from: http://arxiv.org/abs/1709.01648

49. Li H, Yu S, Principe J. Causal Recurrent Variational Autoencoder for Medical Time Series Generation. Proc AAAI Conf Artif Intell. 2023 Jun;37(7):8562–70.

50. NetSim [Internet]. [cited 2025 Jun 25]. Available from: https://www.fmrib.ox.ac.uk/datasets/netsim/

51. Statistical Analysis of Network Data [Internet]. [cited 2025 Jun 25]. Available from: https://math.bu.edu/people/kolaczyk/datasets.html

52. Pang C, Jiang X, Pavinkurve NP, Kalluri KS, Minto EL, Patterson J, et al. CEHR-GPT: Generating Electronic Health Records with Chronological Patient Timelines [Internet]. arXiv; 2024 [cited 2025 Mar 26]. Available from: http://arxiv.org/abs/2402.04400

53. Johnson A, Pollard T, Mark R. MIMIC-III Clinical Database [Internet]. PhysioNet; 2015 [cited 2025 Jun 25]. Available from: https://physionet.org/content/mimiciii/1.4/

54. Sun H, Lin H, Yan R. Collaborative Synthesis of Patient Records through Multi-Visit Health State Inference [Internet]. arXiv; 2023 [cited 2025 Mar 26]. Available from: http://arxiv.org/abs/2312.14646

55. Bing S, Dittadi A, Bauer S, Schwab P. Conditional Generation of Medical Time Series for Extrapolation to Underrepresented Populations [Internet]. arXiv; 2022 [cited 2025 Mar 26]. Available from: http://arxiv.org/abs/2201.08186

56. Theodorou B, Jain S, Xiao C, Sun J. ConSequence: Synthesizing Logically Constrained Sequences for Electronic Health Record Generation. Proc AAAI Conf Artif Intell. 2024 Mar;38(14):15355–63.

57. Wang L, Zhang W, He X. Continuous Patient-Centric Sequence Generation via Sequentially Coupled Adversarial Learning. In: Li G, Yang J, Gama J, Natwichai J, Tong Y, editors. Database Systems for Advanced Applications. Cham: Springer International Publishing; 2019. p. 36–52.

58. Liu Z, Liu X, Zhang X, Li J. Contrastive Learning-Based Time Series Classification in Healthcare. In: Proceedings of the 2023 4th International Symposium on Artificial Intelligence for Medicine Science [Internet]. New York, NY, USA: Association for Computing Machinery; 2024. p. 728–33. (ISAIMS ’23). Available from: https://doi.org/10.1145/3644116.3644238

59. Torfi A, Fox EA. CorGAN: Correlation-Capturing Convolutional Generative Adversarial Networks for Generating Synthetic Healthcare Records [Internet]. arXiv; 2020 [cited 2025 Mar 26]. Available from: http://arxiv.org/abs/2001.09346

60. Yoon J, Mizrahi M, Ghalaty NF, Jarvinen T, Ravi AS, Brune P, et al. EHR-Safe: generating high-fidelity and privacy-preserving synthetic electronic health records. Npj Digit Med. 2023 Aug;6(1):1–11.

61. Tarek MFB, Poulain R, Beheshti R. Fairness-Optimized Synthetic EHR Generation for Arbitrary Downstream Predictive Tasks [Internet]. arXiv; 2024 [cited 2025 Mar 26]. Available from: http://arxiv.org/abs/2406.02510

62. Zhou G, Barbieri S. Generating Clinically Realistic EHR Data via a Hierarchy- and Semantics-Guided Transformer [Internet]. arXiv; 2025 [cited 2025 Mar 26]. Available from: http://arxiv.org/abs/2502.20719

63. Lee D, Yu H, Jiang X, Rogith D, Gudala M, Tejani M, et al. Generating sequential electronic health records using dual adversarial autoencoder. J Am Med Inform Assoc JAMIA. 2020 Jul;27(9):1411–9.

64. Li J, Cairns BJ, Li J, Zhu T. Generating Synthetic Mixed-type Longitudinal Electronic Health Records for Artificial Intelligent Applications [Internet]. arXiv; 2023 [cited 2025 Mar 26]. Available from: http://arxiv.org/abs/2112.12047

65. Brouwer ED, Simm J, Arany A, Moreau Y. GRU-ODE-Bayes: Continuous modeling of sporadically-observed time series [Internet]. arXiv; 2019 [cited 2025 Mar 26]. Available from: http://arxiv.org/abs/1905.12374

66. Silva JF, Matos S. Modelling Patient Trajectories Using Multimodal Information [Internet]. arXiv; 2022 [cited 2025 Mar 26]. Available from: http://arxiv.org/abs/2209.04224

67. Lu C, Reddy CK, Wang P, Nie D, Ning Y. Multi-Label Clinical Time-Series Generation via Conditional GAN. IEEE Trans Knowl Data Eng. 2024 Apr;36(4):1728–40.

68. Wang Z, Sun J. PromptEHR: Conditional Electronic Healthcare Records Generation with Prompt Learning [Internet]. arXiv; 2022 [cited 2025 Apr 2]. Available from: http://arxiv.org/abs/2211.01761

69. Theodorou B, Xiao C, Sun J. Synthesize High-dimensional Longitudinal Electronic Health Records via Hierarchical Autoregressive Language Model. Nat Commun. 2023 Aug;14(1):5305.

70. Zhong Y, Wang X, Wang J, Zhang X, Wang Y, Huai M, et al. Synthesizing Multimodal Electronic Health Records via Predictive Diffusion Models. In: Proceedings of the 30th ACM SIGKDD Conference on Knowledge Discovery and Data Mining [Internet]. New York, NY, USA: Association for Computing Machinery; 2024. p. 4607–18. (KDD ’24). Available from: https://doi.org/10.1145/3637528.3671836

71. Kuo NIH, Jorm L, Barbieri S. Synthetic Health-related Longitudinal Data with Mixed-type Variables Generated using Diffusion Models [Internet]. arXiv; 2023 [cited 2025 Mar 26]. Available from: http://arxiv.org/abs/2303.12281

72. Yalavarthi VK, Burchert J, Schmidt-thieme L. Tripletformer for Probabilistic Interpolation of Irregularly sampled Time Series [Internet]. arXiv; 2024 [cited 2025 Mar 26]. Available from: http://arxiv.org/abs/2210.02091

73. Zhong Y, Cui S, Wang J, Wang X, Yin Z, Wang Y, et al. MedDiffusion: Boosting Health Risk Prediction via Diffusion-based Data Augmentation [Internet]. arXiv; 2023 [cited 2025 Mar 26]. Available from: http://arxiv.org/abs/2310.02520

74. Johnson A, Bulgarelli L, Pollard T, Horng S, Celi LA, Mark R. MIMIC-IV [Internet]. PhysioNet; [cited 2025 Jun 25]. Available from: https://physionet.org/content/mimiciv/1.0/

75. Tian M, Chen B, Guo A, Jiang S, Zhang AR. Reliable Generation of Privacy-preserving Synthetic Electronic Health Record Time Series via Diffusion Models [Internet]. arXiv; 2024 [cited 2025 Mar 26]. Available from: http://arxiv.org/abs/2310.15290

76. Nikolentzos G, Vazirgiannis M, Xypolopoulos C, Lingman M, Brandt EG. Synthetic electronic health records generated with variational graph autoencoders. Npj Digit Med. 2023 Apr;6(1):1–12.

77. Hashemi AS, Etminani K, Soliman A, Hamed O, Lundström J. Time-series Anonymization of Tabular Health Data using Generative Adversarial Network. In: 2023 International Joint Conference on Neural Networks (IJCNN) [Internet]. 2023 [cited 2025 Apr 16]. p. 1–8. Available from: https://ieeexplore.ieee.org/document/10191367

78. Andrzejak RG, Lehnertz K, Mormann F, Rieke C, David P, Elger CE. Indications of nonlinear deterministic and finite-dimensional structures in time series of brain electrical activity: Dependence on recording region and brain state. Phys Rev E. 2001 Nov 20;64(6):061907.

79. Anguita D, Ghio A, Oneto L, Parra F, Reyes-Ortiz J. A Public Domain Dataset for Human Activity Recognition using Smartphones. 2013.

80. IMS Bearings - NASA Open Data Portal [Internet]. [cited 2025 Jun 25]. Available from: https://data.nasa.gov/dataset/ims-bearings

81. Urban Sound Datasets [Internet]. [cited 2025 Jun 25]. UrbanSound8K. Available from: https://urbansounddataset.weebly.com/urbansound8k.html

82. Epileptic Seizure Recognition [Internet]. [cited 2025 Jun 26]. Available from: https://www.kaggle.com/datasets/harunshimanto/epileptic-seizure-recognition

83. Sudlow C, Gallacher J, Allen N, Beral V, Burton P, Danesh J, et al. UK Biobank: An Open Access Resource for Identifying the Causes of a Wide Range of Complex Diseases of Middle and Old Age. PLOS Med. 2015 Mar 31;12(3):e1001779.

84. Choi S, Kim S. Data augmentation method for modeling health records with applications to clopidogrel treatment failure detection [Internet]. arXiv; 2024 [cited 2025 Mar 26]. Available from: http://arxiv.org/abs/2402.18046

85. Gatekeeper Project | GATEKEEPER PROJECT [Internet]. 2022 [cited 2025 Jun 26]. Available from: https://www.gatekeeper-project.eu/

86. Haleem MS, Ekuban A, Antonini A, Pagliara S, Pecchia L, Allocca C. Deep-Learning-Driven Techniques for Real-Time Multimodal Health and Physical Data Synthesis. Electronics. 2023 Jan;12(9):1989.

87. Chang H, Woo SH, Kang S, Lee CY, Lee JY, Ryu JK. A curtailed task for quantitative evaluation of visuomotor adaptation in the head-mounted display virtual reality environment. Front Psychiatry [Internet]. 2023 Feb 16 [cited 2025 Jun 26];13. Available from: https://www.frontiersin.org/journals/psychiatry/articles/10.3389/fpsyt.2022.963303/full

88. Kim J, Woo SH, Kim T, Yoon WT, Shin JH, Lee JY, et al. Development of a cerebellar ataxia diagnosis model using conditional GAN-based synthetic data generation for visuomotor adaptation task. BMC Med Inform Decis Mak. 2024 Nov;24(1):336.

89. Fernandes K, Cardoso J, Fernández J. Transfer Learning with Partial Observability Applied to Cervical Cancer Screening. 2017. 243 p.

90. Bousseljot RD, Kreiseler D, Schnabel A. The PTB Diagnostic ECG Database [Internet]. physionet.org; 2004 [cited 2025 Jun 26]. Available from: https://physionet.org/content/ptbdb/

91. Afrin K, Verma P, Srivatsa SS, Bukkapatnam STS. Simultaneous 12-Lead Electrocardiogram Synthesis using a Single-Lead ECG Signal: Application to Handheld ECG Devices [Internet]. arXiv; 2018 [cited 2025 Mar 26]. Available from: http://arxiv.org/abs/1811.08035

92. Cardiovascular Disease dataset [Internet]. [cited 2025 Jun 26]. Available from: https://www.kaggle.com/datasets/sulianova/cardiovascular-disease-dataset

93. Wagner P, Strodthoff N, Bousseljot RD, Kreiseler D, Lunze FI, Samek W, et al. PTB-XL, a large publicly available electrocardiography dataset. Sci Data. 2020 May 25;7(1):154.

94. Alcaraz JML, Strodthoff N. Diffusion-based Conditional ECG Generation with Structured State Space Models [Internet]. arXiv; 2023 [cited 2025 Mar 26]. Available from: http://arxiv.org/abs/2301.08227

95. Pollard TJ, Johnson AEW, Raffa JD, Celi LA, Mark RG, Badawi O. The eICU Collaborative Research Database, a freely available multi-center database for critical care research. Sci Data. 2018 Sep 11;5(1):180178.

96. Hoorn R van, Bakkes T, Tokoutsi Z, Jong Y de, Bouwman RA, Pechenizkiy M. Generating Privacy-Preserving Longitudinal Synthetic Data. In 2023 [cited 2025 Apr 2]. Available from: https://openreview.net/forum?id=Xr13v66xxT

97. Ghosheh GO, Li J, Zhu T. IGNITE: Individualized GeNeration of Imputations in Time-series Electronic health records [Internet]. arXiv; 2024 [cited 2025 Mar 26]. Available from: http://arxiv.org/abs/2401.04402

98. Hyland SL, Esteban C, Rätsch G. Real-valued (Medical) Time Series Generation with Recurrent Conditional GANs [Internet]. arXiv; 2017 [cited 2025 Mar 26]. Available from: http://arxiv.org/abs/1706.02633

99. Schalk G, Mcfarland D, Hinterberger T, Birbaumer N, Wolpaw J. BCI2000: a general-purpose Brain-Computer Interface (BCI) system. IEEE Trans Biomed Eng. 2004 Jul 1;51:1034.

100. Markova V, Ganchev T, Kalinkov K. CLAS: A Database for Cognitive Load, Affect and Stress Recognition. 2020.

101. Jorge Reyes-Ortiz DA. Human Activity Recognition Using Smartphones [Internet]. UCI Machine Learning Repository; 2013 [cited 2025 Jun 26]. Available from: https://archive.ics.uci.edu/dataset/240

102. Biswal S, Ghosh S, Duke J, Malin B, Stewart W, Sun J. EVA: Generating Longitudinal Electronic Health Records Using Conditional Variational Autoencoders [Internet]. arXiv; 2020 [cited 2025 Mar 26]. Available from: http://arxiv.org/abs/2012.10020

103. Zeng X, Yu G, Lu Y, Tan L, Wu X, Shi S, et al. PIC, a paediatric-specific intensive care database. Sci Data. 2020 Jan 13;7(1):14.

104. Li H, Zeng X, Yu G. Paediatric Intensive Care database [Internet]. PhysioNet; [cited 2025 Jun 26]. Available from: https://physionet.org/content/picdb/1.1.0/

105. Steven Eyobu O, Han DS. Feature Representation and Data Augmentation for Human Activity Classification Based on Wearable IMU Sensor Data Using a Deep LSTM Neural Network. Sensors. 2018 Sep;18(9):2892.

106. Kazemnejad A, Gordany P, Sameni R. EPHNOGRAM: A Simultaneous Electrocardiogram and Phonocardiogram Database [Internet]. PhysioNet; [cited 2025 Jun 26]. Available from: https://physionet.org/content/ephnogram/1.0.0/

107. Shi J, Wang D, Tesei G, Norgeot B. Generating high-fidelity privacy-conscious synthetic patient data for causal effect estimation with multiple treatments. Front Artif Intell [Internet]. 2022 Sep [cited 2025 Apr 16];5. Available from: https://www.frontiersin.orghttps://www.frontiersin.org/journals/artificial-intelligence/articles/10.3389/frai.2022.918813/full

108. Seyfi A, Rajotte JF, Ng RT. Generating multivariate time series with COmmon Source CoordInated GAN (COSCI-GAN) [Internet]. arXiv; 2022 [cited 2025 Mar 26]. Available from: http://arxiv.org/abs/2205.13741

109. Yoon J, Jarrett D, van der Schaar M. Time-series Generative Adversarial Networks. In: Advances in Neural Information Processing Systems [Internet]. Curran Associates, Inc.; 2019 [cited 2025 Jun 26]. Available from: https://papers.nips.cc/paper_files/paper/2019/hash/c9efe5f26cd17ba6216bbe2a7d26d490-Abstract.html

110. Kipnis P, Turk BJ, Wulf DA, LaGuardia JC, Liu V, Churpek MM, et al. Development and validation of an electronic medical record-based alert score for detection of inpatient deterioration outside the ICU. J Biomed Inform. 2016 Dec;64:10–9.

111. hc-24 — CRCNS.org [Internet]. [cited 2025 Jun 26]. Available from: https://crcns.org/data-sets/hc/hc-24

112. Vetter J, Macke JH, Gao R. Generating realistic neurophysiological time series with denoising diffusion probabilistic models. Patterns. 2024 Sep;5(9):101047.

113. Peterson SM, Singh SH, Dichter B, Scheid M, Rao RPN, Brunton BW. AJILE12: Long-term naturalistic human intracranial neural recordings and pose. Sci Data. 2022 Apr 21;9(1):184.

114. Zazzi M, Incardona F, Rosen-Zvi M, Prosperi M, Lengauer T, Altmann A, et al. Predicting response to antiretroviral treatment by machine learning: the EuResist project. Intervirology. 2012;55(2):123–7.

115. Kuo NIH, Garcia F, Sönnerborg A, Böhm M, Kaiser R, Zazzi M, et al. Generating synthetic clinical data that capture class imbalanced distributions with generative adversarial networks: Example using antiretroviral therapy for HIV. J Biomed Inform. 2023 Aug;144:104436.

116. Philip Schmidt AR. WESAD (Wearable Stress and Affect Detection) [Internet]. UCI Machine Learning Repository; 2018 [cited 2025 Jun 26]. Available from: https://archive.ics.uci.edu/dataset/465

117. Lange L, Wenzlitschke N, Rahm E. Generating Synthetic Health Sensor Data for Privacy-Preserving Wearable Stress Detection. Sensors. 2024 Jan;24(10):3052.

118. Yèche H, Kuznetsova R, Zimmermann M, Hüser M, Lyu X, Faltys M, et al. HiRID-ICU-Benchmark -- A Comprehensive Machine Learning Benchmark on High-resolution ICU Data [Internet]. arXiv; 2022 [cited 2025 Jun 26]. Available from: http://arxiv.org/abs/2111.08536

119. Home | Parkinson’s Progression Markers Initiative [Internet]. [cited 2025 Jun 27]. Available from: https://www.ppmi-info.org/

120. Wendland P, Birkenbihl C, Gomez-Freixa M, Sood M, Kschischo M, Fröhlich H. Generation of realistic synthetic data using Multimodal Neural Ordinary Differential Equations. Npj Digit Med. 2022 Aug;5(1):1–10.

121. Sood M, Sahay A, Karki R, Emon MA, Vrooman H, Hofmann-Apitius M, et al. Realistic simulation of virtual multi-scale, multi-modal patient trajectories using Bayesian networks and sparse auto-encoders. Sci Rep. 2020 Jul;10(1):10971.

122. Gootjes-Dreesbach L, Sood M, Sahay A, Hofmann-Apitius M, Fröhlich H. Variational Autoencoder Modular Bayesian Networks for Simulation of Heterogeneous Clinical Study Data. Front Big Data [Internet]. 2020 May [cited 2025 Apr 2];3. Available from: https://www.frontiersin.org/journals/big-data/articles/10.3389/fdata.2020.00016/full

123. NACC Home | National Alzheimer’s Coordinating Center [Internet]. [cited 2025 Jun 27]. Available from: https://naccdata.org/

124. Drouin P, Stamm A, Chevreuil L, Graillot V, Barbin L, Gourraud PA, et al. Semi-supervised clustering of quaternion time series: Application to gait analysis in multiple sclerosis using motion sensor data. Stat Med. 2023 Feb 20;42(4):433–56.

125. Gall KL, Bellanger L, Laplaud D, Stamm A. Generation of synthetic gait data: application to multiple sclerosis patients’ gait patterns [Internet]. arXiv; 2024 [cited 2025 Mar 26]. Available from: http://arxiv.org/abs/2411.10377

126. Linial O, Ravid N, Eytan D, Shalit U. Generative ODE modeling with known unknowns. In: Proceedings of the Conference on Health, Inference, and Learning [Internet]. New York, NY, USA: Association for Computing Machinery; 2021. p. 79–94. (CHIL ’21). Available from: https://doi.org/10.1145/3450439.3451866

127. Information (NCEI) NC for E. United States Historical Climatology Network (USHCN), Version 2.5 [Internet]. [cited 2025 Jun 27]. Available from: https://www.ncei.noaa.gov/access/metadata/landing-page/bin/iso?id=gov.noaa.ncdc:C00895

128. Predicting Mortality of ICU Patients: The PhysioNet/Computing in Cardiology Challenge 2012 v1.0.0 [Internet]. [cited 2025 Jun 27]. Available from: https://www.physionet.org/content/challenge-2012/1.0.0/

129. Ramchandran S, Tikhonov G, Kujanpää K, Koskinen M, Lähdesmäki H. Longitudinal Variational Autoencoder [Internet]. arXiv; 2021 [cited 2025 Mar 26]. Available from: http://arxiv.org/abs/2006.09763

130. Reyna MA, Josef CS, Jeter R, Shashikumar SP, Westover MB, Nemati S, et al. Early Prediction of Sepsis From Clinical Data: The PhysioNet/Computing in Cardiology Challenge 2019. Crit Care Med. 2020 Feb;48(2):210–7.

131. Kiyasseh D, Tadesse GA, Nhan LNT, Van Tan L, Thwaites L, Zhu T, et al. PlethAugment: GAN-Based PPG Augmentation for Medical Diagnosis in Low-Resource Settings. IEEE J Biomed Health Inform. 2020 Nov;24(11):3226–35.

132. De Vito S, Massera E, Piga M, Martinotto L, Di Francia G. On field calibration of an electronic nose for benzene estimation in an urban pollution monitoring scenario. Sens Actuators B Chem. 2008 Feb 22;129(2):750–7.

133. Lee M, Tae D, Choi JH, Jung HY, Seok J. Improved recurrent generative adversarial networks with regularization techniques and a controllable framework. Inf Sci. 2020 Oct;538:428–43.

134. Liang X, Zou T, Guo B, Li S, Zhang H, Zhang S, et al. Assessing Beijing’s PM2.5 pollution: severity, weather impact, APEC and winter heating. Proc R Soc Math Phys Eng Sci. 2015 Oct 8;471(2182):20150257.

135. Fallahzadeh R, Bidoki NH, Stelzer IA, Becker M, Marić I, Chang AL, et al. In-silico generation of high-dimensional immune response data in patients using a deep neural network. Cytometry A. 2023;103(5):392–404.

136. Alt EM, Qu Y, Damone E, Liu J ou, Wang C, Ibrahim JG. Jointly modeling time-to-event and longitudinal data with individual-specific change points: a case study in modeling tumor burden [Internet]. arXiv; 2024 [cited 2025 Mar 26]. Available from: http://arxiv.org/abs/2409.13873

137. Roden DM, Pulley JM, Basford MA, Bernard GR, Clayton EW, Balser JR, et al. Development of a large-scale de-identified DNA biobank to enable personalized medicine. Clin Pharmacol Ther. 2008 Sep;84(3):362–9.

138. Zhang Z, Yan C, Malin BA. Keeping synthetic patients on track: feedback mechanisms to mitigate performance drift in longitudinal health data simulation. J Am Med Inform Assoc. 2022 Nov;29(11):1890–8.

139. Healthcare Cost and Utilization Project (HCUP) [Internet]. [cited 2025 Jun 27]. Available from: https://www.ahrq.gov/data/hcup/index.html

140. Lecun Y, Bottou L, Bengio Y, Haffner P. Gradient-based learning applied to document recognition. Proc IEEE. 1998 Nov;86(11):2278–324.

141. Ramchandran S, Tikhonov G, Lönnroth O, Tiikkainen P, Lähdesmäki H. Learning Conditional Variational Autoencoders with Missing Covariates [Internet]. arXiv; 2022 [cited 2025 Mar 26]. Available from: http://arxiv.org/abs/2203.01218

142. Green AK, Reeder-Hayes KE, Corty RW, Basch E, Milowsky MI, Dusetzina SB, et al. The Project Data Sphere Initiative: Accelerating Cancer Research by Sharing Data. The Oncologist. 2015 May;20(5):464-e20.

143. Schulz NA, Carus J, Wiederhold AJ, Johanns O, Peters F, Rath N, et al. Learning debiased graph representations from the OMOP common data model for synthetic data generation. BMC Med Res Methodol. 2024 Jun;24(1):136.

144. Romero K, de Mars M, Frank D, Anthony M, Neville J, Kirby L, et al. The Coalition Against Major Diseases: developing tools for an integrated drug development process for Alzheimer’s and Parkinson’s diseases. Clin Pharmacol Ther. 2009 Oct;86(4):365–7.

145. Fisher CK, Smith AM, Walsh JR. Machine learning for comprehensive forecasting of Alzheimer’s Disease progression. Sci Rep. 2019 Sep;9(1):13622.

146. TriNetX [Internet]. [cited 2025 Jun 27]. Real-world data for the life sciences and healthcare. Available from: https://trinetx.com/

147. Dau HA, Bagnall A, Kamgar K, Yeh CCM, Zhu Y, Gharghabi S, et al. The UCR Time Series Archive [Internet]. arXiv; 2019 [cited 2025 Jun 27]. Available from: http://arxiv.org/abs/1810.07758

148. Wickstrøm K, Kampffmeyer M, Mikalsen KØ, Jenssen R. Mixing Up Contrastive Learning: Self-Supervised Representation Learning for Time Series. Pattern Recognit Lett. 2022 Mar;155:54–61.

149. Bagnall A, Dau HA, Lines J, Flynn M, Large J, Bostrom A, et al. The UEA multivariate time series classification archive, 2018 [Internet]. arXiv; 2018 [cited 2025 Jun 27]. Available from: http://arxiv.org/abs/1811.00075

150. Huang SS, Singh R, McKinnell JA, Park S, Gombosev A, Eells SJ, et al. Decolonization to Reduce Postdischarge Infection Risk among MRSA Carriers. N Engl J Med. 2019 Feb 14;380(7):638–50.

151. Poyraz O, Marttinen P. Mixture of Coupled HMMs for Robust Modeling of Multivariate Healthcare Time Series [Internet]. arXiv; 2023 [cited 2025 Mar 26]. Available from: http://arxiv.org/abs/2311.07867

152. Ceritli T. tahaceritli/mIOHMM [Internet]. 2025 [cited 2025 Jun 27]. Available from: https://github.com/tahaceritli/mIOHMM

153. Ceritli T, Creagh AP, Clifton DA. Mixture of Input-Output Hidden Markov Models for Heterogeneous Disease Progression Modeling [Internet]. arXiv; 2022 [cited 2025 Mar 26]. Available from: http://arxiv.org/abs/2207.11846

154. Nazabal A, Olmos PM, Ghahramani Z, Valera I. Handling Incomplete Heterogeneous Data using VAEs [Internet]. arXiv; 2020 [cited 2025 Jun 27]. Available from: http://arxiv.org/abs/1807.03653

155. Moazemi S, Adams T, NG HG, Kühnel L, Schneider J, Näher AF, et al. NFDI4Health workflow and service for synthetic data generation, assessment and risk management [Internet]. arXiv; 2024 [cited 2025 Apr 2]. Available from: http://arxiv.org/abs/2408.04478

156. Alzheimer’s Disease Neuroimaging Initiative [Internet]. ADNI. [cited 2025 Jun 27]. Available from: https://adni-lde.loni.usc.edu/

157. PPG-BP Database [Internet]. figshare; 2018 [cited 2025 Jun 27]. Available from: https://figshare.com/articles/dataset/PPG-BP_Database_zip/5459299/5

158. Li K, Yang S, Sullivan TM, Burd RS, Marsic I. ProcessGAN: Generating Privacy-Preserving Time-Aware Process Data with Conditional Generative Adversarial Nets. ACM Trans Knowl Discov Data [Internet]. 2024 Nov;18(9). Available from: https://doi.org/10.1145/3687464

159. Cha J, Voigt-Antons JN, Trahms C, O’Sullivan JL, Gellert P, Kuhlmey A, et al. Finding critical features for predicting quality of life in tablet-based serious games for dementia. Qual User Exp. 2019 Nov 5;4(1):6.

160. Ashrafi N, Schmitt V, Spang RP, Möller S, Voigt-Antons JN. Protect and Extend – Using GANs for Synthetic Data Generation of Time-Series Medical Records [Internet]. arXiv; 2024 [cited 2025 Mar 26]. Available from: http://arxiv.org/abs/2402.14042

161. Stocks 🤑 Generate Synthetic Data TimeGAN [Internet]. [cited 2025 Jun 27]. Available from: https://kaggle.com/code/alincijov/stocks-generate-synthetic-data-timegan

162. Yadav P, Gaur M, Fatima N, Sarwar S. Qualitative and Quantitative Evaluation of Multivariate Time-Series Synthetic Data Generated Using MTS-TGAN: A Novel Approach. Appl Sci. 2023 Jan;13(7):4136.

163. Micucci D, Mobilio M, Napoletano P. UniMiB SHAR: A Dataset for Human Activity Recognition Using Acceleration Data from Smartphones. Appl Sci. 2017 Oct;7(10):1101.

164. Yu K, Wang Y, Cai Y, Xiao C, Zhao E, Glass L, et al. Rare Disease Detection by Sequence Modeling with Generative Adversarial Networks [Internet]. arXiv; 2019 [cited 2025 Apr 16]. Available from: http://arxiv.org/abs/1907.01022

165. MNIST Dataset [Internet]. [cited 2025 Jun 27]. Available from: https://www.kaggle.com/datasets/hojjatk/mnist-dataset

166. George B. Moody PhysioNet Challenge [Internet]. [cited 2025 Jun 27]. QT Interval Measurement: The PhysioNet/Computing in Cardiology Challenge 2006. Available from: https://moody-challenge.physionet.org/2006/

167. Sørensen K, Diez P, Margeta J, Youssef YE, Pham M, Pedersen JJ, et al. Spatio-temporal neural distance fields for conditional generative modeling of the heart [Internet]. arXiv; 2024 [cited 2025 Mar 26]. Available from: http://arxiv.org/abs/2407.10663

168. Birjandtalab J, Cogan D, Maziyar Baran Pouyan, Nourani M. A Non-EEG Dataset for Assessment of Neurological Status [Internet]. physionet.org; 2017 [cited 2025 Jun 27]. Available from: https://physionet.org/content/noneeg/

169. Hsieh TY, Sun Y, Tang X, Wang S, Honavar VG. SrVARM: State Regularized Vector Autoregressive Model for Joint Learning of Hidden State Transitions and State-Dependent Inter-Variable Dependencies from Multi-variate Time Series. In: Proceedings of the Web Conference 2021 [Internet]. New York, NY, USA: Association for Computing Machinery; 2021. p. 2270–80. (WWW ’21). Available from: https://doi.org/10.1145/3442381.3450116

170. Ang KK, Chin ZY, Wang C, Guan C, Zhang H. Filter Bank Common Spatial Pattern Algorithm on BCI Competition IV Datasets 2a and 2b. Front Neurosci [Internet]. 2012 Mar 29 [cited 2025 Jun 27];6. Available from: https://www.frontiersin.org/journals/neuroscience/articles/10.3389/fnins.2012.00039/full

171. Ming Y, Ding W, Pelusi D, Wu D, Wang YK, Prasad M, et al. Subject adaptation network for EEG data analysis. Appl Soft Comput. 2019 Nov;84:105689.

172. Song Z, Toral XS, Xu Y, Liu A, Guo L, Powell G, et al. Supervised multi-specialist topic model with applications on large-scale electronic health record data. In: Proceedings of the 12th ACM International Conference on Bioinformatics, Computational Biology, and Health Informatics [Internet]. New York, NY, USA: Association for Computing Machinery; 2021. (BCB ’21). Available from: https://doi.org/10.1145/3459930.3469543

173. A controlled trial of two nucleoside analogues plus indinavir in persons with human immunodeficiency virus infection and CD4 cell counts of 200 per cubic millimeter or less. AIDS Clinical Trials Group 320 Study Team - PubMed [Internet]. [cited 2025 Jun 27]. Available from: https://pubmed.ncbi.nlm.nih.gov/9287227/

174. Norcliffe A, Cebere B, Imrie F, Lio P, Schaar M van der. SurvivalGAN: Generating Time-to-Event Data for Survival Analysis [Internet]. arXiv; 2023 [cited 2025 Mar 26]. Available from: http://arxiv.org/abs/2302.12749

175. SEER [Internet]. [cited 2025 Jun 27]. Surveillance, Epidemiology, and End Results Program. Available from: https://seer.cancer.gov/index.html

176. Pereira B, Chin SF, Rueda OM, Vollan HKM, Provenzano E, Bardwell HA, et al. The somatic mutation profiles of 2,433 breast cancers refine their genomic and transcriptomic landscapes. Nat Commun. 2016 May 10;7(1):11479.

177. Detti P. Siena Scalp EEG Database [Internet]. PhysioNet; [cited 2025 Jun 27]. Available from: https://physionet.org/content/siena-scalp-eeg/1.0.0/

178. Pimentel MAF, Johnson AEW, Charlton PH, Birrenkott D, Watkinson PJ, Tarassenko L, et al. Toward a Robust Estimation of Respiratory Rate From Pulse Oximeters. IEEE Trans Biomed Eng. 2017 Aug;64(8):1914–23.

179. Denny JC, Bastarache L, Ritchie MD, Carroll RJ, Zink R, Mosley JD, et al. Systematic comparison of phenome-wide association study of electronic medical record data and genome-wide association study data. Nat Biotechnol. 2013 Dec;31(12):1102–10.

180. Evaluating phecodes, clinical classification software, and ICD-9-CM codes for phenome-wide association studies in the electronic health record - PubMed [Internet]. [cited 2025 Jun 27]. Available from: https://pubmed.ncbi.nlm.nih.gov/28686612/

181. Zhang Z, Yan C, Lasko TA, Sun J, Malin BA. SynTEG: a framework for temporal structured electronic health data simulation. J Am Med Inform Assoc JAMIA. 2021 Mar;28(3):596–604.

182. Project Data Sphere [Internet]. [cited 2025 Jun 28]. Available from: https://data.projectdatasphere.org/projectdatasphere/html/content/127

183. Foomani FH, Anisuzzaman DM, Niezgoda J, Niezgoda J, Guns W, Gopalakrishnan S, et al. Synthesizing time-series wound prognosis factors from electronic medical records using generative adversarial networks. J Biomed Inform. 2022 Jan;125:103972.

184. Perrar I, Schadow AM, Schmitting S, Buyken AE, Alexy U. Time and Age Trends in Free Sugar Intake from Food Groups among Children and Adolescents between 1985 and 2016. Nutrients. 2019 Dec 20;12(1):20.

185. Kühnel L, Schneider J, Perrar I, Adams T, Moazemi S, Prasser F, et al. Synthetic data generation for a longitudinal cohort study – evaluation, method extension and reproduction of published data analysis results. Sci Rep. 2024 Jun;14(1):14412.

186. Electroretinogram raw waveforms for control and autism [Internet]. Flinders University; 2022 [cited 2025 Jun 28]. Available from: https://open.flinders.edu.au/articles/dataset/Electroretinogram_raw_waveforms_for_control_and_autism/21546210/1

187. Kulyabin M, Constable PA, Zhdanov A, Lee IO, Skuse DH, Thompson DA, et al. Synthetic Electroretinogram Signal Generation Using Conditional Generative Adversarial Network for Enhancing Classification of Autism Spectrum Disorder [Internet]. arXiv; 2024 [cited 2025 Mar 26]. Available from: http://arxiv.org/abs/2407.08166

188. Bureau of Labor Statistics [Internet]. [cited 2025 Jun 28]. ATUS home. Available from: https://www.bls.gov/tus/

189. Dash S, Dutta R, Guyon I, Pavao A, Yale A, Bennett KP. Synthetic Event Time Series Health Data Generation [Internet]. arXiv; 2019 [cited 2025 Mar 26]. Available from: http://arxiv.org/abs/1911.06411

190. Parbhoo S, Bogojeska J, Zazzi M, Roth V, Doshi-Velez F. Combining Kernel and Model Based Learning for HIV Therapy Selection. AMIA Summits Transl Sci Proc. 2017 Jul 26;2017:239–48.

191. Mongin D, García Romero J, Alvero Cruz JR. Treadmill Maximal Exercise Tests from the Exercise Physiology and Human Performance Lab of the University of Malaga [Internet]. PhysioNet; [cited 2025 Jun 28]. Available from: https://physionet.org/content/treadmill-exercise-cardioresp/1.0.1/

192. Larrea X, Hernandez M, Epelde G, Beristain A, Molina C, Alberdi A, et al. Synthetic Subject Generation with Coupled Coherent Time Series Data. Eng Proc. 2022;18(1):7.

193. Yelp, Inc. Profile | Kaggle [Internet]. [cited 2025 Jun 28]. Available from: https://www.kaggle.com/organizations/yelp-dataset/datasets

194. Dubey M, Srijith PK, Desarkar MS. Time-to-Event Modeling with Hypernetwork based Hawkes Process. In: Proceedings of the 29th ACM SIGKDD Conference on Knowledge Discovery and Data Mining [Internet]. New York, NY, USA: Association for Computing Machinery; 2023. p. 3956–65. (KDD ’23). Available from: https://doi.org/10.1145/3580305.3599912

195. SNAP: Network datasets: 96 million Memetracker memes [Internet]. [cited 2025 Jun 28]. Available from: https://snap.stanford.edu/data/memetracker9.html

196. Lee G, Yu W, Shin K, Cheng W, Chen H. TimeCAP: Learning to Contextualize, Augment, and Predict Time Series Events with Large Language Model Agents [Internet]. arXiv; 2025 [cited 2025 Mar 26]. Available from: http://arxiv.org/abs/2502.11418

197. Zhou H, Zhang S, Peng J, Zhang S, Li J, Xiong H, et al. Informer: Beyond Efficient Transformer for Long Sequence Time-Series Forecasting. Proc AAAI Conf Artif Intell. 2021 May 18;35(12):11106–15.

198. Lai G, Chang WC, Yang Y, Liu H. Modeling Long- and Short-Term Temporal Patterns with Deep Neural Networks. 2018. 95 p.

199. Index of /data/local-climatological-data [Internet]. [cited 2025 Jun 29]. Available from: https://www.ncei.noaa.gov/data/local-climatological-data/

200. Kwapisz JR, Weiss GM, Moore SA. Activity recognition using cell phone accelerometers. SIGKDD Explor Newsl. 2011 März;12(2):74–82.

201. Andrzejak R, Lehnertz K, Mormann F, Rieke C, David P, Elger C. Indications of nonlinear deterministic and finite-dimensional structures in time series of brain electrical activity: Dependence on recording region and brain state. Phys Rev E Stat Nonlin Soft Matter Phys. 2002 Jan 1;64:061907.

202. ALİMOĞLU F, ALPAYDIN E. Combining Multiple Representations for Pen-based Handwritten Digit Recognition. Turk J Electr Eng Comput Sci. 2001 Jan 1;9(1):1–12.

203. Blankertz B, Curio G, Müller KR. Classifying Single Trial EEG: Towards Brain Computer Interfacing. In: Advances in Neural Information Processing Systems [Internet]. MIT Press; 2001 [cited 2025 Jun 29]. Available from: https://papers.nips.cc/paper_files/paper/2001/hash/2d579dc29360d8bbfbb4aa541de5afa9-Abstract.html

204. Qian J, Xie B, Wan B, Li M, Sun M, Chiang PY. TimeLDM: Latent Diffusion Model for Unconditional Time Series Generation [Internet]. arXiv; 2024 [cited 2025 Mar 26]. Available from: http://arxiv.org/abs/2407.04211

205. Tunyasuvunakool S, Muldal A, Doron Y, Liu S, Bohez S, Merel J, et al. dm_control: Software and tasks for continuous control. Softw Impacts. 2020 Nov 1;6:100022.

206. Desai A, Freeman C, Wang Z, Beaver I. TimeVAE: A Variational Auto-Encoder for Multivariate Time Series Generation [Internet]. arXiv; 2021 [cited 2025 Jun 29]. Available from: http://arxiv.org/abs/2111.08095

207. Smith SM, Miller KL, Salimi-Khorshidi G, Webster M, Beckmann CF, Nichols TE, et al. Network modelling methods for FMRI. NeuroImage. 2011 Jan 15;54(2):875–91.

208. Gao C, Beigi M, Shafquat A, Aptekar J, Sun J. TrialSynth: Generation of Synthetic Sequential Clinical Trial Data [Internet]. arXiv; 2024 [cited 2025 Mar 26]. Available from: http://arxiv.org/abs/2409.07089

209. Ruiz AP, Flynn M, Large J, Middlehurst M, Bagnall A. The great multivariate time series classification bake off: a review and experimental evaluation of recent algorithmic advances. Data Min Knowl Discov. 2021 Mar 1;35(2):401–49.

210. Yang Z, Li Y, Zhou G. TS-GAN: Time-series GAN for Sensor-based Health Data Augmentation. ACM Trans Comput Healthc [Internet]. 2023 Apr;4(2). Available from: https://doi.org/10.1145/3583593

211. Banos O, Villalonga C, Garcia R, Saez A, Damas M, Holgado-Terriza JA, et al. Design, implementation and validation of a novel open framework for agile development of mobile health applications. Biomed Eng Online. 2015;14 Suppl 2(Suppl 2):S6.

212. Nutzung der EKG-Signaldatenbank CARDIODAT der PTB über das Internet. Biomed Tech. 2009 Jul 17;40:317–8.

213. Wang Y, Fu T, Xu Y, Ma Z, Xu H, Du B, et al. TWIN-GPT: Digital Twins for Clinical Trials via Large Language Model. ACM Trans Multimed Comput Commun Appl [Internet]. 2024 Jul; Available from: https://doi.org/10.1145/3674838

214. Fu T, HUANG K, Sun J. Automated prediction of clinical trial outcome [Internet]. US20230034559A1, 2023 [cited 2025 Jun 29]. Available from: https://patents.google.com/patent/US20230034559A1/en

215. Das T, Wang Z, Sun J. TWIN: Personalized Clinical Trial Digital Twin Generation. In: Proceedings of the 29th ACM SIGKDD Conference on Knowledge Discovery and Data Mining [Internet]. New York, NY, USA: Association for Computing Machinery; 2023 [cited 2025 Apr 16]. p. 402–13. (KDD ’23). Available from: https://dl.acm.org/doi/10.1145/3580305.3599534

216. Li J, Peng J, Li H, Chen L. UniCL: A Universal Contrastive Learning Framework for Large Time Series Models [Internet]. arXiv; 2024 [cited 2025 Mar 26]. Available from: http://arxiv.org/abs/2405.10597

217. Max-Planck-Institut fuer Biogeochemie - Wetterdaten [Internet]. [cited 2025 Jun 29]. Available from: https://www.bgc-jena.mpg.de/wetter/

218. Trindade A. ElectricityLoadDiagrams20112014 [Internet]. UCI Machine Learning Repository; 2015 [cited 2025 Jun 29]. Available from: https://archive.ics.uci.edu/dataset/321

219. National, Regional, and State Level Outpatient Illness and Viral Surveillance [Internet]. [cited 2025 Jun 29]. Available from: https://gis.cdc.gov/grasp/fluview/fluportaldashboard.html

220. Wang X, Lin Y, Xiong Y, Zhang S, He Y, He Y, et al. Using an optimized generative model to infer the progression of complications in type 2 diabetes patients. BMC Med Inform Decis Mak. 2022 Jul;22(1):174.

221. Moore JB, Stackhouse HP, Fulcher BD, Mahmoodian S. Using matrix-product states for time-series machine learning [Internet]. arXiv; 2024 [cited 2025 Mar 26]. Available from: http://arxiv.org/abs/2412.15826

222. Time Series Classification Website [Internet]. [cited 2025 Jun 29]. Available from: https://www.timeseriesclassification.com/description.php?Dataset=ECG200

223. Barbara NH, Bedding TR, Fulcher BD, Murphy SJ, Van Reeth T. Classifying Kepler light curves for 12 000 A and F stars using supervised feature-based machine learning. Mon Not R Astron Soc. 2022 Aug 1;514(2):2793–804.

224. Arabi D, Bakhshaliyev J, Coskuner A, Madhusudhanan K, Uckardes KS. Wave-Mask/Mix: Exploring Wavelet-Based Augmentations for Time Series Forecasting [Internet]. arXiv; 2024 [cited 2025 Mar 26]. Available from: http://arxiv.org/abs/2408.10951

225. Dahmen J, Cook D. SynSys: A Synthetic Data Generation System for Healthcare Applications. Sensors. 2019 Jan;19(5):1181.

226. Walonoski J, Kramer M, Nichols J, Quina A, Moesel C, Hall D, et al. Synthea: An approach, method, and software mechanism for generating synthetic patients and the synthetic electronic health care record. J Am Med Inform Assoc. 2018 Mar;25(3):230–8.
